# Post‐Synthetic Modification of Porous Organic Cages for Enhanced Iodine Adsorption Performance

**DOI:** 10.1002/advs.202408494

**Published:** 2024-10-14

**Authors:** Qianqian Mao, Siyuan Yang, Jinjin Zhang, Yuanhan Liu, Ming Liu

**Affiliations:** ^1^ Department of Chemistry Zhejiang University Hangzhou 310058 China; ^2^ Hangzhou Global Scientific and Technological Innovation Center (HIC) Zhejiang University Hangzhou 311215 China

**Keywords:** Decontamination, Iodine adsorption, Porous organic cages, Post‐synthetic modification

## Abstract

The capture of radioactive iodine species from nuclear waste is crucial for environmental protection and human health. Porous organic cages (POCs), an emerging porous material, have showed potential in iodine adsorption due to the advantages of tunable pores and processibility. However, integrating multiple desirable characteristics into a single POC through bottom‐up assembly of pre‐designed building blocks remains challenging. Post‐synthetic modification (PSM) offers an alternative approach, enabling the introduction of various functions into a single POC. Herein, a viable and highly efficient three‐step PSM strategy to modify a representative POC (CC3), is presented. The modified POC, **OFT‐RCC3^6+^6Br^−^**, features a charged confined space, electron‐rich heteroatom, and halide ions, exhibiting significantly enhanced iodine vapor uptake compared to the parental cage. The universality of the PSM strategy has been verified by successfully modifying two other POCs. The iodine adsorption behaviors of three modified cage adsorbents in organic solvent and aqueous solution have also been investigated, all of which exhibited improved performance, especially in comparison to ionic cages modified through direct protonation. This work provides an effective PSM strategy for POCs to facilitate iodine adsorption. More importantly, the introduction of a new PSM strategy enriches the functional diversity of POCs, potentially broadening their future applications.

## Graphical abstract

Here a highly efficient post‐synthetic modification (PSM) strategy is developed to modify the structure of porous organic cages (POCs) for enhanced I_2_ adsorption performance by integrating multiple desirable structural features, including electron‐rich atoms, positively charged skeletons, and halide ions. Compared with its parental imine cage, the I_2_ vapor uptake of the modified cage has been improved by 5.4 times, with the adsorption equilibrium time reduced by 40 times.

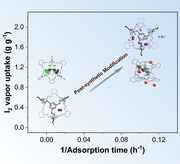

## 1 Introduction

Nuclear energy, a potential solution to meet the increasingly growing global energy demand, has garnered extensive attention due to its high energy density, low emissions, and low operating costs. However, the disposal of nuclear waste poses a significant threat to both human health and the environment. Among the hazardous pollutants, radioactive iodine is of particular concern due to its fast diffusion, high volatility, and severe safety implications. The isotopes of radioactive iodine, such as ^129^I with an extremely long half‐life (≈10^7^ years) and ^131^I with intense radiation, present serious risks to human metabolism and the ecosystem.^[^ ^1^ ^]^

**Scheme 1:**
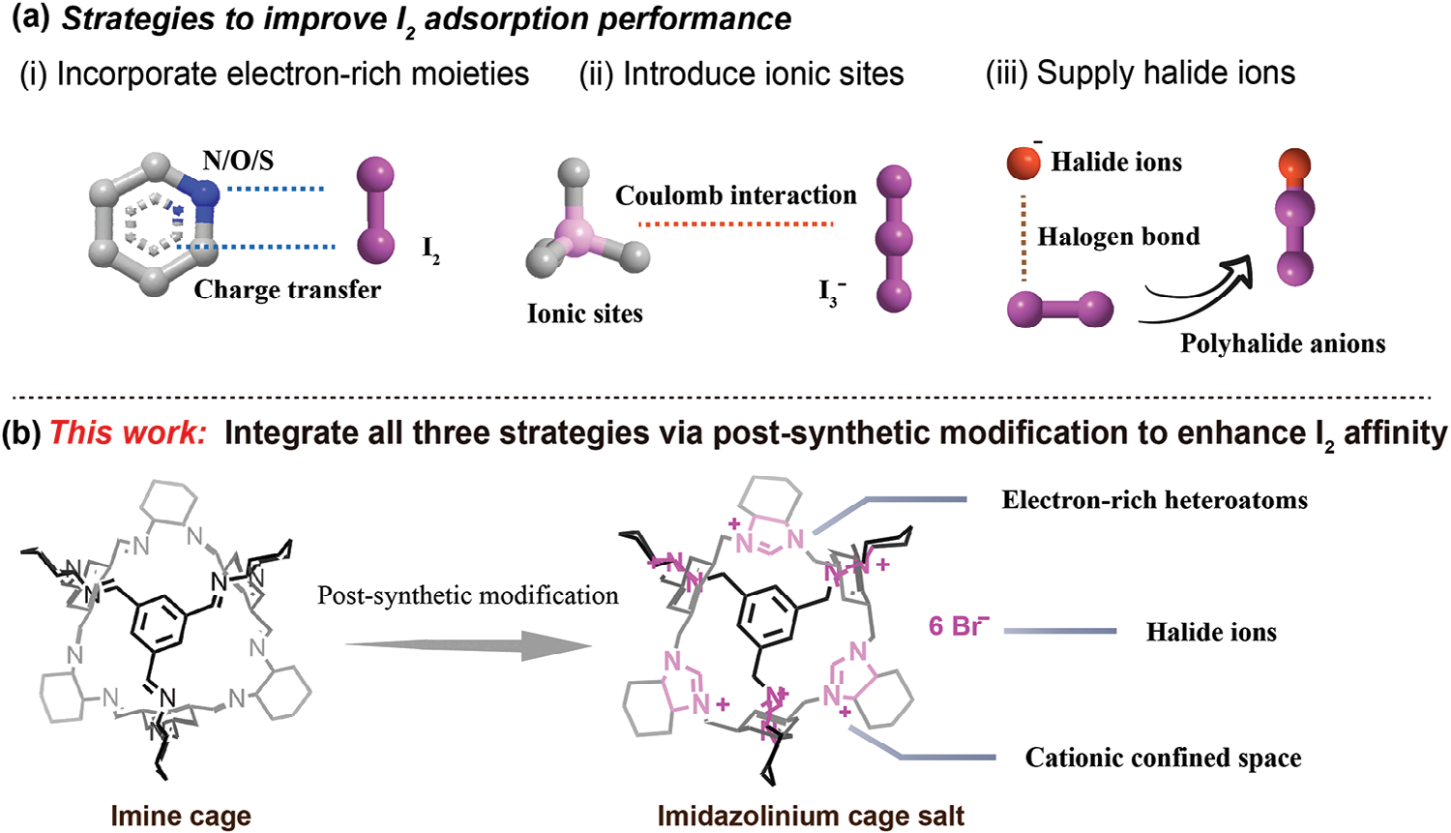
a) Summary of strategies to improve I_2_ adsorption performance in previous works. b) Enhance I_2_ affinity of the porous organic cage through post‐synthetic modification (this work).

Therefore, it is of urgent need to develop adsorbents to capture iodine effectively. Inorganic solid adsorbents represented by Ag‐loaded zeolites^[^ ^2^ ^]^ and activated carbon^[^ ^3^ ^]^ are prevalent in the industry, but limited I_2_ uptake capacity and poor recyclability have restricted their further application. In recent years, various porous materials with good iodine adsorption performance have emerged, such as metal‐organic frameworks (MOFs),^[^ ^4^ ^]^ covalent organic frameworks (COFs),^[^ ^5^ ^]^ porous organic polymers (POPs),^[^ ^6^ ^]^ and macrocycle.^[^ ^7^ ^]^ In general, three effective strategies have been developed and identified to enhance the affinity toward iodine (**Scheme** **1**a): (i) Incorporating electron‐rich moieties (e.g., heteroatoms (N, O, S) or π systems (aromatic rings, double/triple bonds)) to adsorb electron‐deficient I_2_ by forming charge‐transfer complexes.^[^ ^5^, ^8^ ^]^ (ii) Introducing ionic sites to the adsorbents, which can subsequently bind to negatively charged I_2_ species (I_3_^−^ or I_5_^−^) via Coulomb interactions.^[^ ^5^, ^9^ ^]^ (iii) Supplying halide ions to capture I_2_ molecules through the formation of polyhalide anions, followed by electrostatic interactions between complex anions and cation skeletons as the additional driving force.^[^ ^9^, ^10^ ^]^

In comparison with framework materials such as MOFs, COFs, and POPs, discrete porous materials porous organic cages (POCs)^[^ ^8^, ^11^ ^]^ are less investigated for I_2_ capture, possibly due to its relatively lower surface areas. As a new subclass of emerging porous materials with intrinsic porosity, POCs have exhibited remarkable potential in applications including gas storage, molecular recognition, separation, and catalysis.^[^ ^12^ ^]^ The solution processability allows them to be processed into devices, making them suitable for real‐world applications. Additionally, due to their good solubility in common organic solvents, POCs can undergo various chemical reactions, allowing for the introduction or modification of functional groups to tune their properties for specific applications.^[^ ^12^, ^13^ ^]^ Recently, an increasing number of studies aim to improve I_2_ adsorption through the construction or functionalization of POCs. For example, Jiang et al.^[^ ^5^, ^8^ ^]^ reported two [3+6] topological POCs (BTPOC, BPPOC), both of which possessed electron‐rich heterocyclics (thiophene, pyridine) and imine bonds, endowing them with significantly high I_2_ vapor uptake of up to 3.21 and 5.64 g g^−1^, respectively. Sun et al.^[^ ^9a^ ^]^ devised hexacationic imidazolium organic cages bearing different counter anions including halide anions. The excellent iodine capture performance of the 3·6Br^−^ (5.89 g g^−1^) confirmed that introducing charged species into the structures is conducive to optimizing I_2_ binding.

However, it is highly challenging to integrate multiple structural features, such as those facilitating iodine adsorption, onto a single POC via predesign of the building blocks followed by bottom‐up assembly. Subtle changes in the building blocks can profoundly affect the assembly of the target POCs. The post‐synthetic modification (PSM) strategy has proven a powerful tool to enrich the functions of crystalline framework materials such as MOFs^[^ ^14^ ^]^ and COFs.^[^ ^15^ ^]^ It has also been successfully used for tuning the properties of POCs as well as introducing functional groups that are otherwise difficult to be synthesized directly.^[^ ^12^, ^13^, ^16^ ^]^ Therefore, here we aim to develop a PSM strategy that can incorporate all the desirable structural features into a single POC.

**CC3**, a representative [4+6] imine‐based POCs, was studied for iodine capture back in 2011.^[^ ^11^, ^17^ ^]^ As an adsorbent, **CC3** could be easily scaled up and showed good stability. However, the relatively low iodine adsorption capacity of 0.364 g g^−1^ and long adsorption equilibrium time of 350 h hindered its practical application. In this study, we developed a new PSM strategy to construct an imidazolinium cage salt derivatized from **CC3**. The resultant cage (**OFT‐RCC3^6+^6Br^−^**), containing electron‐rich nitrogen atoms, providing a positively charged confined space and supplying halide ions that can bind I_2_ to form polyhalide anions, made it an ideal molecular container for I_2_ (Scheme 1b). As expected, the iodine capture performance, both the adsorption capacity and the adsorption rate, were drastically improved in comparison with its parent material **CC3**. The PSM strategy had also been corroborated to be a universal method, applicable for constructing charged cages of different sizes. Two other imine cages (**CC1, TC**)^[^ ^17^, ^18^ ^]^ were also modified using the same PSM strategy to investigate their iodine adsorption behaviors and to understand the I_2_ adsorption mechanism in a confined space.

## 2 Results

### 2.1 Synthesis and Characterizations

**Figure 1:**
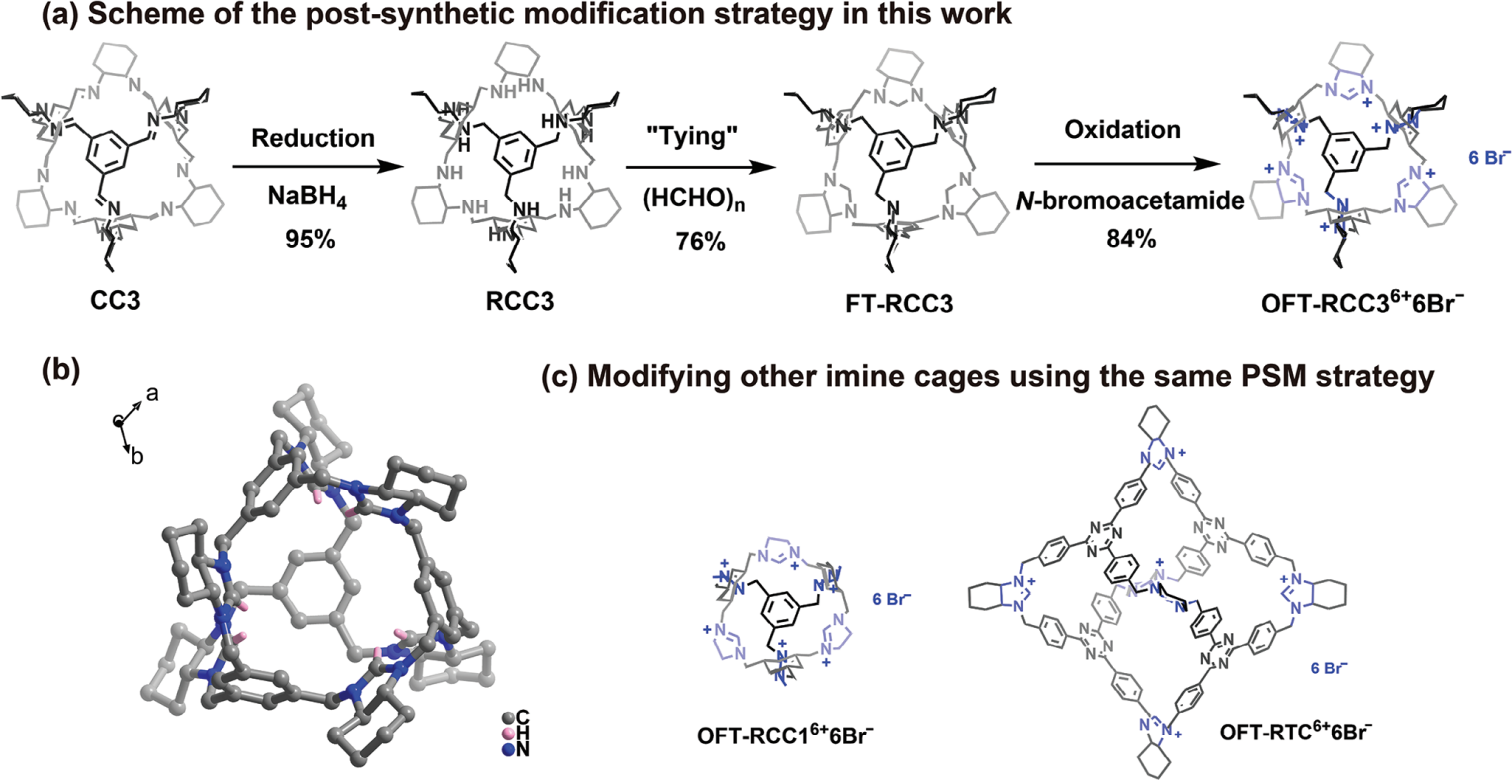
a) Synthesis scheme of **OFT‐RCC3^6+^6Br^−^**. b) Single crystal of **OFT‐RCC3^6+^6Br^−^**. Color code: C, grey; H, pink; N, blue. (anions are omitted for clarity) c) Chemical structures of **OFT‐RCC1^6+^6Br^−^** and **OFT‐RTC^6+^6Br^−^**.

Building on the previously reported “tying strategy”,^[^ ^16b^ ^]^ a nitrogen‐containing five‐membered heterocyclic compound, denoted as **FT‐RCC3**, was successfully synthesized. Subsequent addition of the *N*‐bromoacetamide oxidant led to the formation of an imidazolinium salt structure, with bromide as counterbalancing anions to yield the target product **OFT‐RCC3^6+^6Br^−^** (**Figure** **1**a). Detailed synthetic procedures were presented in Scheme S2 (Supporting Information). The three‐step post‐modification reactions were all highly efficient (the yield for each step exceeds 75%) and operationally simple, with products purified by filtration and washing. The successful synthesis of **OFT‐RCC3^6+^6Br^−^** had been confirmed by a series of characterizations including ^1^H NMR spectroscopy, ^13^C NMR spectroscopy, HSQC NMR spectroscopy, Electro‐spray ionization time‐of‐flight mass spectrometry (ESI‐TOF‐MS), etc. (Figure S10–S13, Supporting Information). The single crystal of **OFT‐RCC3^6+^6Br^−^** was also successfully obtained via diffusion of ethyl ether into a solution of the cage in CH_2_Cl_2_‐MeOH (1:1, v/v) binary solvent mixture. Imidazolinium ring could be clearly observed in Figure 1b, providing sufficient evidence of the successful transformation from the C═N bond in the pristine POC five‐membered heterocycle. Single crystal X‐ray diffraction (SCXRD) study revealed that **OFT‐RCC3^6+^6Br^−^** crystallized in the hexagonal system with a chiral *P*6 space group (Figure S28 and Table S1, Supporting Information). The asymmetric unit contained only 1/3 ionic cage molecules, and each individual cage featured an octahedron structure. Intermolecular C‐H···π interactions were presented in the packing structure of **OFT‐RCC3^6+^6Br^−^**, with C‐H···aromatic plane distances of 2.782 and 2.849 Å (Figure S29, Supporting Information). The majority of bromide ions were distributed disorderly around the cation cage, while C‐H···Br hydrogen bonding interactions with the distance of 2.845 Å could be observed between one of the bromide ions and the cage skeleton (Figure S28, Supporting Information).

Furthermore, the same procedures were carried out on two other cages with different sizes (**CC1, TC**), to verify the universality of this strategy and also to investigate the possibility of further enhancing iodine adsorption performance by increasing the size of the molecular cage cavity. As expected, the modified products (**OFT‐RCC1^6+^6Br^−^**, **OFT‐RTC^6+^6Br^−^**) were obtained successfully as confirmed by solution NMR and MS results (Figure 1c; Schemes S1–S3 and Figures S1–S27, Supporting Information). Also, both the iodine adsorption capacity and adsorption rate had been improved comparing with their parental cage (**CC1**, **TC**) after modification. We failed to get the single crystal structure of these two ionic cages. However, we succeeded in obtaining the single crystal of **FT‐RTC**, as an intermediate of **OFT‐RTC^6+^6Br^−^**, via slow evaporation of the corresponding solution in THF. Single crystal data of **FT‐RTC** suggested the cage skeleton is approximately 31.08 Å in size with a window size of ≈9.14 Å, which is ≈2.3 time of that of the **CC3** derivatives (**FT‐RCC3**) (Figure S30 and Table S2, Supporting Information).

### 2.2 Iodine Vapor Uptake

**Figure 2:**
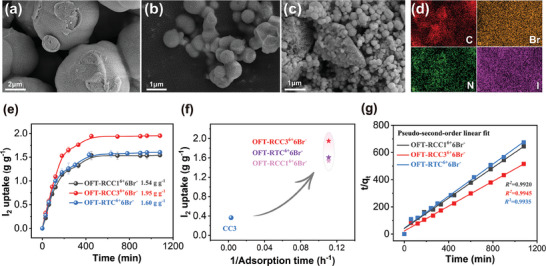
SEM images of the adsorbents after adsorption of iodine, **OFT‐RCC1^6+^6Br^−^** a), **OFT‐RCC3^6+^6Br^−^** b), **OFT‐RTC^6+^6Br^−^** c). d) EDS mapping of **OFT‐RCC1^6+^6Br^−^** after adsorption of iodine. e) Time‐dependent plots of iodine vapor uptake at 75 °C and under ambient pressure. f) Comparison of the iodine vapor uptake performance of **CC3** and three modified cages. g) The kinetic fitting curves by pseudo‐second‐order model for iodine vapor uptake.

The adsorption capacity toward iodine vapor was first evaluated. The iodine vapor uptake experiments were performed by exposing the activated materials to iodine vapor at 75 °C and ambient pressure, which is consistent with the method reported previously.^[^ ^6^, ^8^, ^19^ ^]^ The color of the powder changed from yellow to dark brown accompanied by its mass increase, indicating that the iodine was successfully captured by three synthesized cage adsorbents (Figure S31, Supporting Information). Characterizations, including SEM spectroscopy and EDS mapping (**Figure** **2**a–d; Figures S38 and S39, Supporting Information), further verified the loading of iodine. The uniformly loaded white particles observed after adsorption indicated that the iodine element was homogeneously distributed over the adsorbents **OFT‐RCC1^6+^6Br^−^**, **OFT‐RCC3^6+^6Br^−^**, and **OFT‐RTC^6+^6Br^−^**, as depicted in SEM spectra. The maximum adsorption capacities were determined by weighing the mass of adsorbents before and after adsorption. As shown in Figure 2e, three materials exhibited relatively fast adsorption rates in the initial 200 min, with almost a linear increasing trend. All the adsorption reached equilibrium at ≈9 hours. Confirmed by TGA experiments (Figure S40, Supporting Information), the measured results demonstrated that, the order of maximum adsorption capacities of three adsorbents is **OFT‐RCC3^6+^6Br^−^** (1.95 g g^−1^) > **OFT‐RTC^6+^6Br^−^** (1.60 g g^−1^) > **OFT‐RCC1^6+^6Br^−^** (1.54 g g^−1^), which far exceeded that of **CC3** (0.364 g g^−1^). Furthermore, the adsorption time had been largely reduced from 350 h (**CC3**) to 9 h (**OFT‐RCC3^6+^6Br^−^**) (Figure 2f). On the other hand, the iodine vapor adsorption behaviors of **CC1** and **TC** also had been studied. It was found that the adsorption capacities of **OFT‐RCC1^6+^6Br^−^** and **OFT‐RTC^6+^6Br^−^** were twice the quantity of their parental POCs respectively (Figure S35, Supporting Information). Considering the non‐porosity as checked by N_2_ uptake experiments (Figure S41, Supporting Information), the iodine vapor uptake capacities of three materials were impressive and at a moderate level among the reported discrete cage materials with higher surface areas.^[^ ^5^, ^8^, ^11^, ^20^ ^]^ For further investigating the influence of pore volume on iodine adsorption, their adsorption capacity was compared in molar ratio with the unit of “mol mol^−1^”. The results showed that the I_2_ vapor uptake of **OFT‐RCC3^6+^6Br^−^** (12.91 mol mol^−1^) was lower than that of **OFT‐RTC^6+^6Br^−^** (16.46 mol mol^−1^), which possessed a larger pore volume. Besides, the adsorption capacity of **OFT‐RCC1^6+^6Br^−^** was 8.27 mol mol^−1^, remaining the lowest among the three adsorbents, suggesting the positive correlation between pore volume and adsorption capacity in our system.

Additionally, the adsorption kinetics and adsorption rate had also been studied^[^ ^21^ ^]^ (Tables S3 and S4, Figures S32–S34, Figures S36 and S37, Supporting Information). Compared with fitting the experimental data to pseudo‐first‐order kinetic model, the R^2^ values obtained from pseudo‐second‐order kinetic model are higher (Figure 2g). A higher R^2^ value based on pseudo‐second‐order kinetic model indicated the existence of chemisorption during the adsorption process, which was also supported by the formation of polyhalide ions as suggested by the Raman spectra (Figure 4a‐c). According to the kinetic parameters calculated from a linear fit of pseudo‐second‐order model, the rate constants were as follows: **OFT‐RCC3^6+^6Br^−^** (0.0091 g g^−1^ min^−1^) > **OFT‐RTC^6+^6Br^−^** (0.0079 g g^−1^ min^−1^) > **OFT‐RCC1^6+^6Br^−^** (0.0076 g g^−1^ min^−1^). Furthermore, average adsorption rates (defined as K_80%_)^[^ ^5^, ^8^, ^11^, ^19^, ^21^ ^]^ were determined by the division of the 80% saturated adsorption capacity by the adsorption time for a better appraisal of the materials. It is noteworthy that the K_80%_ values of **OFT‐RCC3^6+^6Br^−^** (0.00723 g g^−1^ min^−1^), **OFT‐RTC^6+^6Br^−^** (0.00495 g g^−1^ min^−1^) and **OFT‐RCC1^6+^6Br^−^** (0.00513 g g^−1^ min^−1^) were all higher than those of reported BPy‐cage (0.004333 g g^−1^ min^−1^)^[^ ^11b^ ^]^ and BTPOC (0.0 03833 g g^−1^ min^−1^),^[^ ^8c^ ^]^ both of which exhibited higher I_2_ adsorption capacity over 3 g g^−1^.

### 2.3 Iodine Adsorption in Solution

**Figure 3:**
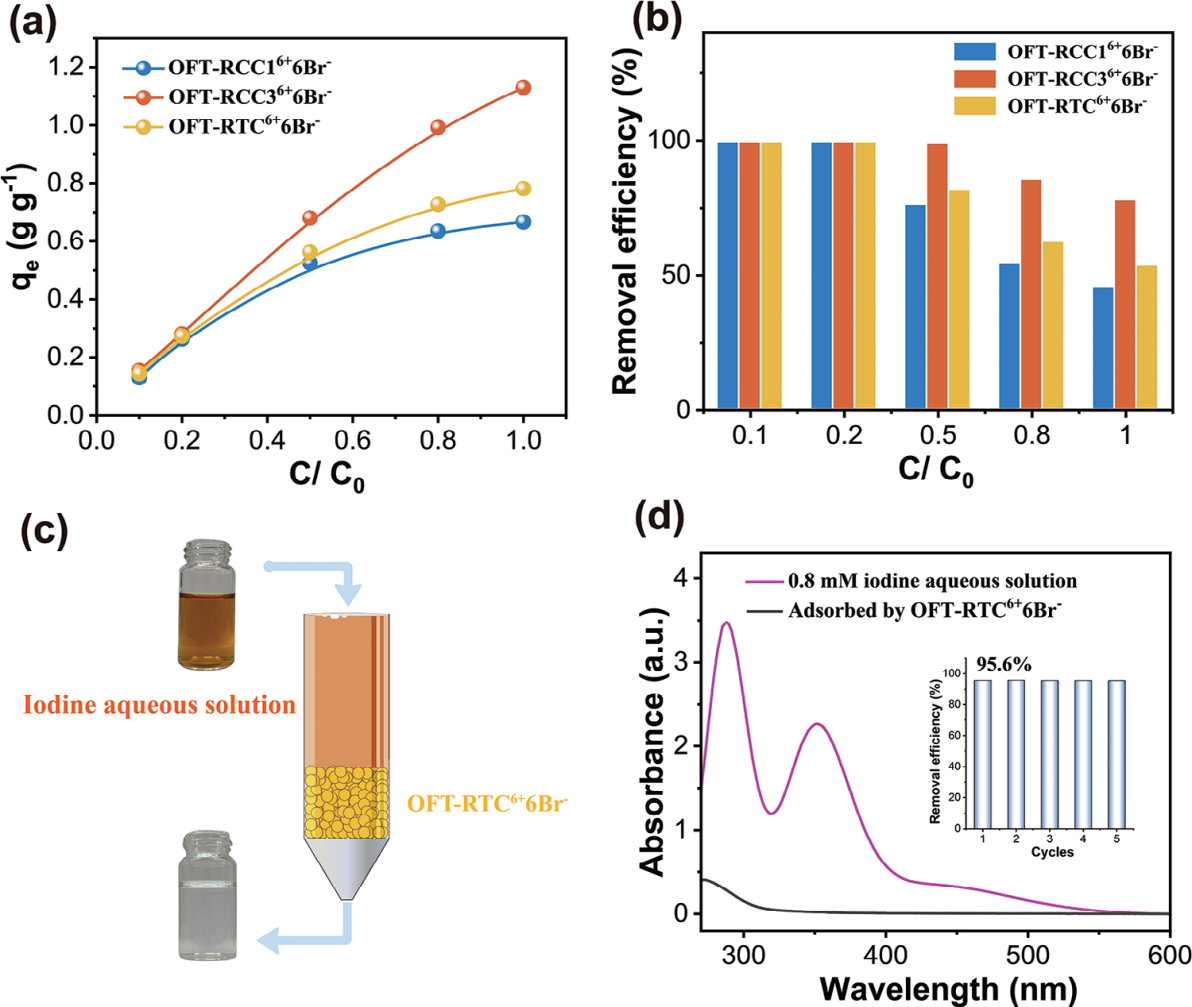
a) Equilibrium adsorption capacity of three adsorbents in iodine *n*‐hexane solution of different concentrations (C_0_ = 1 mg mL^−1^). b) Removal efficiency of three adsorbents in iodine *n*‐hexane solution of different concentrations (C_0_ = 1 mg mL^−1^). c) Column setup used in the aqueous iodine adsorption experiments via flow‐through processes. d) UV/Vis spectra of iodine aqueous solution (0.8 mM) before and after flowing through **OFT‐RTC^6^^+^6Br^−^** adsorbent. The inset shows iodine removal efficiencies after every cycle.

In addition to iodine vapor, the adsorption also occurred in the *n*‐hexane solution of iodine, which could be reflected in the color change from deep purple to colorless after immersing the adsorbents in iodine solution (Figure S60, Supporting Information). 10 mg of adsorbents were added into 15 mL of iodine *n*‐hexane solution with the initial concentration of 0.1, 0.2, 0.5, 0.8 and 1 mg mL^−1^, respectively, and then left for 24 h. During the adsorption process, time‐dependent UV/Vis spectra were carried out to monitor the concentration reduction of iodine based on the intensity changes at 522 nm, thus the equilibrium adsorption capacities and removal efficiency could be calculated (**Figure** **3**a,b). Notably, **OFT‐RCC3^6+^6Br^−^** displayed the highest equilibrium adsorption capacity of 1.13 g g^−1^ in iodine *n*‐hexane solution with a concentration of 1.0 mg mL^−1^. The adsorption rates were also calculated by fitting the data to pseudo‐second‐order model (Figures S47–S53 and Table S5, Supporting Information) with the result of 0.0213 g g^−1^ min^−1^ for **OFT‐RCC1^6+^6Br^−^**, 0.0672 g g^−1^ min^−1^ for **OFT‐RTC^6+^6Br^−^** and 0.2138 g g^−1^ min^−1^ for **OFT‐RCC3^6+^6Br^−^**. **OFT‐RCC3^6+^6Br^−^** showed a much higher I_2_ adsorption rate in organic solvents (≈10 times than that of **OFT‐ RCC1^6+^6Br^−^**, and 3 times than that of **OFT‐RTC^6+^6Br^−^**), which again indicated that **OFT‐RCC3^6+^6Br^−^** had a much higher binding affinity toward I_2_ molecules.

Furthermore, we investigated the use of three materials to capture iodine from the aqueous phase, which represented a real application scenario when water is contaminated by radioactive iodine. Considering the slight water solubility of **OFT‐RCC1^6+^6Br^−^** and **OFT‐RCC3^6+^6Br^−^**, it was challenging to quantify their adsorption capacity using UV–Vis spectroscopy since their adsorption peaks of the aromatic region on cages overlapped with those of I_2_ (Figure S54, Supporting Information). Therefore, the following study focused on **OFT‐RTC^6+^6Br^−^**, which was completely insoluble in water. Upon suspending 5 mg **OFT‐RTC^6+^6Br^−^** into an iodine aqueous solution (0.8 mM, 15 mL), the solution quickly turned transparent, accompanied by a reduced absorbance intensity at 459 nm (Figures S55 and S56, Supporting Information). The adsorption efficiency reached 99.6% within two min, reducing the residue iodine concentration to less than 1 ppm. Its potential for extracting iodine from the aqueous phase promoted us to simulate the decontamination process for practical application scenarios. An iodine aqueous solution (0.8 mM) with a flow rate of 1 mL min^−1^ was used as the mobile phase, and the adsorbent **OFT‐RTC^6+^6Br^−^** filled in a glass column, serving as the stationary phase. This setup created a simple chromatographic column, as shown in Figure 3c. The removal efficiency reached 95.6% after the first cycle (video S1, Supporting Information). After five cycles, the removal efficiency remained nearly unchanged at 95.4%, suggesting good regeneration capacity (Figure 3d). The nearly complete release of iodine could be realized by heating the iodine‐loaded sample at 100 °C under vacuum for 12 h, verified by the EDS analysis (Figure S57, Supporting Information).The results of this laboratory study indicated that the modified POCs hold significant promise for rapidly removing iodine from contaminated aqueous environments via flow‐through processes.

### 2.4 Iodine Adsorption Mechanism Study

**Figure 4:**
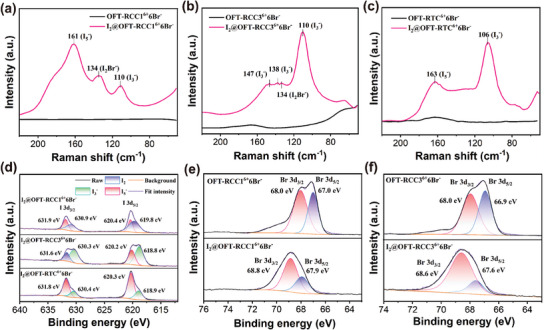
The Raman spectra of adsorbents before and after iodine adsorption, **OFT‐RCC1^6+^6Br^−^** a), **OFT‐RCC3^6+^6Br^−^** b), **OFT‐RTC^6+^6Br^−^** c). d) The XPS I 3d spectra of **OFT‐RCC1^6+^6Br^−^**, OFT‐RCC3^6+^6Br^−,^ and **OFT‐RTC^6+^6Br^−^** after iodine adsorption. The XPS Br 3d spectra of **OFT‐RCC1^6+^6Br^−^** e) and **OFT‐RCC3^6+^6Br^−^** f) before and after iodine adsorption.

To further understand the adsorption process and identify the adsorbed iodine species, we characterized the I_2_‐loaded samples using Raman spectroscopy and X‐ray photoelectron spectroscopy (XPS). Raman spectra results confirmed the formation of polyiodide species and polyhalide ions. As shown in **Figure** **4**a–c, after exposure to iodine, two characteristic peaks at 110 cm^−1^ and 134 cm^−1^ appeared for **I_2_@OFT‐RCC1^6+^6Br^−^** and **I_2_@OFT‐RCC3^6+^6Br^−^**, respectively.^[^ ^5^, ^8^, ^9^ ^]^ The peak at 110 cm^−1^ corresponded to the symmetric stretching of I_3_^−^, while the peak at 134 cm^−1^ was attributed to the stretching of another polyhalide, I_2_Br^−^, implying the interaction between iodine and the bromide ions. Also, the peak of polyhalide could be seen for **I_2_@OFT‐RTC^6+^6Br^−^** (Figure S43, Supporting Information). In addition, the signals emerging at 138 cm^−1^ and 147 cm^−1^ in the spectrum of **I_2_@OFT‐RCC3^6+^6Br^−^** also originated from the asymmetric stretching of I_3_^−^. As for **I_2_@OFT‐RTC^6+^6Br^−^**, the strong band at 106 cm^−1^ reflected the occurrence of I_3_^−^. The characteristic stretching vibration at 163 cm^−1^ confirmed the occurrence of another polyiodide species I_5_^−^, which also could be observed for **I_2_@OFT‐RCC1^6+^6Br^−^** at 161 cm^−1^. A similar phenomenon appeared in the XPS spectra. In the I 3d spectrums, two groups of iodine splitting peaks were seen near 631 and 620 eV after iodine adsorption, which should be ascribed to the I 3d_3/2_ and 3d_5/2_, respectively.^[^ ^5^, ^8^, ^22^ ^]^ Each characteristic peak attributed to the corresponding iodine species is displayed specifically in Figure 4d. The generation of I_2_Br^−^ could be again verified by the recorded Br 3d XPS spectra. It was found that one band centered ≈67.5 eV split into two peaks that were assigned to the Br 3d_3/2_ and 3d_5/2_ orbital levels bands.^[^ ^22b^ ^]^ In the case of **OFT‐RCC1^6+^6Br^−^**, peaks ascribable to Br^−^ exhibited an obvious shift from 67.0 to 67.9 eV and from 68.0 to 68.9 eV after adsorption, providing further evidence for the involvement of bromide ions to adsorb iodine. Similarly, the peaks of Br^−^ in the **OFT‐RCC3^6+^6Br^−^** and **OFT‐RTC^6+^6Br^−^** exhibited an increased binding energy of 1.0 eV after iodine capture (Figure 4e,f; Figure S44, Supporting Information). On the other hand, the shift of 0.2 eV in N 1 s XPS spectra from the imidazolinium ring of adsorbents recorded before and after iodine absorption indicated relatively weaker charge transfer interactions between the nitrogen atom on the imidazolinium ring and iodine species (Figure S45, Supporting Information).

**Figure 5:**
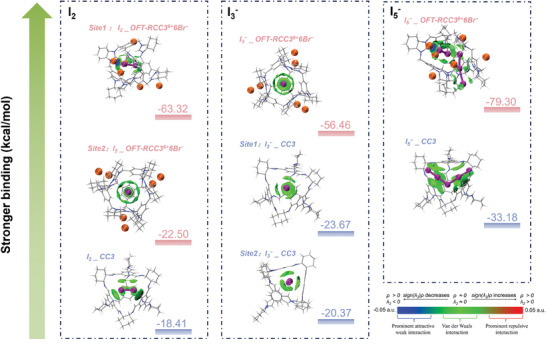
Binding energies and IGMH isosurface of iodine species with **OFT‐RCC3^6+^6Br^−^** (pink) and **CC3** (blue). Carbon, hydrogen, nitrogen, bromine, and iodine atoms are colored grey, white, blue, orange, and purple, respectively. The isosurface values for site1: I_2__**OFT‐RCC3^6+^6Br^−^** and I_5__**OFT‐RCC3^6+^6Br^−^** are set to 0.006 a.u. for clarity, while the rest are set to 0.003 a.u.

Density functional theory (DFT) calculations were also conducted to gain further insights into the adsorption mechanism. (**Figure** **5**). The independent gradient model based on Hirshfeld molecular density partitioning (IGMH) analysis^[^ ^23^ ^]^ was used to visualize non‐covalent interactions. We compared the binding energies of I_2_, I_3_^−^, and I_5_^−^ with **OFT‐RCC3^6+^6Br^−^** and **CC3** at the B3LYP level. The calculated binding energies of iodine species with **OFT‐RCC3^6+^6Br^−^** were ≈2–4 times stronger than **CC3**. Strong I_2_···Br^−^ attractive interactions, indicated by the blue isosurface, were observed in the Site1: I_2__**OFT‐RCC3^6+^6Br^−^**, where I_2_ is placed next to Br^−^, as well as in the I_5_^−^_**OFT‐RCC3^6+^6Br^−^** complex. These complexes showed the highest binding energies of −63.32 and −79.30 kcal/mol, respectively, with a high negative *sign(λ_2_)ρ* values ≈−0.04 (Figure S58, Supporting Information), indicating the significant promoting effect of Br^−^ on the capture of I_2_ and I_5_^−^. During the optimization process, we observed the breaking of I_5_^−^ into I_3_^−^ and I_2_, likely driven by the more energetically favorable I_2_···Br^−^ interaction. The positive charge on the cage skeleton of **OFT‐RCC3^6+^6Br^−^** also promoted the capture of I_2_ and I_3_^−^ compared to the neutral **CC3**. Site2: I_2__**OFT‐RCC3^6+^6Br^−^** (I_2_ is placed inside the cage) and I_3_^−^_**OFT‐RCC3^6+^6Br^−^** showed stronger binding energies than **CC3**. Notably, I_3_^−^_**OFT‐RCC3^6+^6Br^−^** exhibited an increase in binding energy of more than 130%, with non‐covalent interactions observed only between I_3_^−^ and the cationic cage skeleton when the isosurface value was set at 0.003 a.u. A similar phenomenon was also observed in Site2: I_2__**OFT‐RCC3^6+^6Br^−^**. This suggested that the positive charge of the cage is the second factor contributing to iodine adsorption.

### 2.5 The comparison of two Types of Ionic Cages

**Figure 6:**
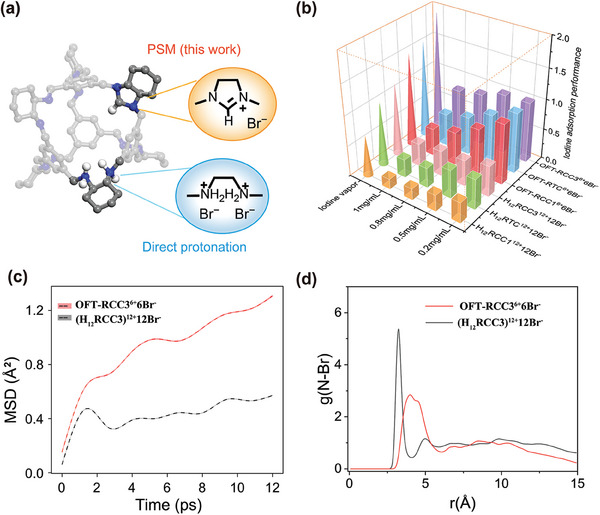
a) The substructures of two types of ionic cages derivatized from **RCC3**. b) The iodine adsorption performance of two types of ionic cages in different circumstances. In the iodine vapor uptake experiment, the adsorption performance is quantified by the mass of iodine adsorbed per gram of adsorbent, expressed in units of g g^−1^. In iodine *n*‐hexane solution of varying concentrations, the adsorption performance is measured by the removal efficiency*100%, after 24 h of contact between 10 mg adsorbent and 15 mL iodine solution. c) MSDs of Iodine and d) N‐Br RDFs in both **OFT‐RCC3^6+^6Br^‐^** and (**H_12_RCC3)^12+^12Br^‐^** calculated using 25 ps AIMD at 348.15 K.

Another way to construct cationic cages with bromide ions as counter anions is directly protonating. By treating the reduced imine cages (**RCC1**, **RCC3**, **RTC**) with hydrobromic acid (HBr), three protonated cationic cages — (**H_12_RCC1)^12+^12Br^−^**, (**H_12_RCC3)^12+^12Br^−^** and (**H_12_RTC)^12+^12Br^−^**, each carrying twelve positive charges, had also been synthesized (Scheme S4–S6, Supporting Information). The difference between the two structures is shown in **Figure** **6**a.

It was found that the iodine adsorption performance of the ionic cages derived from direct protonation was significantly inferior to that of three imidazolinium cage salts prepared using the PSM strategy. In iodine vapor uptake experiments, the three protonated cages exhibited lower adsorption capacities under identical conditions as the imidazolinium cage salts. As illustrated in Figure 6b, among the protonated cages, (**H_12_RCC3)^12+^12Br^−^** achieved the highest iodine vapor uptake value, reaching up to 1.30 g g^−1^, which still did not match any of the imidazolinium cage salts. Similarly, the iodine capture of the protonated cages in solution was also relatively poorer than that of the imidazolinium cages. For example, upon addition of 5 mg (**H_12_RCC3)^12+^ 12Br^−^**, (**H_12_RCC1)^12+^ 12Br^−^** and (**H_12_RTC)^12+^ 12Br^−^** into 3 mL of an iodine *n*‐hexane solution (1 mg mL^−1^), there was only a slight color change after 29 h (Figure S60, Supporting Information). The lower removal efficiency at different concentrations of iodine *n*‐hexane solution suggested that the protonated cages have a lower affinity toward iodine (Figure 6b).

The disparity in iodine adsorption performance between the two types of ionic cages could be attributed to two factors. First, the twelve bromide ions introduced by protonation may be excessively present in the structure, potentially blocking the connected pores of the cage and restricting the transport of iodine molecules within the cavity, which was confirmed by ab initio molecular dynamics (AIMD) calculations. As shown in Figure 6c, I_2_ in **OFT‐RCC3^6+^6Br^−^** had a steeper slope in the mean‐squared displacement (MSD) plot compared to I_2_ in (**H_12_RCC3)^12+^12Br^−^**, indicating that I_2_ diffused more freely in **OFT‐RCC3^6+^6Br^−^**. Second, the first peaks of the N‐Br radial distribution functions (RDFs) (Figure 6d) were located ≈4.1 Å for **OFT‐RCC3^6+^6Br^−^** and 3.2 Å for (**H_12_RCC3)^12+^12Br⁻**, respectively. Compared to the quaternary ammonium structures contained in the protonated cages, the larger steric hindrance of the five‐membered rings in the imidazolinium salt structure may restrict their electrostatic interaction with bromide anions. As a result, the bromide anions in the imidazolinium cage salts had more freedom to interact with iodine molecules and form polyhalide ions, thereby enhancing iodine capture. On the other hand, we also observed that iodine molecules continued interacting with Br^−^ in both cages through the 25 ps AIMD simulations (video S2 and video S3, Supporting Information), which was consistent with the strong binding energy between iodine molecules and Br^−^ as shown in the DFT calculation (Figure 5, site1: I_2__**OFT‐RCC3^6+^6Br^−^**), suggesting the effect of bromide ions in improving iodine adsorption.

## 3 Conclusion

In summary, a three‐step, highly efficient post‐synthetic modification strategy was developed to tune the functionality of porous organic cages. Three modified porous organic cages with different sizes (**OFT‐RCC1^6+^6Br^−^**, **OFT‐RCC3^6+^6Br^−^**, **OFT‐RTC^6+^6Br^−^**) were synthesized using this PSM strategy, which incorporated all desirable structural features for improved iodine adsorption performance. Compared with its parent material **CC3**, the resultant cage (**OFT‐RCC3^6+^6Br^−^**) exhibited a significantly faster adsorption rate and enhanced iodine vapor uptake capacity, which is five times higher than that of **CC3**. Also, **OFT‐RCC1^6+^6Br^−^** and **OFT‐RTC^6+^6Br^−^** showed better iodine adsorption performance than their parental cage (**CC1** and **TC**) respectively. In addition to iodine vapor, excellent iodine adsorption behaviors were also observed in *n*‐hexane and aqueous solutions. The simple chromatographic column experiment, using flowing iodine aqueous solution as a mobile phase, proved the potential of **OFT‐RTC^6+^6Br^−^** for removing iodine rapidly in practical decontamination processes. The approximately unchanged removal efficiency at 95.6% after five cycles, demonstrating its good regeneration capacity. Raman and XPS characterizations, combined with Density functional theory (DFT) and ab initio molecular dynamics (AIMD) calculations, were performed to elucidate the I_2_ adsorption mechanism. The N on the imidazolinium ring, combined with charged skeleton and bromide ions, contributed to the increased iodine adsorption, and the bromide ions may have played the dominant role in enhancing iodine adsorption. With well‐characterized structures, this work provided case studies to understand the structure‐performance relationship for I_2_ adsorption, which will benefit the design of better I_2_ adsorbents in the future.

Furthermore, it was the first time that imine cages had been directly modified into imidazolinium cage salts to our knowledge. This work showcases the effectiveness of the developed PSM strategy in tuning the structure and properties of POCs, which are still underexploited compared with their framework counterparties. The PSM strategy, developed in this work, and those to come, will be a valuable tool to diversify the functions of POCs and promote its applications.

## 4 Experimental Section

### Iodine Vapor Uptake

Time‐dependent iodine vapor uptake experiments were carried out as follows: a glass vial (20 mL) packed with the activated materials (**OFT‐RCC1^6+^6Br^−^**, **OFT‐RCC3^6+^6Br^−^**, **OFT‐RTC^6+^6Br^−^**, 30 mg) was pre‐weighed, and then was transferred to a larger wide‐mouth jar (100 mL) containing a sufficient amount of solid iodine at the bottom. There was no physical contact between the adsorbents and iodine. Next, the jar was sealed and heated at 75 °C at ambient pressure in an oven. At different time intervals of adsorption, the iodine‐loaded materials were cooled down to room temperature and weighed until its weight reached a steady value. The iodine uptake capacities were estimated by the weight gains: *C* = (m_2_‐m_1_)/m_1_*100%, where *C* was the I_2_ uptake capacities, m_1_ and m_2_ were the masses of adsorbents before and after being exposed to I_2_ vapor, respectively. The capacities were determined by at least three parallel experiments. The pseudo‐first‐order and pseudo‐second‐order models were employed to help understand the adsorption kinetics. The linear forms of the two models can be expressed by the following equation (S−1 and S−2):

The pseudo‐first order model (S−1)

(1) $$\mathrm{In}\left(q_{e}-q_{t}\right)=\mathrm{In}\;q_{e}-k_{1}t$$

The pseudo‐second order model (S−2)

(2) $$\frac{t}{q_{t}}=\frac{1}{k_{2}\cdot q_{e}^{2}}+\frac{t}{q_{e}}$$

where *q_e_* (g g^−1^) and *q_t_* (g g^−1^) refer to the adsorption capacity at equilibrium and at time t (min); k_1_ (min^−1^) and k_2_ (g g^−1^ min^−1^) are the adsorption rate constants for the pseudo‐first‐order and pseudo‐second‐order, respectively.

### Iodine Adsorption Experiments in n‐Hexane Solution

Iodine solutions in *n*‐hexane of different initial concentrations (0.1–1.0 mg mL^−1^, 15 mL) were prepared and materials (10 mg) were added to the solutions. After 24 h of adsorption, adsorbents were removed by filtering and the UV‐vis spectra of filtrate were carried out to calculate the equilibrium concentrations of remaining iodine. According to the pre‐made standard calibration curve, the absorbance at 522 nm was chosen to determine the concentration of iodine in the solution. The maximum adsorption capacity (*q_e_*) can be calculated using equation (S‐3) and the efficiency of iodine removal (%) is determined by equation (S‐4).

The maximum adsorption capacity calculation equation (S‐3)

(3) $$q_{e}=(C_{0}-C_{e})\cdot \frac{v}{m}$$

The removal efficiency calculation equation (S‐4)

(4) $$\mathrm{removal}\;\mathrm{efficiency}=\frac{C_{0}-C_{e}}{C_{0}}\;\times 100\%$$

where m (mg) denotes the weight of adsorbents (**OFT‐RCC1^6+^6Br^−^**, **OFT‐RCC3^6+^6Br^−^**, **OFT‐RTC^6+^6Br^−^**) and V (mL) represents the volume of iodine solutions in *n*‐hexane. *C_0_* and *C_e_* (mg mL^−1^) are the initial and equilibrium concentrations of the iodine solutions, respectively.

[CCDC 2368266 and 2368267 contain the supplementary crystallographic data for this paper. These data can be obtained free of charge from The Cambridge Crystallographic Data Centre via http://www.ccdc.cam.ac.uk/data_request/cif.]

## Conflict of Interest

The authors declare no conflict of interest.

## Supporting information



Supporting Information

Supplemental Video 1

Supplemental Video 2

Supplemental Video 3

## Data Availability

The data that support the findings of this study are available from the corresponding author upon reasonable request.
