# Supplementary material for: Post‐Synthetic Modification of Porous Organic Cages for Enhanced Iodine Adsorption Performance
Source: Adv Sci (Weinh). 2024 Oct 14;11(45):2408494. doi: 10.1002/advs.202408494 (PMC11615766; doi:10.1002/advs.202408494)
Supplement: Supplementary file 1 — Supporting Information [file ADVS-11-2408494-s002.docx]

Supporting Information

Post-synthetic Modification of Porous Organic Cages for Enhanced Iodine Adsorption Performance

Qianqian Mao, Siyuan Yang, Jinjin Zhang, Yuanhan Liu and Ming Liu*

Q. Mao, S. Yang, J. Zhang, Y. Liu, M. Liu

Department of Chemistry

Zhejiang University

Xihu, Hangzhou 310058, China
E-mail: mingliu@zju.edu.cn

S. Yang, M. Liu
Hangzhou Global Scientific and Technological Innovation Center (HIC)

Zhejiang University

Xiaoshan, Hangzhou 311215, China

Table of Contents

*1. Materials and methods*

*2. Synthetic procedures and characterization data*

*3. X-ray Experimental Data of* ***OFT-RCC3^6+^6Br*^−^**

*4. Iodine vapor uptake experiments*

*5. Iodine adsorption experiments in n-hexane solution*

*6. Iodine adsorption experiments in aqueous solution*

*7.* *Density functional theory (DFT) and ab initio molecular dynamics (AIMD)* *calculations*

*8. Iodine adsorption performance comparison of two types of ionic cages*

*9. References*

**1. Materials and methods**

All reagents were commercially available, used as supplied without further purification. ^1^H and ^13^C Nuclear Magnetic Resonance (NMR) spectra were collected on a Bruker Advance spectrometer (operating at 600 MHz for ^1^H NMR and 151 MHz for ^13^C NMR). Mass spectra were measured with an Agilent G6545 Q-TOF ESI MS instrument or MALDI-TOF-MS instrument. UV−vis spectra were measured using a UV-3600i Plus UV-Vis-NIR spectrometer at room temperature. Raman spectra were recorded on a Horiba LabRAM HR Evolution spectrometer equipped with a 532 nm laser. X-ray photoelectron spectroscopy (XPS) measurements were carried out using a Thermo Scientific K-Alpha instrument. Scanning electron microscopy (SEM) and energy-dispersive spectroscopy (EDS) experiments were performed on a Scios2 Hivac scanning electron microscope. Thermogravimetric analysis (TGA) were carried out under nitrogen atmosphere on a Discovery TGA 550 thermogravimeter with a heating rate of 5°C/min. The surface areas were measured by N_2_ adsorption and desorption at 77 K using a Micromeritics ASAP 2020 volumetric adsorption analyser, and samples were degassed at offline at 80 ℃ for 12 h under vacuum. Single crystal X-ray diffraction data were collected on a Bruker D8 Venture diffractometer with a CCD area detector (Cu-Kα radiation, λ = 1.54178 Å) at 150(2) K. The Bruker APEX III program^[1]^ was used to determine the unit cell parameters and for data collection. The data were integrated and corrected for Lorentz and polarization effects using SAINT. The structures were solved by direct methods and refined by full-matrix least squares method on F2 using the SHELXTL^[2]^crystallographic software package and Olex2^[3]^. All non-hydrogen atoms were refined with anisotropic thermal parameters, whereas all hydrogen atoms were placed at calculated positions and refined using a riding model. Crystallographic data for the structures reported in this paper have been deposited with the Cambridge Crystallographic Data Centre^[4]^. CCDC 2368266-2368267 contain the supplementary crystallographic data for this paper. These data can be obtained free of charge from The Cambridge Crystallographic Data Centre via www.ccdc.cam.ac.uk/structures. This report and the CIF file were generated using FinalCif ^[5]^. Details on the crystallographic data, selected bond lengths and bond angles are listed in Table S1 to Table S2.

**2. Synthetic procedures and characterization data**

**2.1 Synthetic procedures**

***Scheme S1.*** Synthesis scheme of **OFT-RCC1^6+^6Br^-^*.***

***Scheme S2.*** Synthesis scheme of **OFT-RCC3^6+^6Br^-^*.***

***Scheme S3.*** Synthesis scheme of **OFT-RTC^6+^6Br^-^*.***

***Scheme S4.*** Synthesis scheme of **(H_12_RCC1)^12+^12Br^-^*.***

***Scheme S5.*** Synthesis scheme of **(H_12_RCC3)^12+^12Br^-^*.***

***Scheme S6.*** Synthesis scheme **(H_12_RTC)^12+^12Br^-^*.***

**2.2 Synthesis details**

**Synthesis of RCC1. RCC1** was synthesized according to literature procedure^[6]^. ^1^H NMR (600 MHz, CDCl_3_, 298 K) *δ* (ppm): 7.09 (s, 12H, -ArH), 3.68 (s, 24H, -ArCH_2_), 2.65 (s, 24H, -NCH_2_).

**
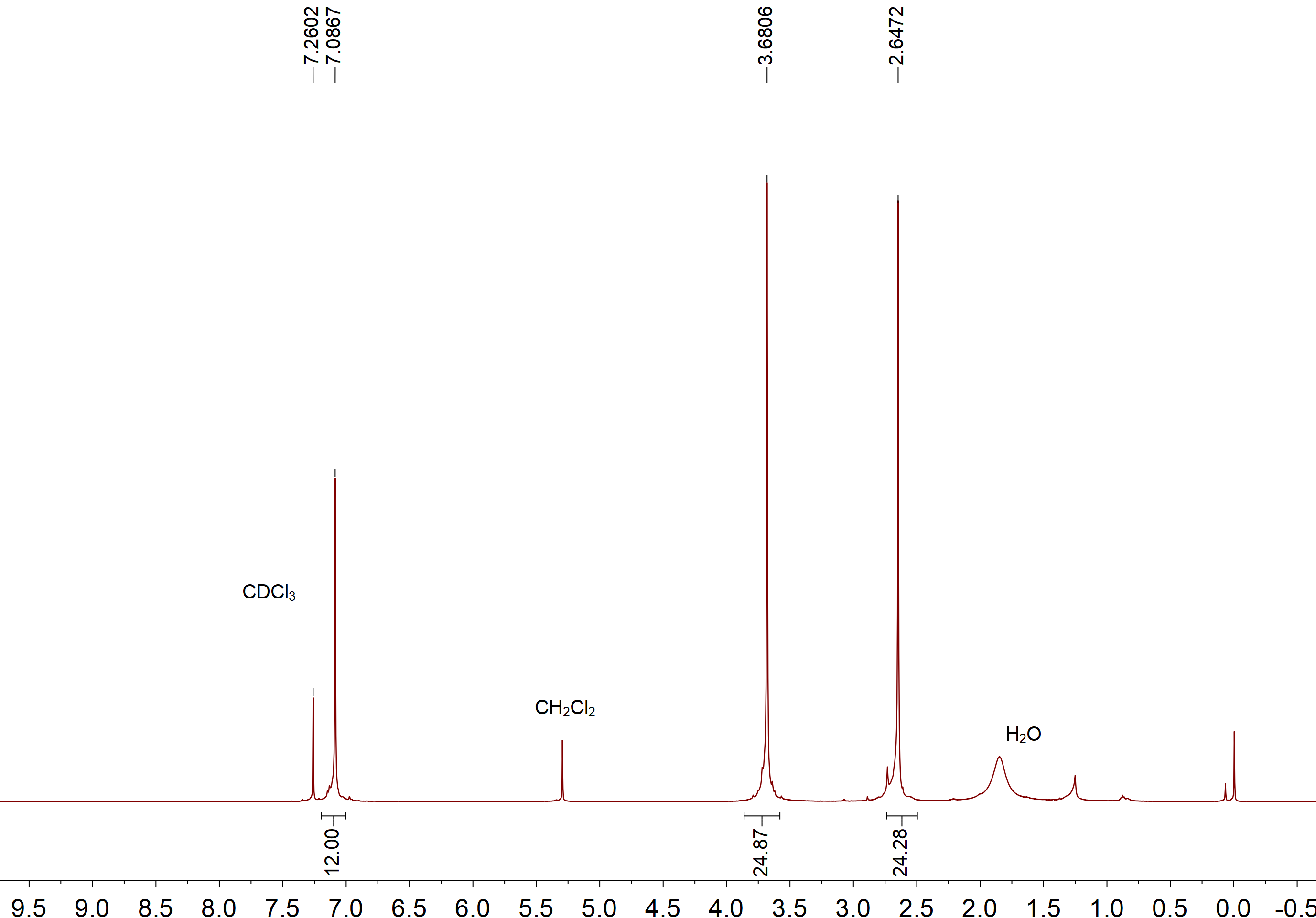
**

***Figure S1*** ^1^H NMR spectrum (600 MHz, CDCl_3_, 298 K) of **RCC1**

**Synthesis of FT-RCC1.** Paraformaldehyde (147.0 mg, 20 eq.) dissolved in CH_3_OH (20 mL) was stirred at 70 °C. To this clear solution was added **RCC1** (200 mg, 0.245 mmol) dissolved in CH_3_OH (20 mL). The reaction was stirred for a further 30 min at 70 °C. The reaction was cooled to room temperature and the solvent was then removed under vacuum. Pure **FT-RCC1** crystalized from the solution of MeOH. Yield: 152.4 mg, 70 %. ^1^H NMR (600 MHz, CDCl_3_, 298 K) δ (ppm): 7.09 (s, 12H, -ArH), 3.66 (s, 24H, -ArCH_2_), 3.13 (s, 12H, -NCH_2_N), 2.93 (s, 24H, -NCH_2_). ^13^C NMR (151 MHz, CDCl_3_, 298 K) δ (ppm): 53.4, 59.6, 76.2, 125.5, 139.5. MS (ESI-HRMS): m/z [M+H]^+^ calcd for [C_54_H_73_N_12_] ^+^: 889.6076, found: 889.6084.


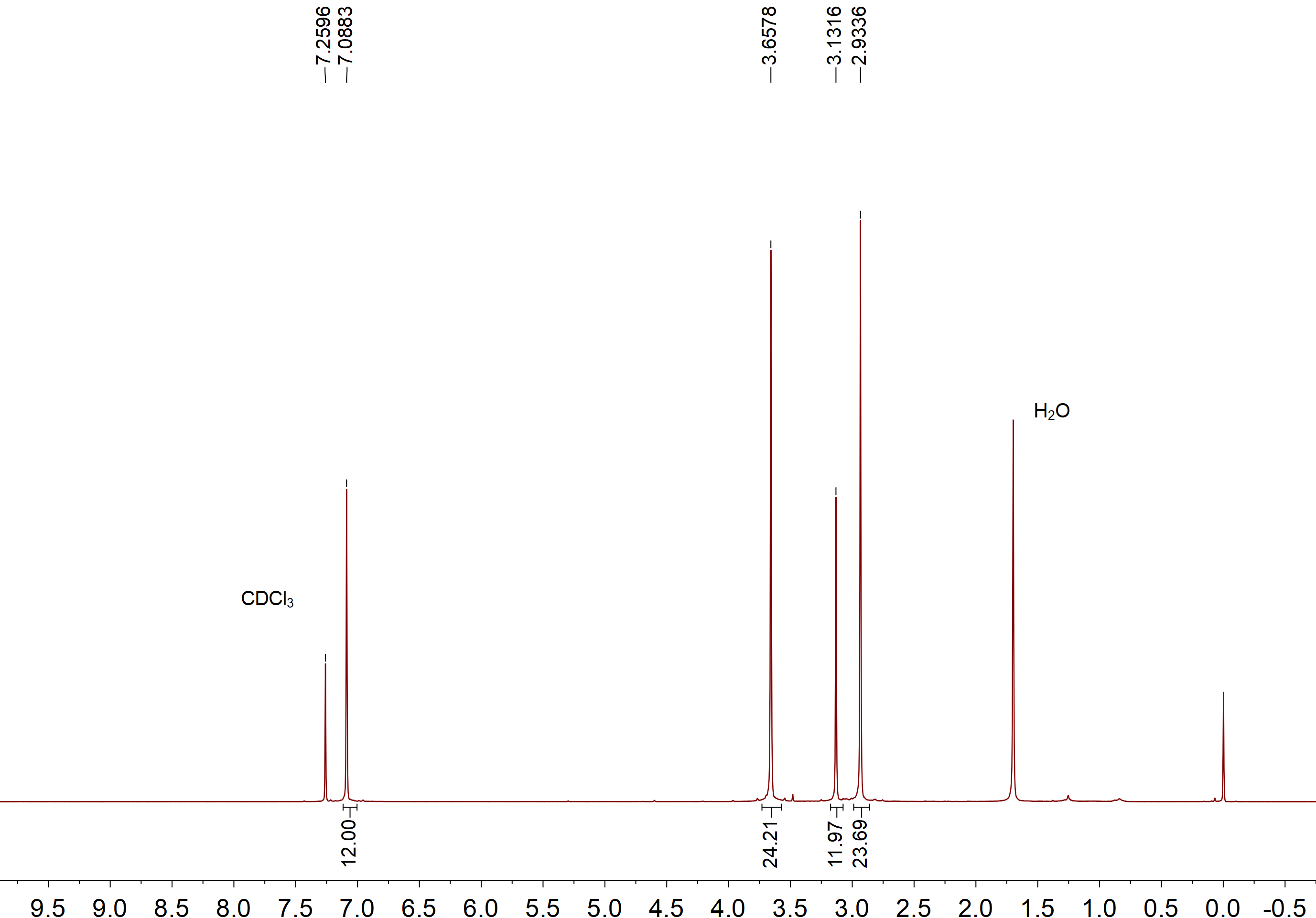


***Figure S2*** ^1^H NMR spectrum (600 MHz, CDCl_3_, 298 K) of **FT-RCC1.**


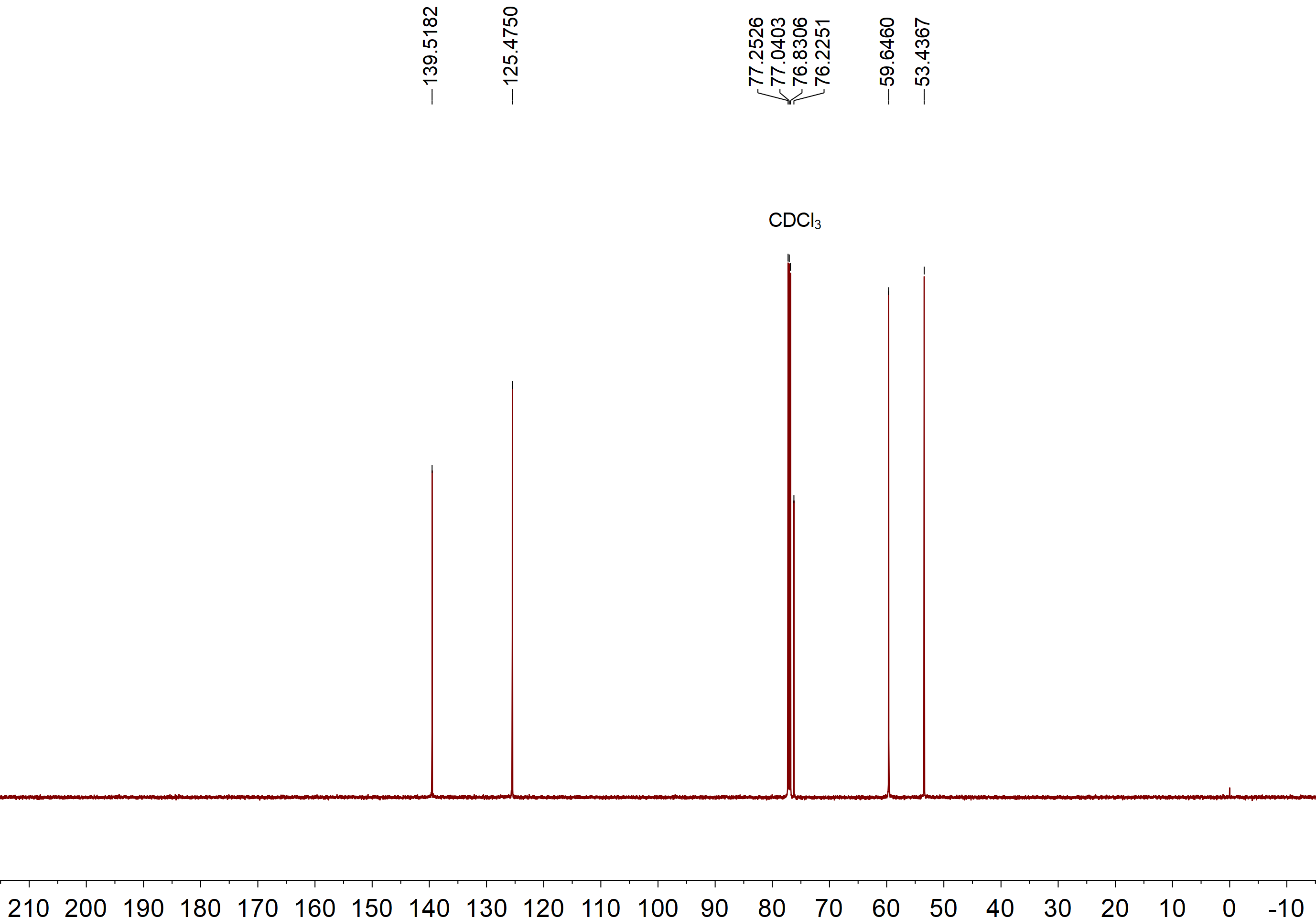


***Figure S3*** ^13^C NMR spectrum (151 MHz, CDCl_3_, 298 K) of **FT-RCC1.**


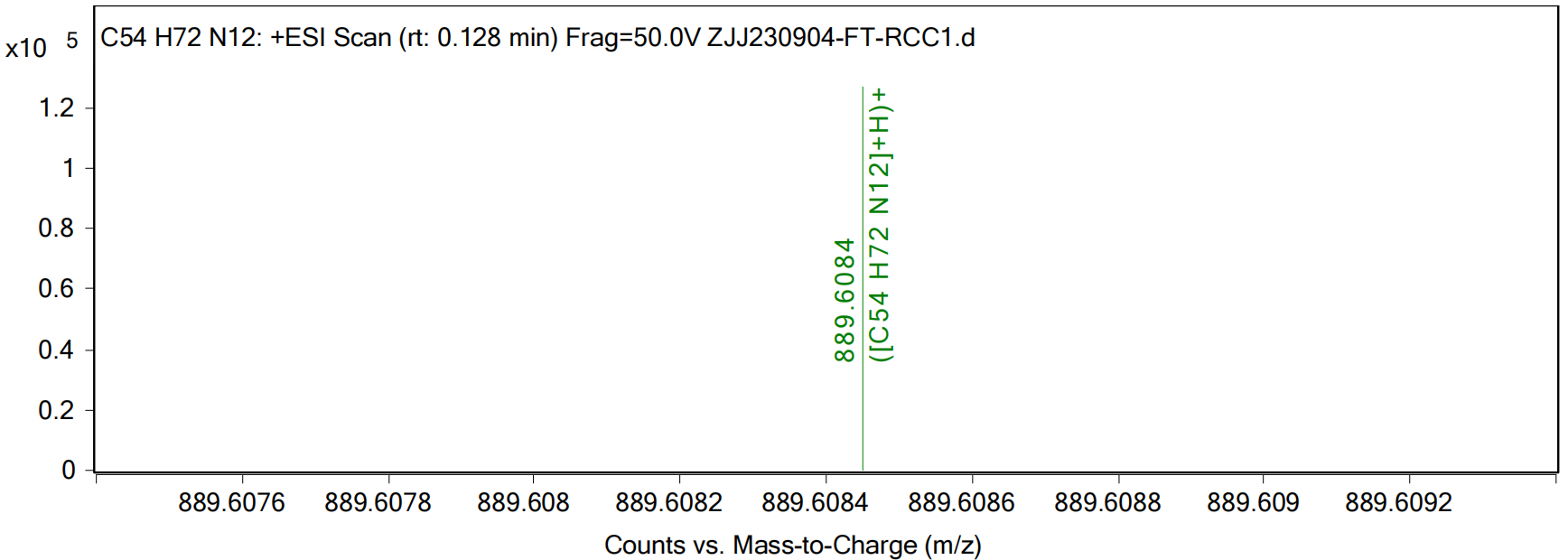


***Figure S4*** HR-MS spectrum of **FT-RCC1.**

**Synthesis of** **OFT-RCC1^6+^6Br^−^.** **FT-RCC1** (150.0 mg, 0.169 mmol) was immersed in 1,2-dimethoxyethane (20 mL). *N*-Bromacetamide (210.0 mg, 1.521 mmol) was added in two portions (0.76 mmol each) with an interval of 15 min. After addition of the second portion, the reaction mixture was stirred for an additional hour. The product precipitated and was isolated by filtration. **OFT-RCC1^6+^6Br^−^** was obtained after MeOH washing. Yield: 100.5 mg, 45%. ^1^H NMR (600 MHz, DMSO-*d*_6_, 298 K) δ (ppm): 8.76 (s, 6H, -N=CH), 7.55 (s, 12H, -ArH), 4.65 (s, 24H, -Ar-CH_2_), 4.10 (s, 24H, -NCH_2_). ^13^C NMR (151 MHz, DMSO-*d*_6_, 298 K) δ (ppm): 50.0, 50.1, 129.5, 136.0, 158.6. MS (ESI-HRMS): m/z [M-Br]^+^ calcd for [C_54_H_66_Br_5_N_12_]^+^: 1281.1404, found: 1281.14087; [M-2Br]^2+^ calcd for [C_54_H_66_Br_4_N_12_]^2+^: 601.1108, found: 601.11075; [M-3Br]^3+^ calcd for [C_54_H_66_Br_3_N_12_]^3+^: 373.7682, found: 373.76822; [M-4Br]^4+^ calcd for [C_54_H_66_Br_2_N_12_]^4+^: 260.5964, found: 206.59653.

**
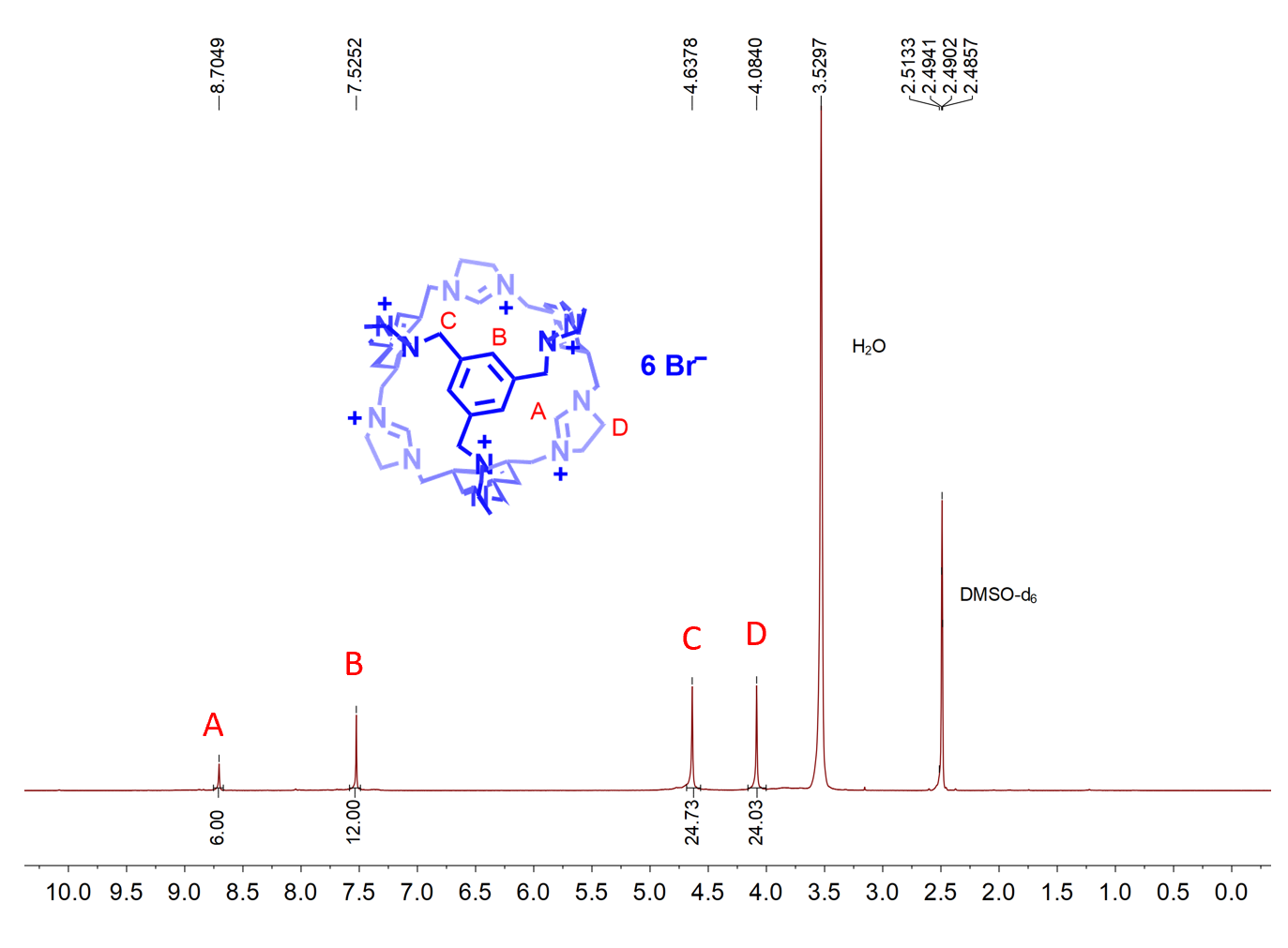
**

***Figure S5*** ^1^H NMR spectrum (600 MHz, DMSO-d_6_, 298 K) of **OFT-RCC1^6+^Br^−^.**


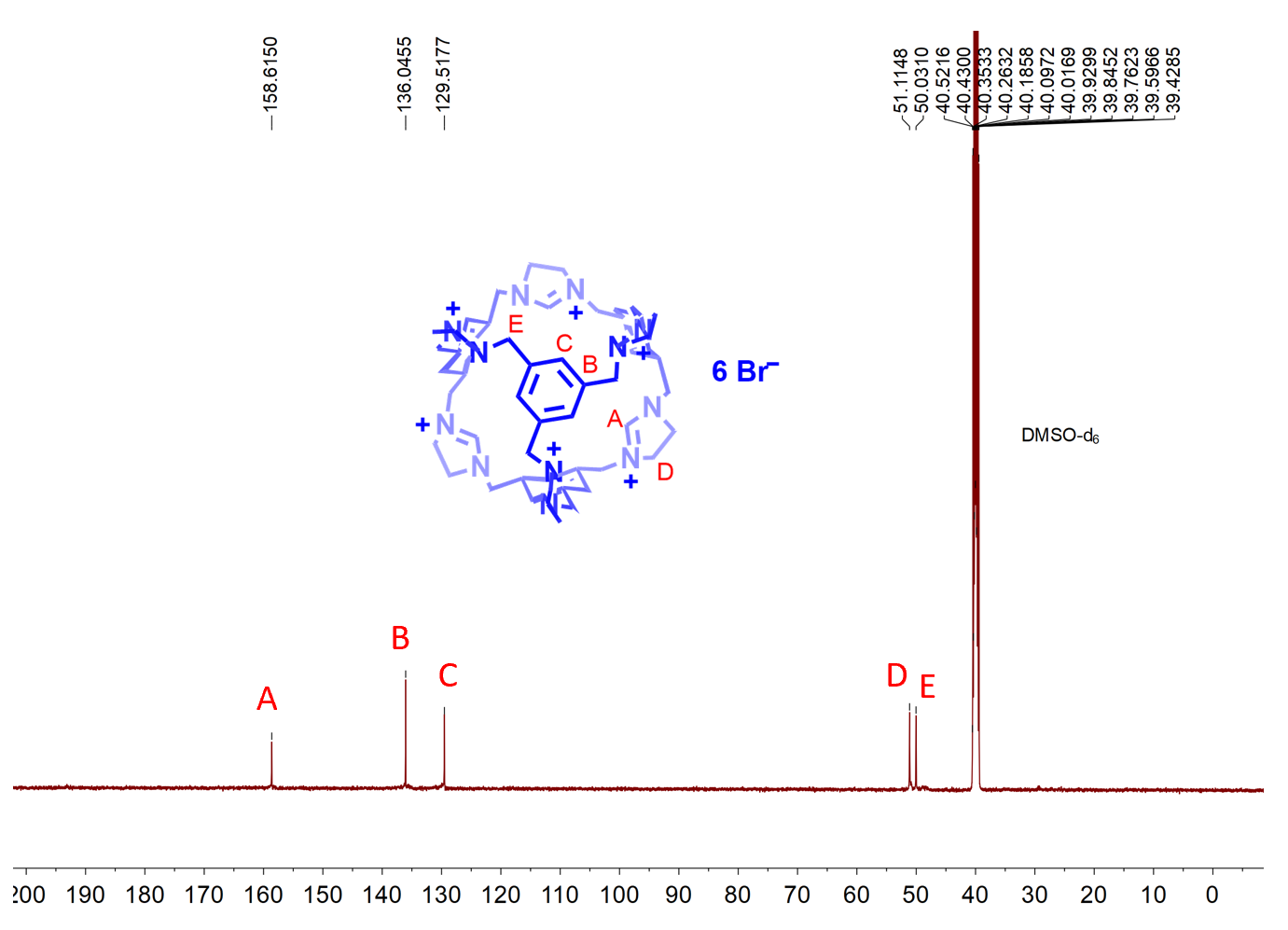


***Figure S6*** ^13^C NMR spectrum (151 MHz, DMSO-d_6_, 298 K) of **OFT-RCC1^6+^6Br^−^.**

***Figure S7*** HR-MS spectrum of **OFT-RCC1^6+^6Br^−^.**

**Synthesis of RCC3. RCC3** was synthesized according to literature procedure^[7]^. ^1^H NMR (600 MHz, CDCl_3_, 298 K) *δ* (ppm): 7.12(s, 12H, -ArH), 3.82 (d, 12H, -ArCH_2_), 3.57 (d, 12H, -ArCH_2_), 2.20 (m, 12H, CH on cyclohexane), 0.95-2.01(m, 48H, CH_2_ on cyclohexane).


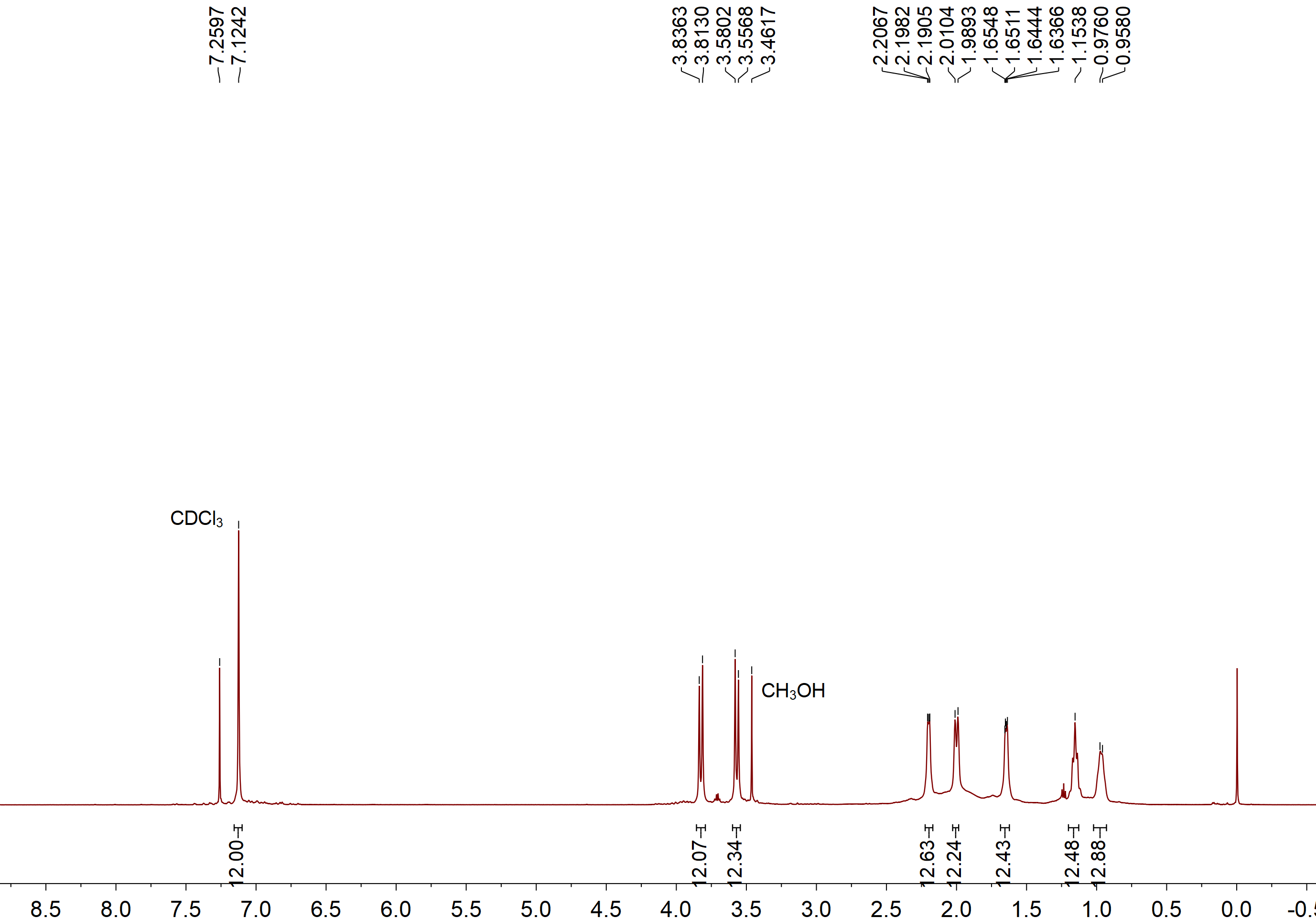


***Figure S8*** ^1^H NMR spectrum (600 MHz, CDCl_3_, 298 K) of **RCC3.**

**Synthesis of FT-RCC3. FT-RCC3** was synthesized according to literature procedure^[7]^. ^1^H NMR (600 MHz, CDCl_3_, 298 K) *δ* (ppm): 7.11(s, 12H, -ArH), 4.01 (d, 12H, -ArCH_2_), 3.23 (s, 12H, -NCH_2_N), 3.19 (s, 12H, -ArCH_2_), 2.28 (d, 12H, CH on cyclohexane), 1.96(d, 12H, CH_2_ on cyclohexane), 1.81(d, 12H, CH_2_ on cyclohexane), 1.28 (m, 24H, CH_2_ on cyclohexane).


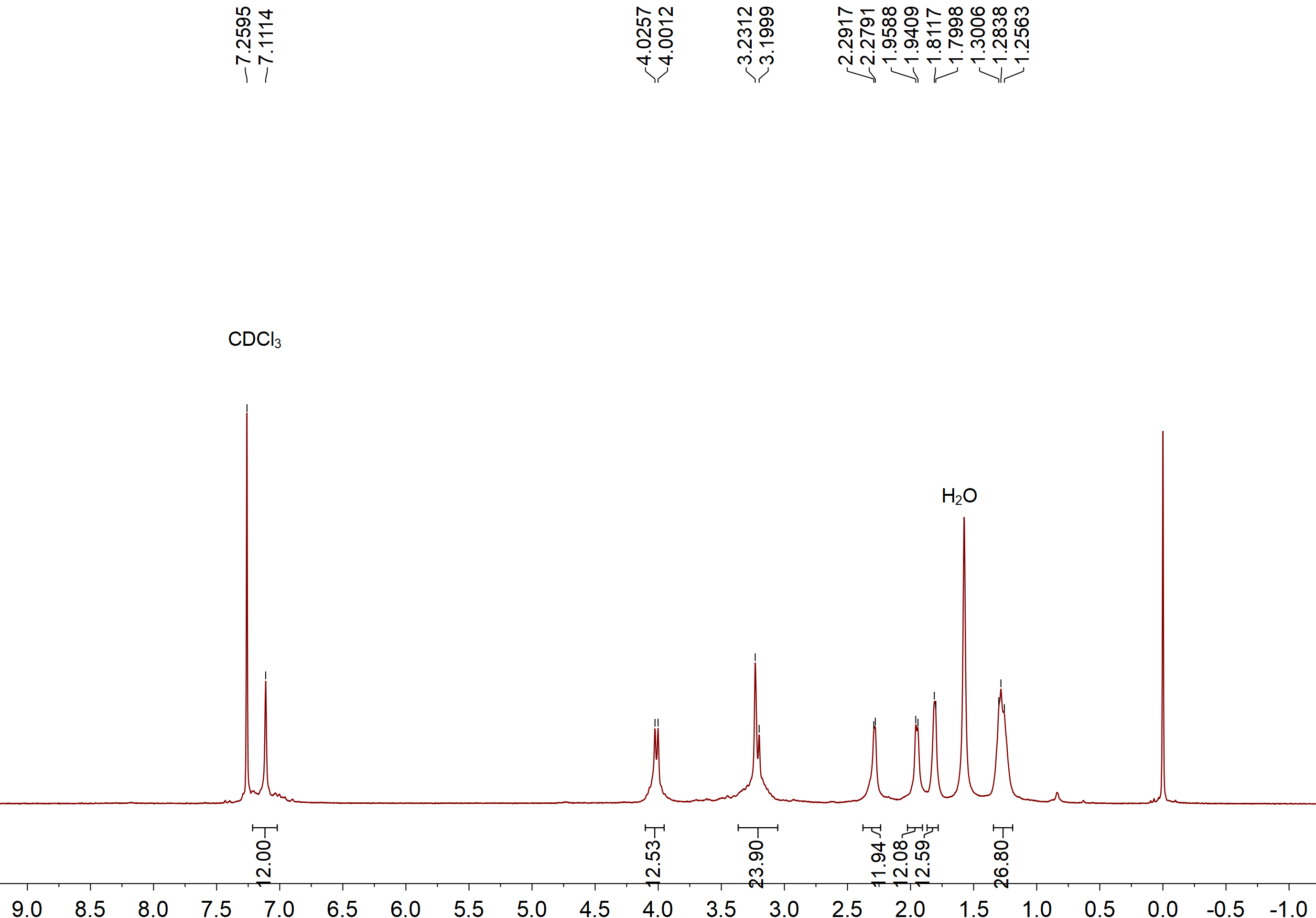


***Figure S9*** ^1^H NMR spectrum (600 MHz, CDCl_3_, 298 K) of **FT-RCC3.**

**Synthesis of OFT-RCC3^6+^·6Br^-^.** **FT-RCC3** (120 mg, 0.099 mmol) was immersed in 1,2-dimethoxyethane (10 mL). *N*-Bromacetamide (106 mg, 0.77 mmol, 7.8 eq.) was added in two portions (0.385 mmol each) with an interval of 15 min. After addition of the second portion, the reaction mixture was stirred for 3 hours. The product precipitated and was isolated by filtration. After washing by Et_2_O, **OFT-RCC3^6+^6Br^−^** was purified and obtained. Yield: 140 mg, 84 %. ^1^H NMR (600 MHz, CDCl_3_, 298 K) δ (ppm): 8.23 (s, 6H, -N=CH), 7.52 (s, 12H, -ArH), 4.86 (d, 12H, -ArCH_2_), 4.67 (d, 12H, -ArCH_2_), 3.95 (d, 12H, CH on cyclohexane), 2.25 (d, 12H, CH_2_ on cyclohexane), 1.97 (d, 12H, CH_2_ on cyclohexane), 1.78 (m, 12H, CH_2_ on cyclohexane), 1.46 (m, 12H, CH_2_ on cyclohexane).^13^C NMR (151MHz, CDCl_3_, 298 K) δ (ppm): 162.08, 135.47,127.94, 69.16, 49.47, 27.14, 23.83. MS (ESI-HRMS): m/z [M-2Br]^2+^ calcd for [C_78_H_102_Br_4_N_12_]^2+^: 763.2516, found: 763.62860; [M-3Br]^3+^ calcd for [C_78_H_102_Br_3_N_12_]^3+^: 482.5281 found: 482.77924; [M-5Br]^5+^ calcd for [C_78_H_102_BrN_12_]^5+^: 257.5497, found: 257.81464.


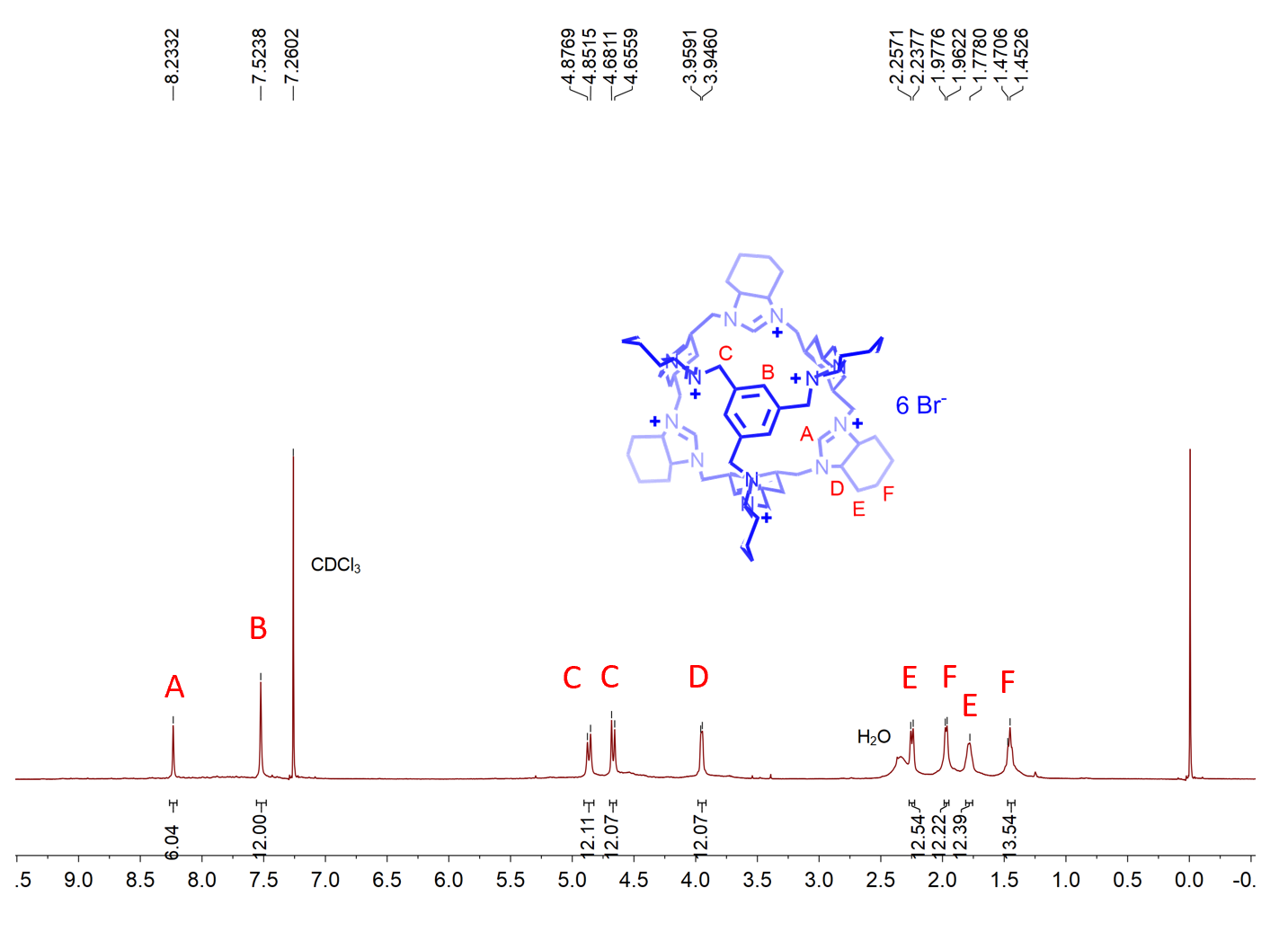


***Figure S10*** ^1^H NMR spectrum (600 MHz, CDCl_3_, 298 K) of **OFT-RCC3^6+^6Br^−^.**


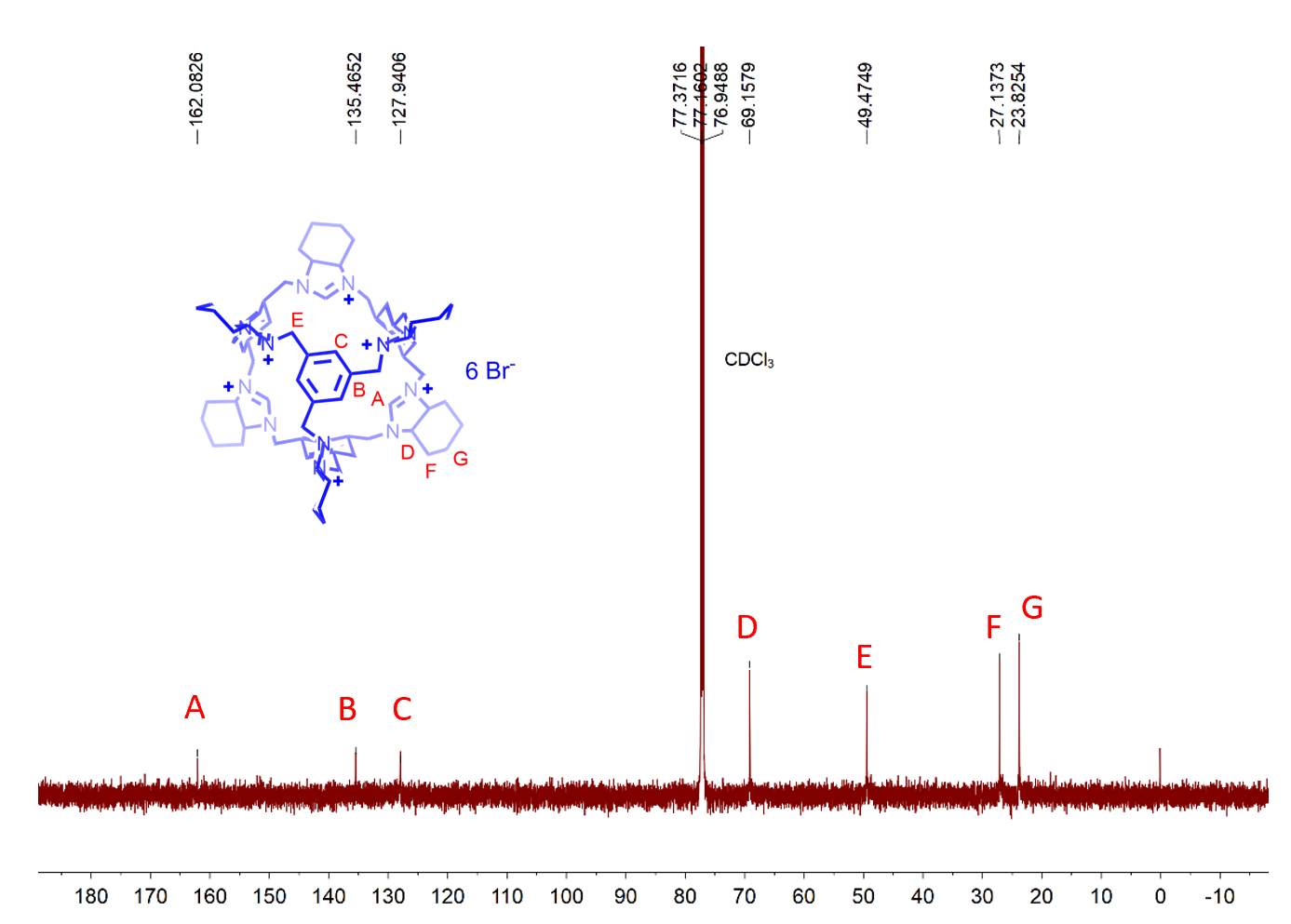


***Figure S11*** ^13^C NMR spectrum (151 MHz, CDCl_3_, 298 K) of **OFT-RCC3^6+^6Br^-^.**

**
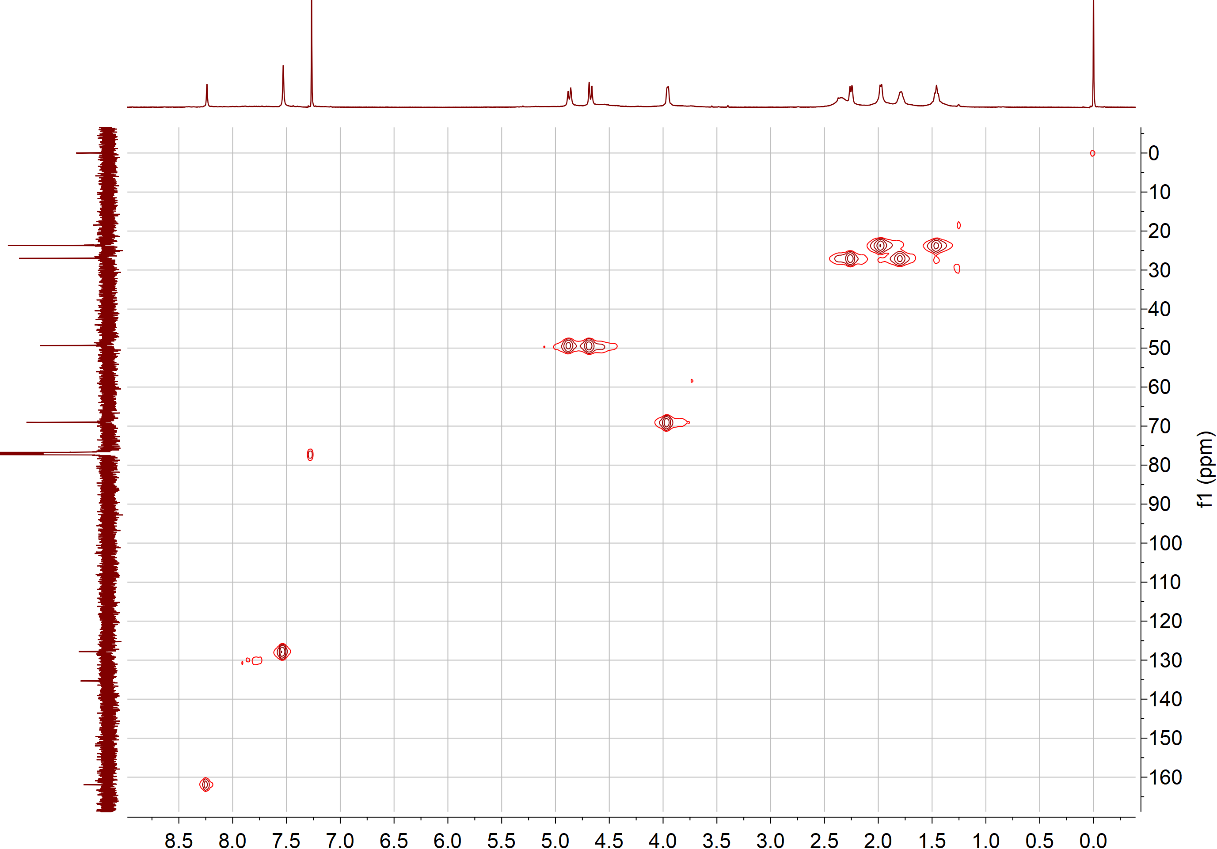
**

***Figure S12*** HSQC NMR spectrum (CDCl_3_, 298 K) of **OFT-RCC3^6+^6Br^-^.**


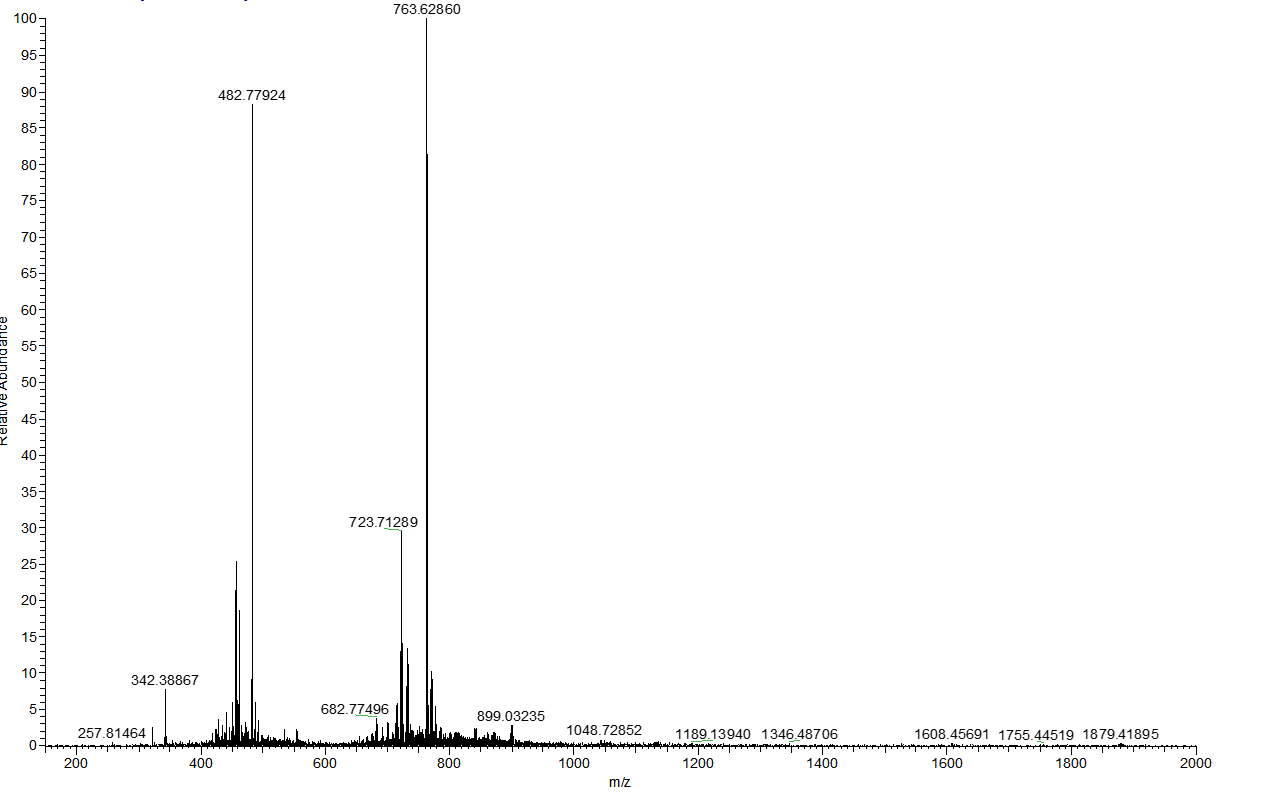


***Figure S13*** HR-MS spectrum of **OFT-RCC3^6+^6Br^−^.**

**Synthesis of TC. TC** was synthesized according to literature procedure^[8]^. ^1^H NMR (600 MHz, CDCl_3_, 298 K) *δ* (ppm): 8.59 (d, 24H, -ArH), 8.31 (s, 12H, -C=NH), 7.77 (d, 24H, -ArH), 3.52 (s, 12H, CH on cyclohexane), 1.91-1.65 (m, 48H, CH_2_ on cyclohexane). HR-MALDI-TOF-MS(DCTB): m/z [M+H]^+^ calcd for [C_132_H_121_N_24_]^+^: 2042.0206 , found : 2042.5615.


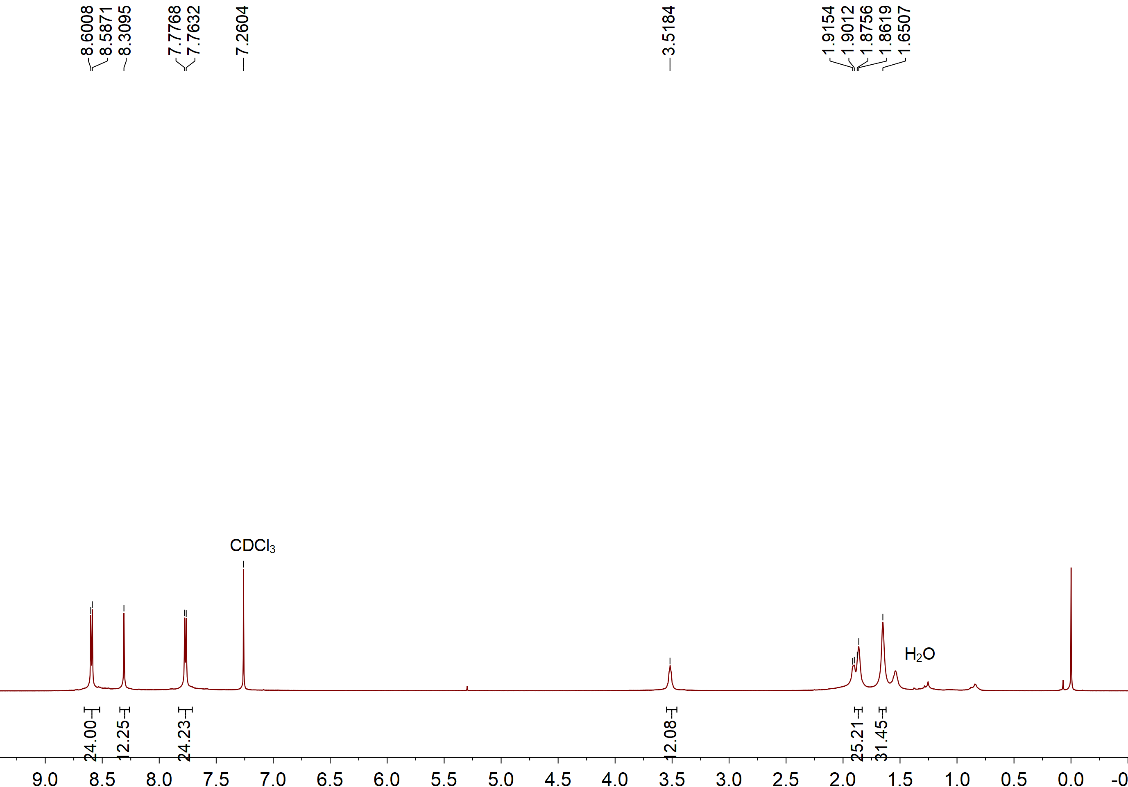


***Figure S14*** ^1^H NMR spectrum (600 MHz, CDCl_3_, 298 K) of **TC.**

**
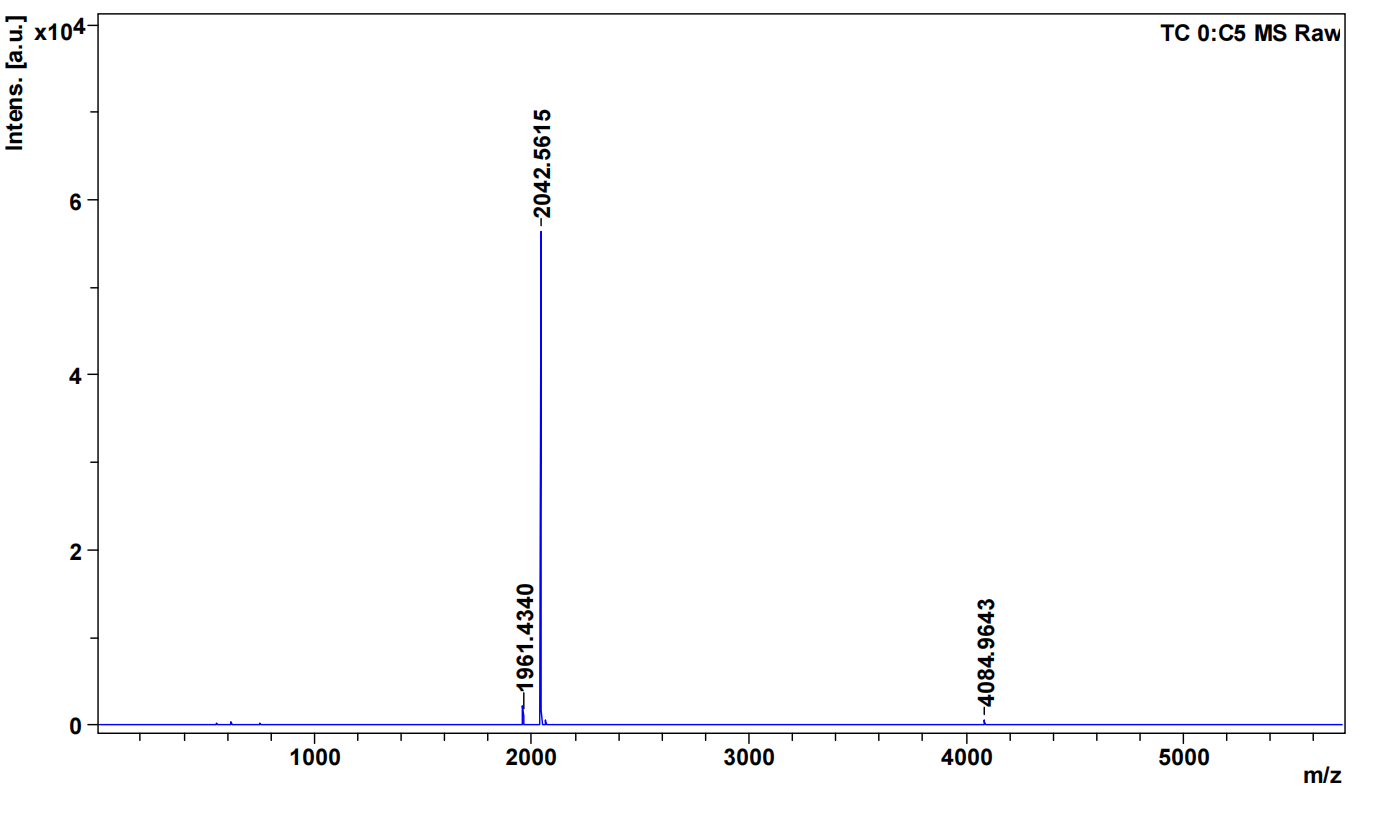
**

***Figure S15*** The HR-MALDI-TOF-MS of **TC**.

**Synthesis of RTC.** In a 250 mL round bottom flask, **TC** (200 mg, 0.098mmol) was dissolved in CHCl_3_-MeOH (1:1, v/v) binary solvent mixture by stirring. NaBH_4_ (88.9 mg, 2.35 mmol, 24 eq.) was added at room temperature when this solution became clear and stirred for a further 48 hours. Water (2 mL) was then added, and the reaction stirred for a further 12 hours. The solvent was then removed under vacuum. The resulting white solid was extracted with CHCl_3_ (2 × 50 mL) and then the combined organic phase was washed by water (2 × 100 mL). The CHCl_3_ phase was dried using anhydrous MgSO_4_, before being dried at 60 °C under vacuum overnight. RTC (Yield: 154 mg, 76 %) was obtained as a white solid. ^1^H NMR (600 MHz, CDCl_3_, 298 K) *δ* (ppm): 8.66-8.29 (m, 24H, -ArH), 7.53-7.20 (m, 24H, -ArH), 4.05-3.44 (m, 24H, -ArCH_2_), 2.35 (s, 12H, CH on cyclohexane), 2.13-2.10,1.30-1.17(m, 48H, CH_2_ on cyclohexane). ^13^C NMR (151MHz, CDCl_3_, 298 K) δ (ppm):171.02, 145.44, 134.65, 128.71, 127.91, 50.70, 31.49, 29.64, 25.20. HR-MALDI-TOF-MS (DHB): m/z [M+H]^+^ calcd for [C_132_H_145_N_24_]^+^: 2067.2118 , found : 2067.065.


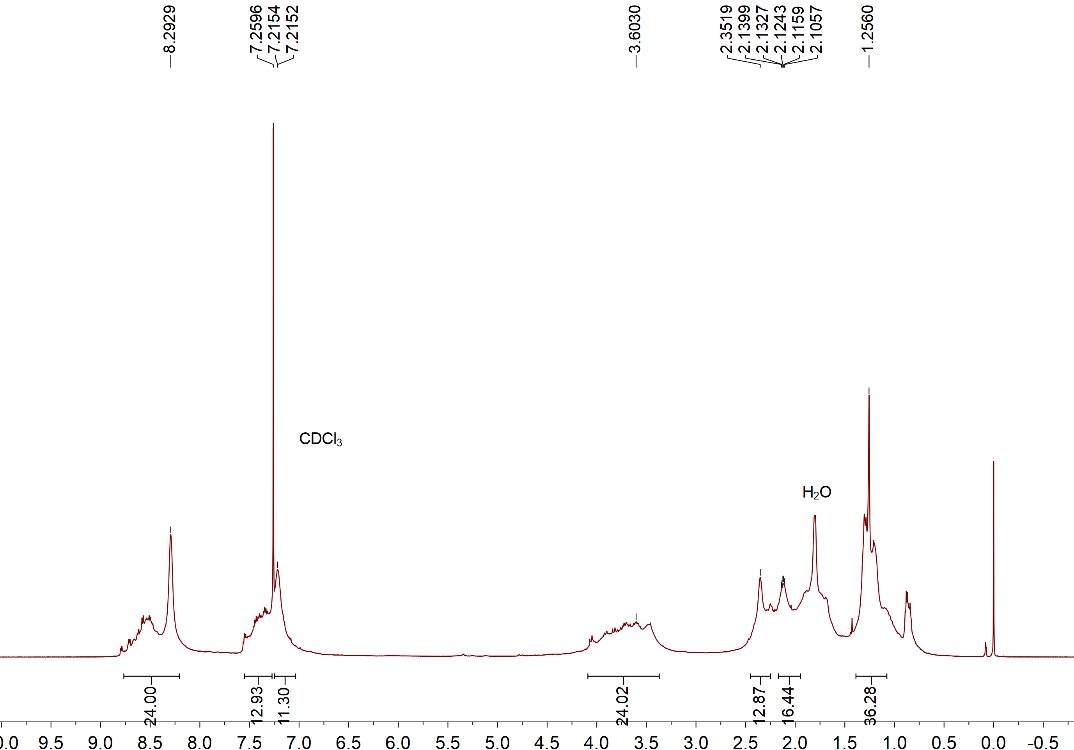


***Figure S16*** ^1^H NMR spectrum (600 MHz, CDCl_3_, 298 K) of **RTC.**


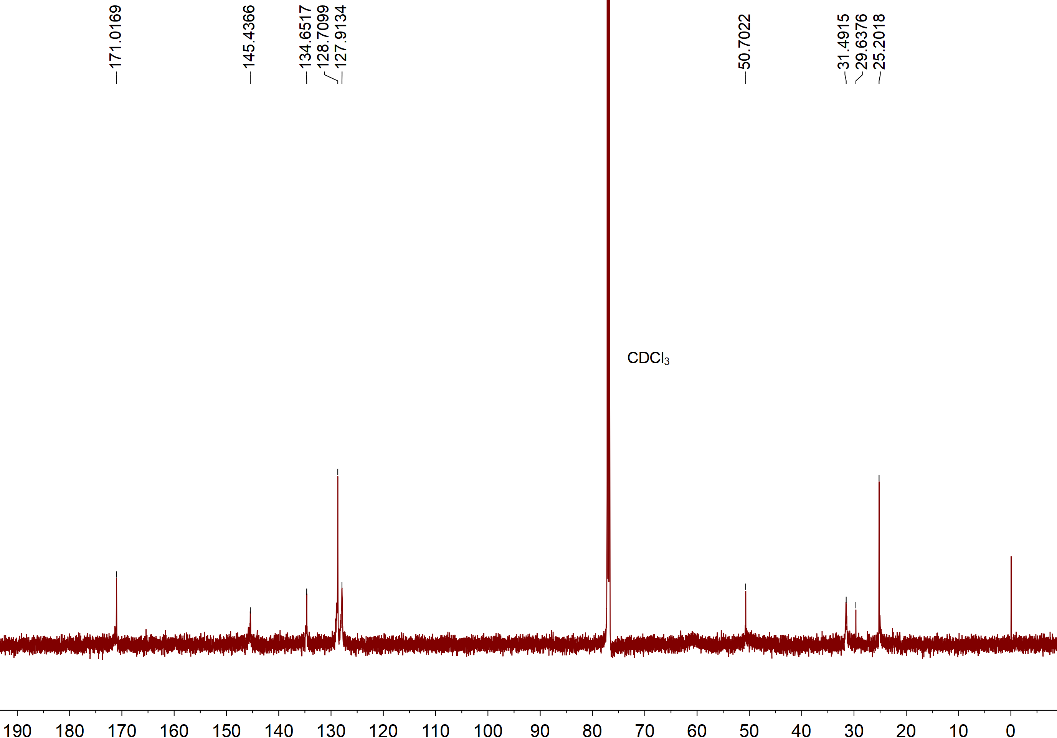


***Figure S17*** ^13^C NMR spectrum (151 MHz, CDCl_3_, 298 K) of **RTC.**


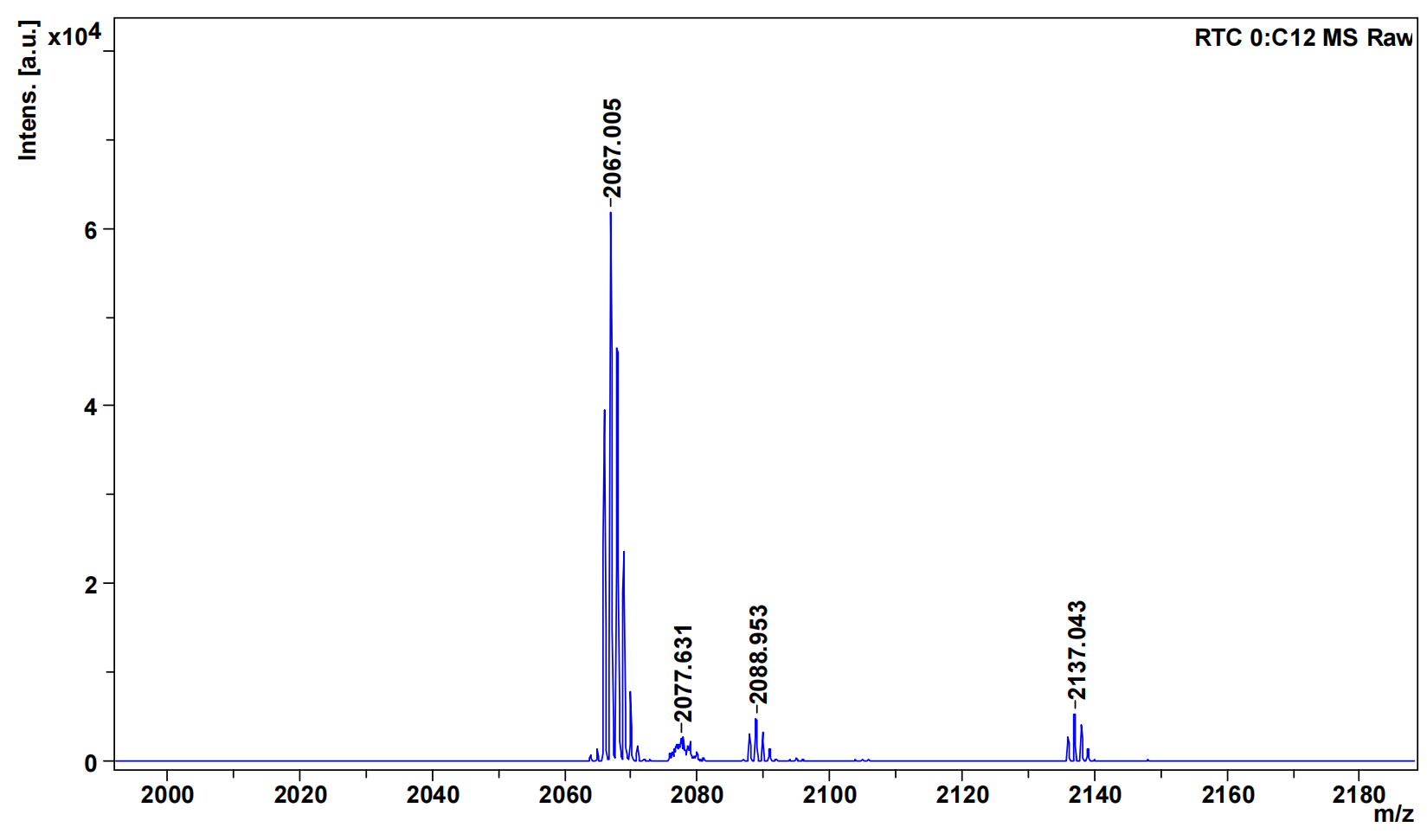


***Figure S18*** The HR-MALDI-TOF-MS of **RTC**.

**Synthesis of FT-RTC. RTC** (200 mg, 0.097 mmol) was dissolved in a CHCl_3_-MeOH (1:1, v/v) binary solvent mixture. Paraformaldehyde (70 mg, 24 eq.) was added and the reaction was stirred at 70 °C. After 3 hours, the reaction was cooled to room temperature and the solvent was then removed under vacuum. After adding chloroform to the resulting white solid, insoluble solid was filtered, and the solvent of remaining solution is removed to obtain the target product **FT-RTC**. Yield: 180 mg, 77.4%. ^1^H NMR (600 MHz, CDCl_3_, 298 K) δ (ppm): 8.52 (d, 24H, -ArH), 7.45 (d, 24H, -ArH), 4.23 (d, 12H, -ArCH_2_), 3.38-3.33 (m, 24H, -ArCH_2,_-NCH_2_N), 2.41 (s, 12H, CH on cyclohexane), 2.08 (d, 12H, CH_2_ on cyclohexane), 1.88 (d, 12H, CH_2_ on cyclohexane), 1.37-1.25 (m, 24H, CH_2_ on cyclohexane).^13^C NMR (151MHz, CDCl_3_, 298 K) δ (ppm): 171.29, 144.75, 134.88, 128.84, 127.56, 77.66, 69.00, 57.67, 29.47, 24.55. HR-MALDI-TOF-MS (DCTB): m/z [M+H]^+^ calcd for [C_138_H_145_N_24_]^+^: 2139.2118 , found : 2139.244.


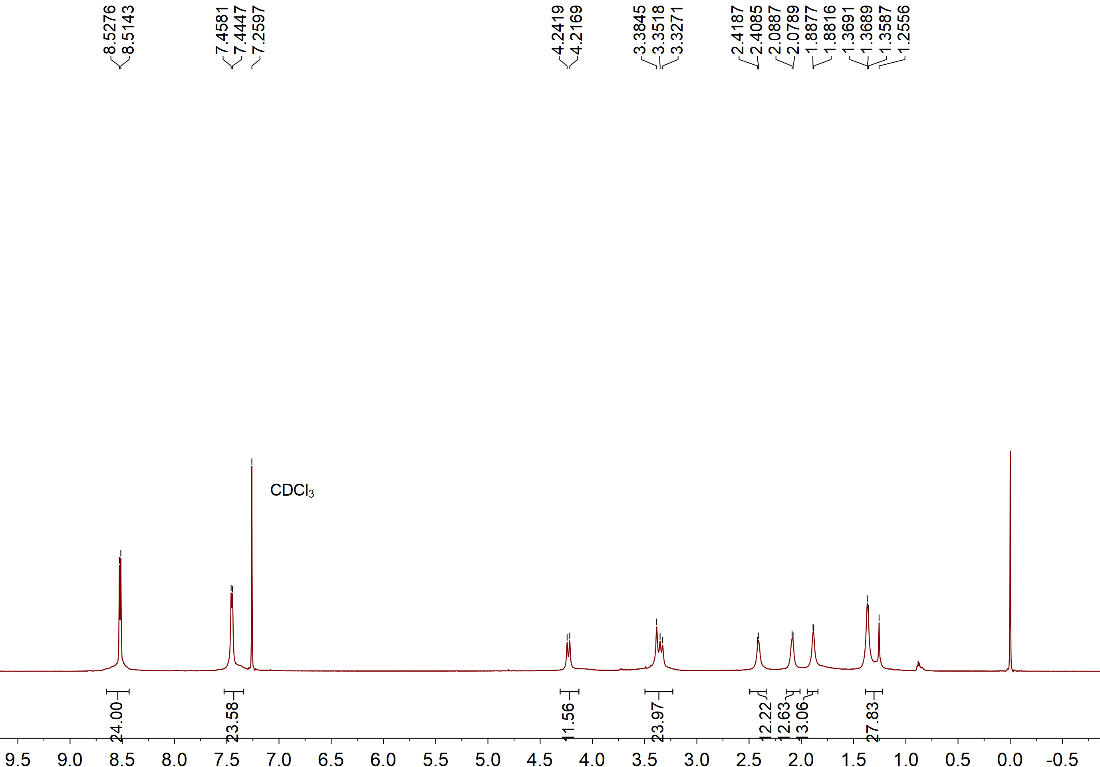


***Figure S19*** ^1^H NMR spectrum (600 MHz, CDCl_3_, 298 K) of **FT-RTC.**

**
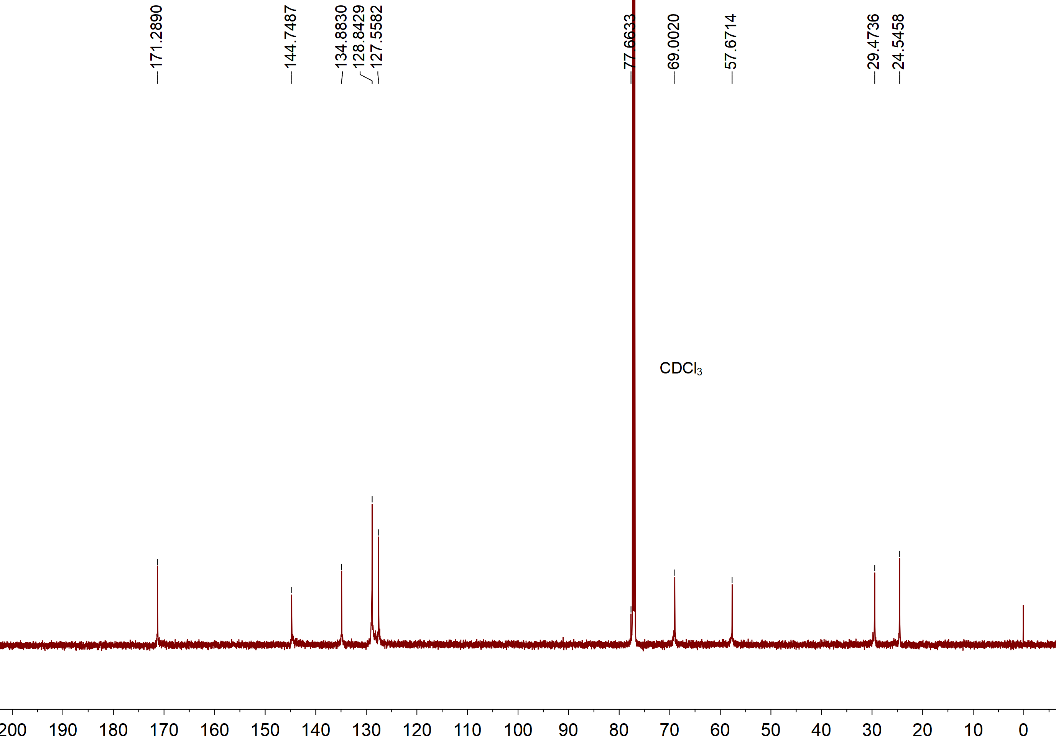
**

***Figure S20*** ^13^C NMR spectrum (151 MHz, CDCl_3_, 298 K) of **FT-RTC.**

**
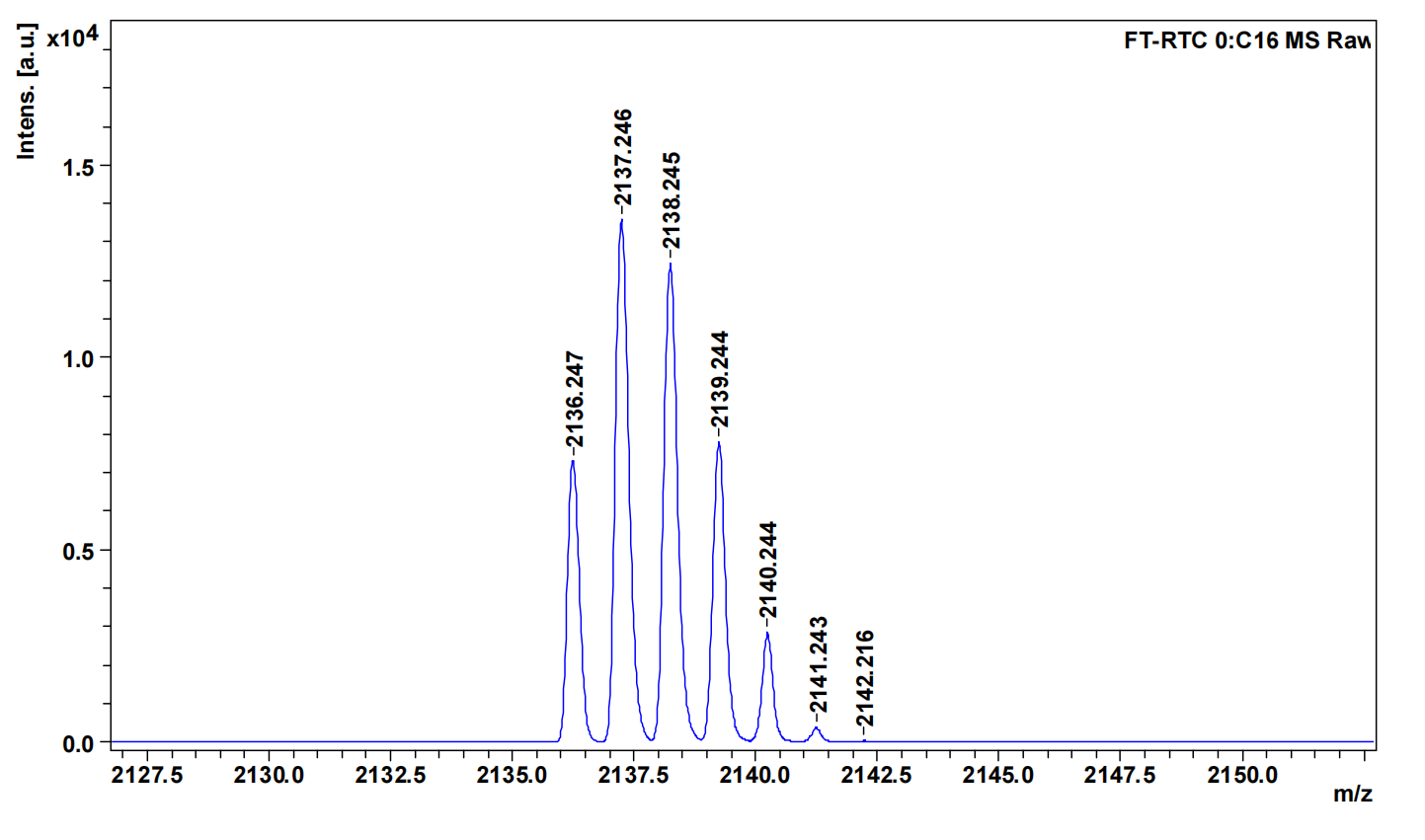
**

***Figure S21*** The HR-MALDI-TOF-MS of **FT-RTC.**

**Synthesis of** **OFT-RTC^6+^·6Br^-^.** **FT-RTC** (150 mg, 0.07 mmol) was dissolved in 1,2-dimethoxyethane (15 mL). *N*-Bromacetamide (76 mg, 0.546 mmol) was added in two portions (0.273 mmol each) with an interval of 15 min. After addition of the second portion, the reaction mixture was stirred for 3 hours. The product precipitated and was isolated by filtration. After washing by Et_2_O, **OFT-RTC^6+^6Br^−^** was purified and obtained. Yield: 179 mg, 98 %.^1^H NMR (600 MHz, DMSO-d_6_, 298 K) δ (ppm): 8.74 (s, 6H, -N=CH), 8.58 (d, 24H, -ArH), 7.65 (d, 24H, -ArH), 4.85 (s, 24H, -ArCH_2_), 3.61 (d, 12H, CH on cyclohexane), 2.30 (d, 12H, CH_2_ on cyclohexane), 1.92-1.73 (m, 12H, CH_2_ on cyclohexane), 1.58 (s, 12H, CH_2_ on cyclohexane), 1.29 (m, 12H, CH_2_ on cyclohexane). ^13^C NMR (151MHz, DMSO-d_6_, 298 K) δ (ppm): 171.03, 161.02, 139.08, 135.34, 129.31, 129.23, 67.76, 49.31, 27.01, 23.30.


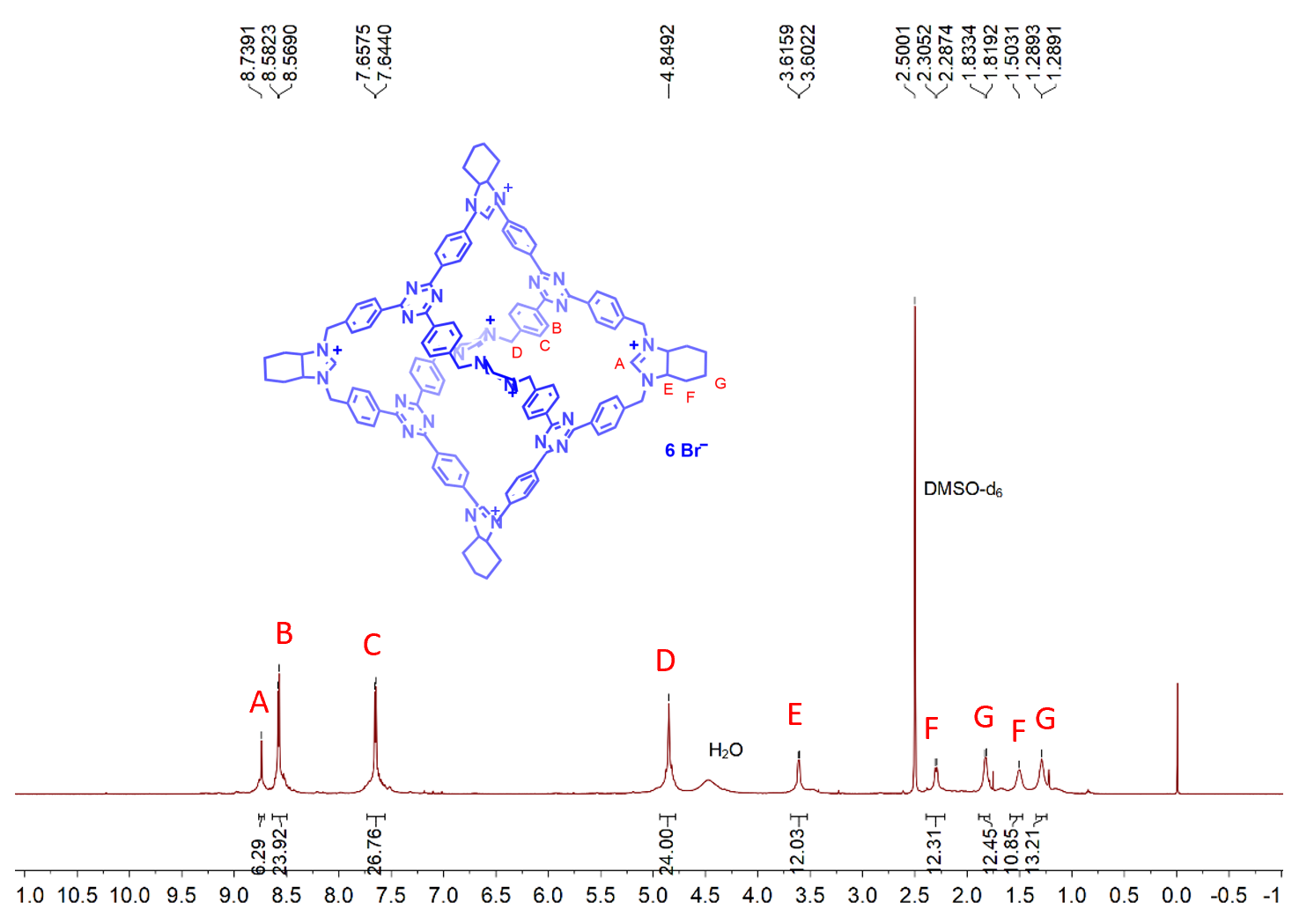


***Figure S22*** ^1^H NMR spectrum (600 MHz, DMSO-d_6_, 298 K) of **OFT-RTC^6+^6Br^-^.**
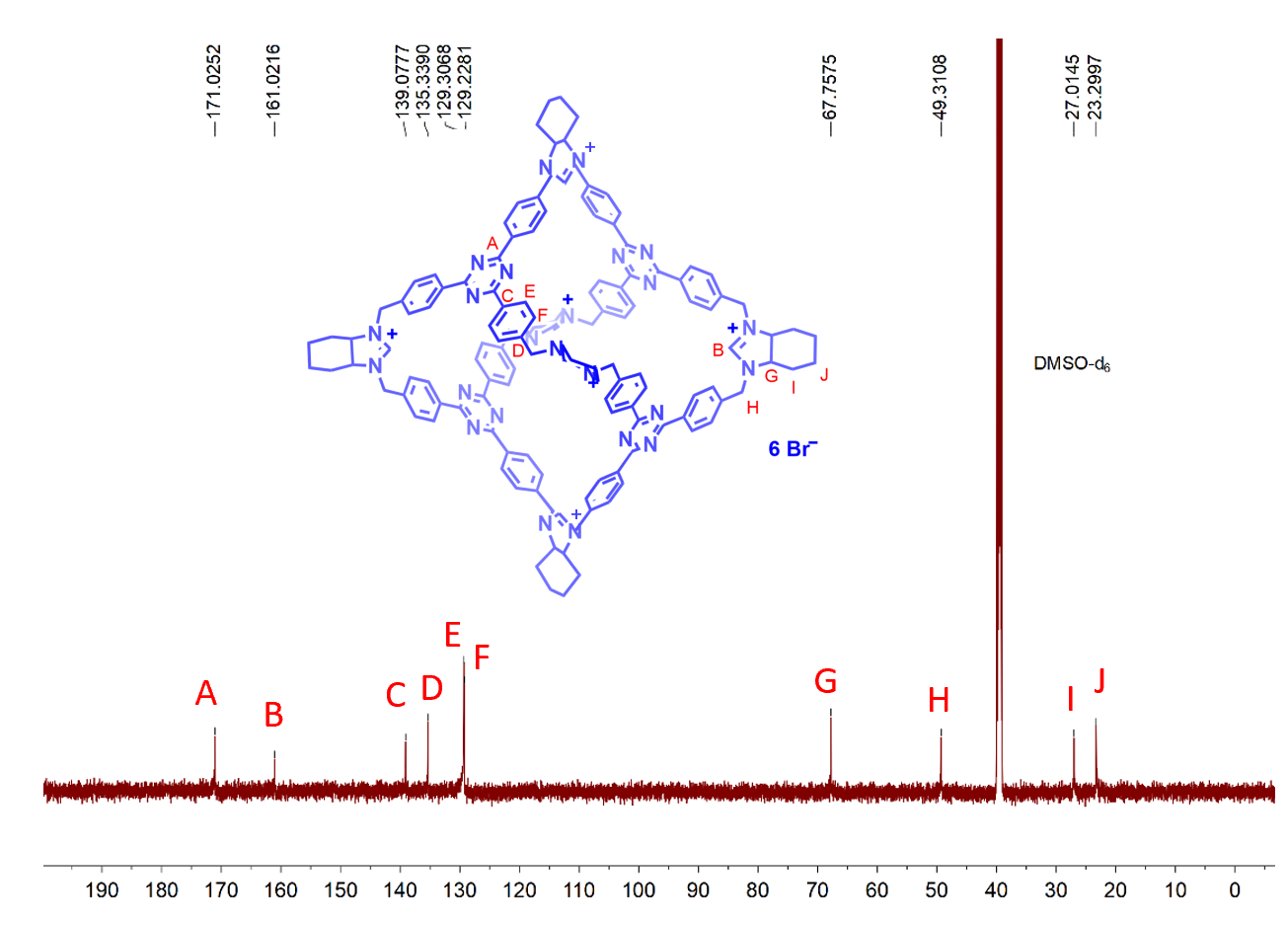


***Figure S23*** ^13^C NMR spectrum (151 MHz, DMSO-d_6_, 298 K) of **OFT-RTC^6+^6Br^-^.**

**
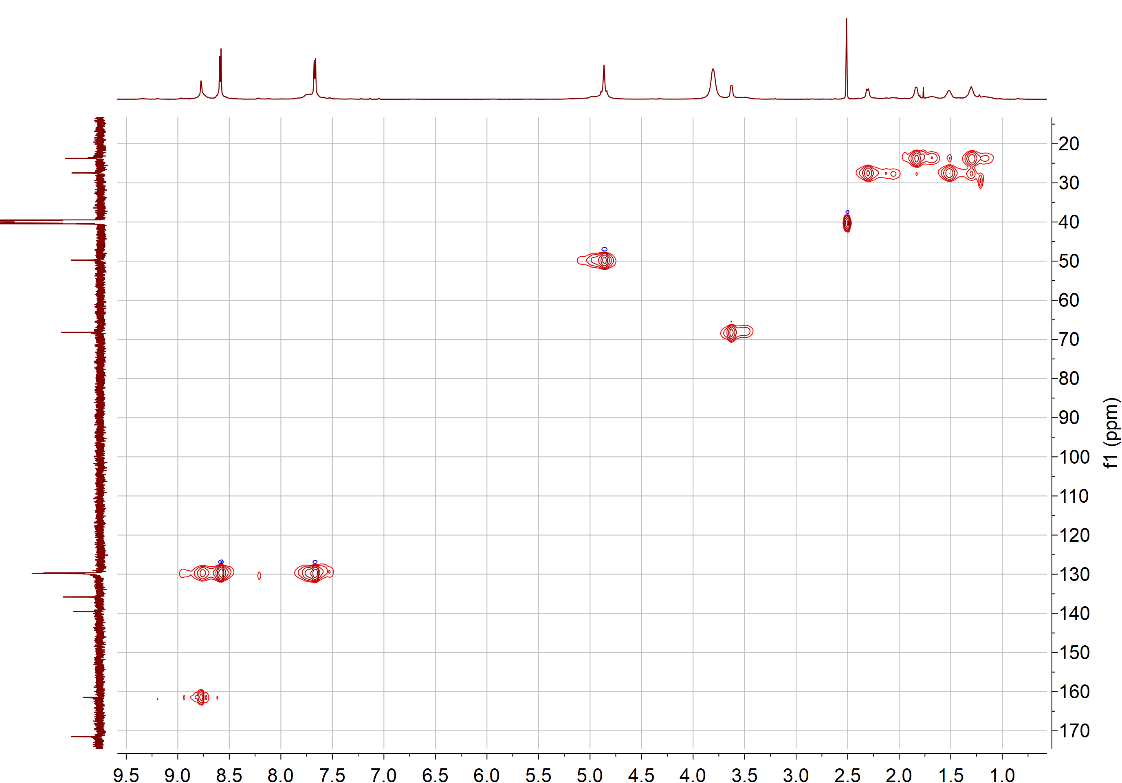
**

***Figure S24*** HSQC spectrum (DMSO-d_6_, 298 K) of **OFT-RTC^6+^6Br^-^.**

**Synthesis of (H_12_RCC1)^12+^12Br^-^. (H_12_RCC1)^12+^12Br^-^** was synthesized according to literature procedure^[9]^. ^1^H NMR (600 MHz, D_2_O, 298 K) *δ* (ppm): δ 7.68 (s, 12H, ArH), 4.41 (s, 24H, ArCH_2_), 3.51 (s, 24H, CH_2_).


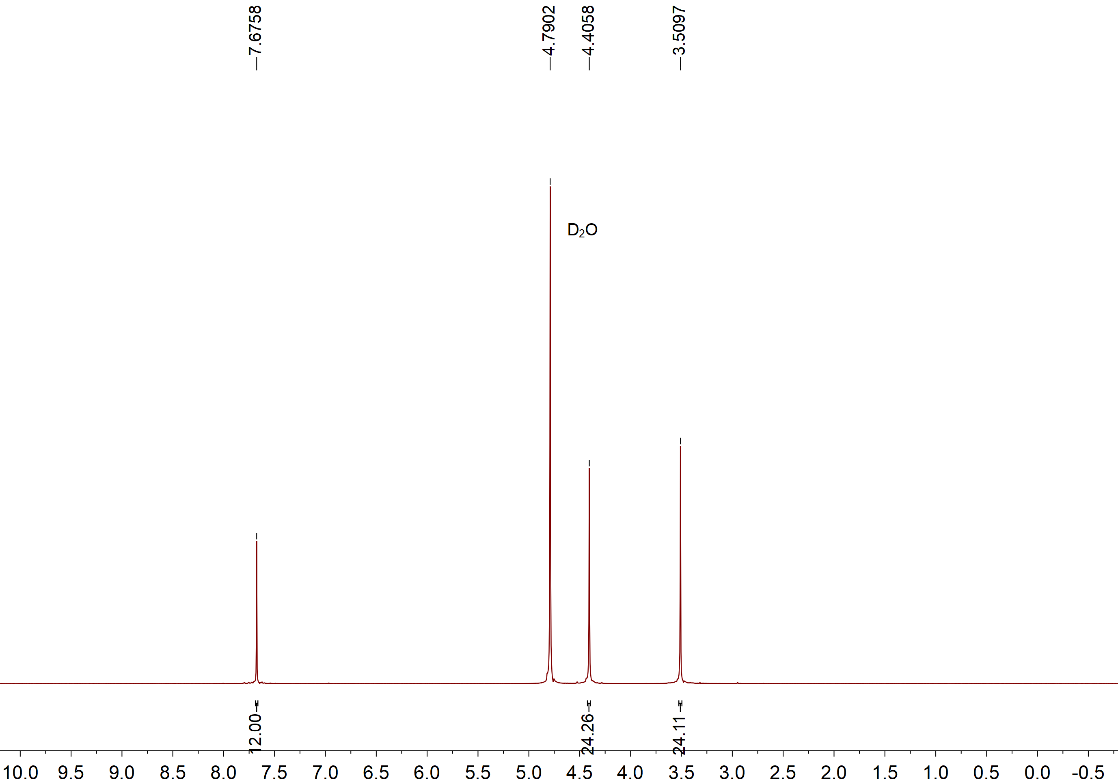


***Figure S25*** ^1^H NMR spectrum (600 MHz, D_2_O, 298 K) of **(H_12_RCC1)^12+^12Br^-^.**

**Synthesis of (H_12_RCC3)^12+^12Br^-^. (H_12_RCC3)^12+^12Br^-^** was synthesized according to literature procedure^[9]^. ^1^H NMR (600 MHz, D_2_O, 298 K) *δ* (ppm): δ 7.64 (s, 12H, ArH), 4.47 (d, 12H, ArCH_2_), 4.28 (d, 12H, ArCH_2_), 3.70 (s, 12H, CH on cyclohexane), 2.28 (s, 12H, CH_2_ on cyclohexane), 1.78 (m, 24H, CH_2_ on cyclohexane), 1.51 (s, 12H, CH_2_ on cyclohexane).


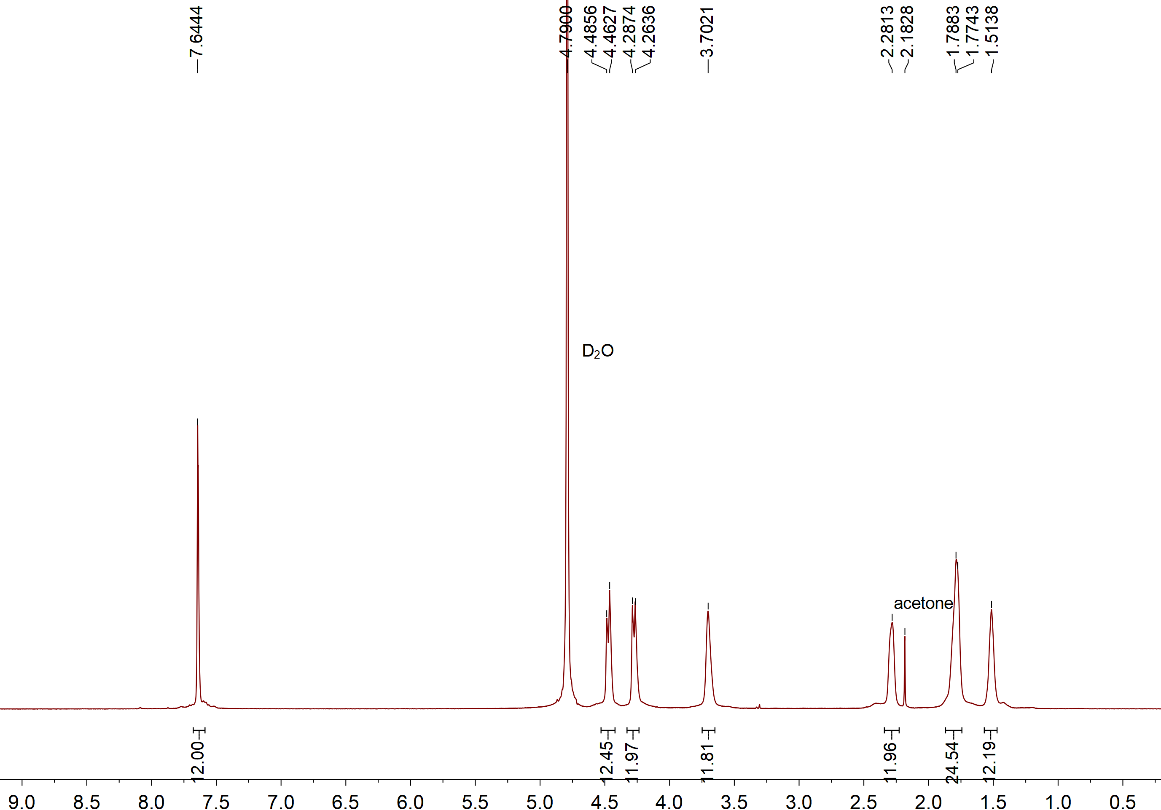


***Figure S26*** ^1^H NMR spectrum (600 MHz, D_2_O, 298 K) of **(H_12_RCC3)^12+^12Br^-^.**

**Synthesis of (H_12_RTC)^12+^12Br^-^. RTC** (160 mg, 0.077 mmol) was dissolved in CHCl_3_ (8 ml) by stirring. Hydrogen bromide (aqueous solution, 0.22 mL, 1.155 mmol, 15 eq.) was added dropwise. White precipitate appeared and the reaction mixture was stirred for a further 2 h at room temperature. The precipitate was collected by filtration then washed by CHCl_3_. ^1^H NMR (600 MHz, D_2_O, 298 K) *δ* (ppm): δ 8.72-7.51 (m, 48H, ArH), 4.41-3.60 (m, 24H, ArCH_2_), 2.53-1.44 (m, 60H, CH on cyclohexane).

**
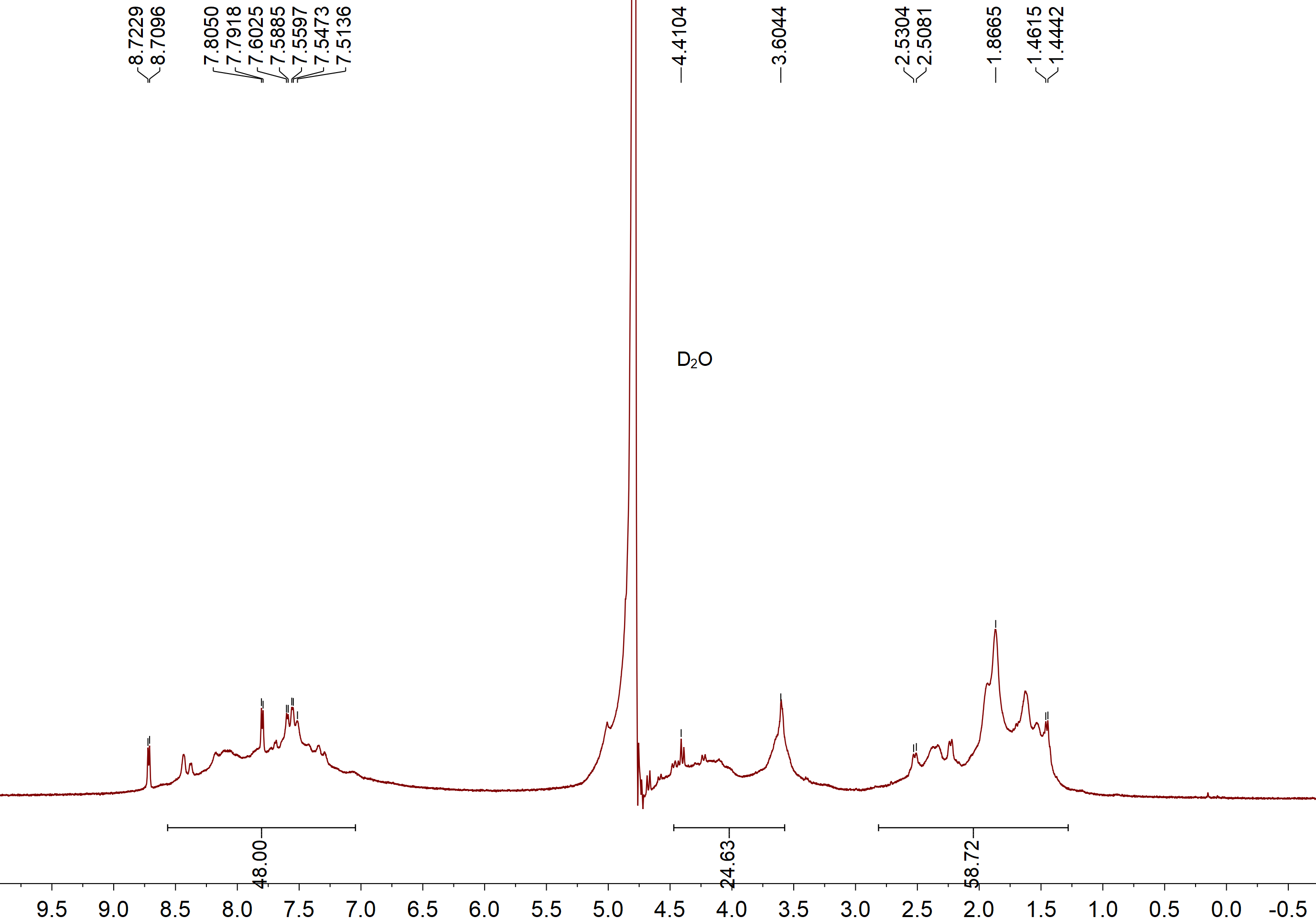
**

***Figure S27*** ^1^H NMR spectrum (600 MHz, D_2_O, 298 K) of **(H_12_RTC)^12+^12Br^-^.**

**3. X-ray Experimental Data**

**3.1 X-ray Experimental Data for OFT-RCC3^6+^6Br^-^**

The single crystal of **OFT-RCC3^6+^6Br^−^** was successfully obtained via diffusion of ethyl ether into a solution of cage in CH_2_Cl_2_-MeOH (1:1, v/v) binary solvent mixture. For each ionic cage molecule, six hydrogen atoms on the five membered ring of imidazoline salt inclined toward the cavity of the cage respectively, which interacted with sulfate anion locating inside the cavity through C-H···O hydrogen bonding interactions (2.110 Å and 2.287 Å). The majority of bromide anions are distributed disorderly around the cation cage, while C-H···Br hydrogen bonding interactions with the distance of 2.845 Å could be observed between one of the bromide ions and the cage skeleton. Of note, the presence of a possible sulfate anion within the cavity was incidental. It was not intentionally added, and its introduction may be attributed to the solvent or the use of anhydrous sodium sulfate during the drying process. The existence of the sulfate anion facilitated the crystallization of the ionic cage to some extent. The formation of single crystals of other ionic cages is, however, difficult to realize in the absence of the sulfate anion.

**
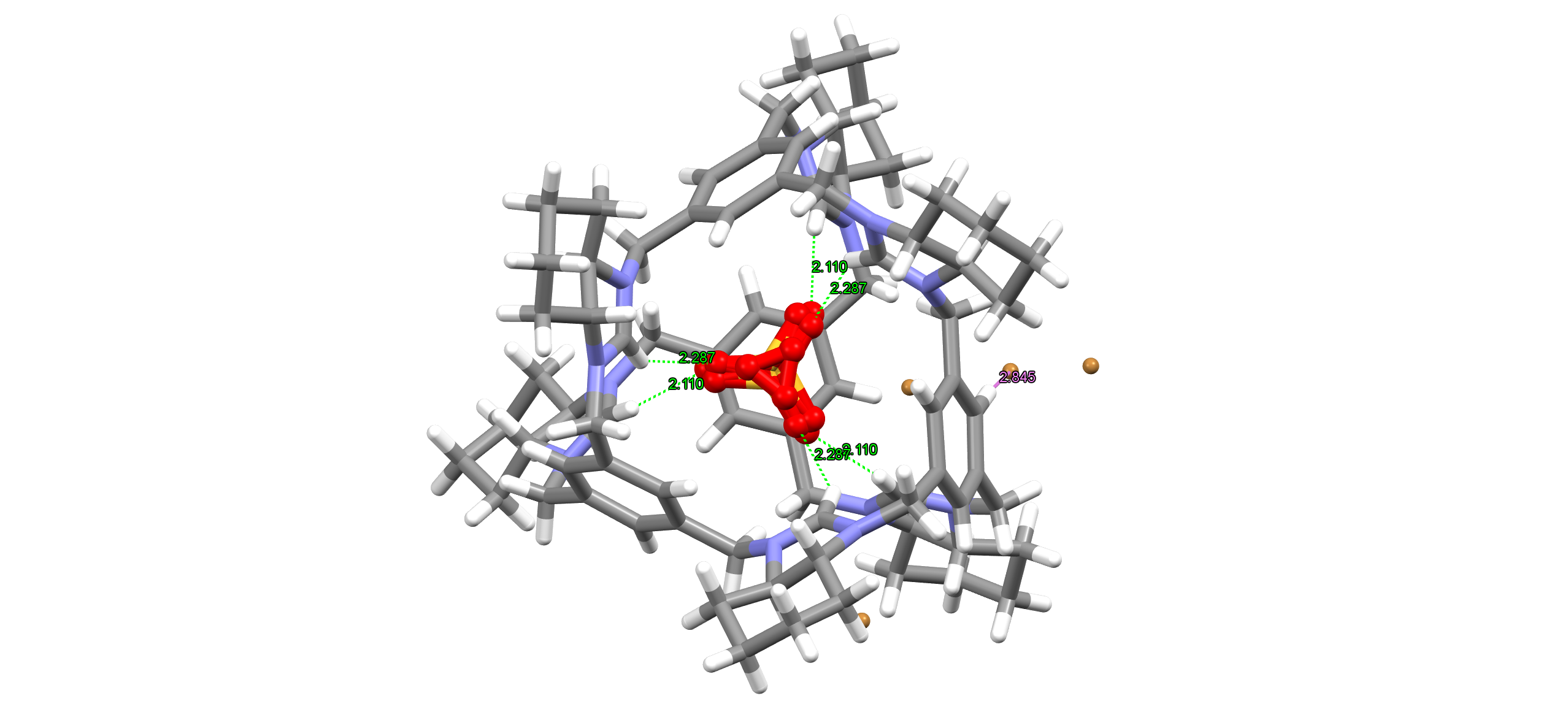
**

***Figure S28*** Single crystal structure of **OFT-RCC3^6+^6Br^-^**. C-H···Br hydrogen bond and C-H···O hydrogen bond are shown with purple and green dashed lines respectively.

**
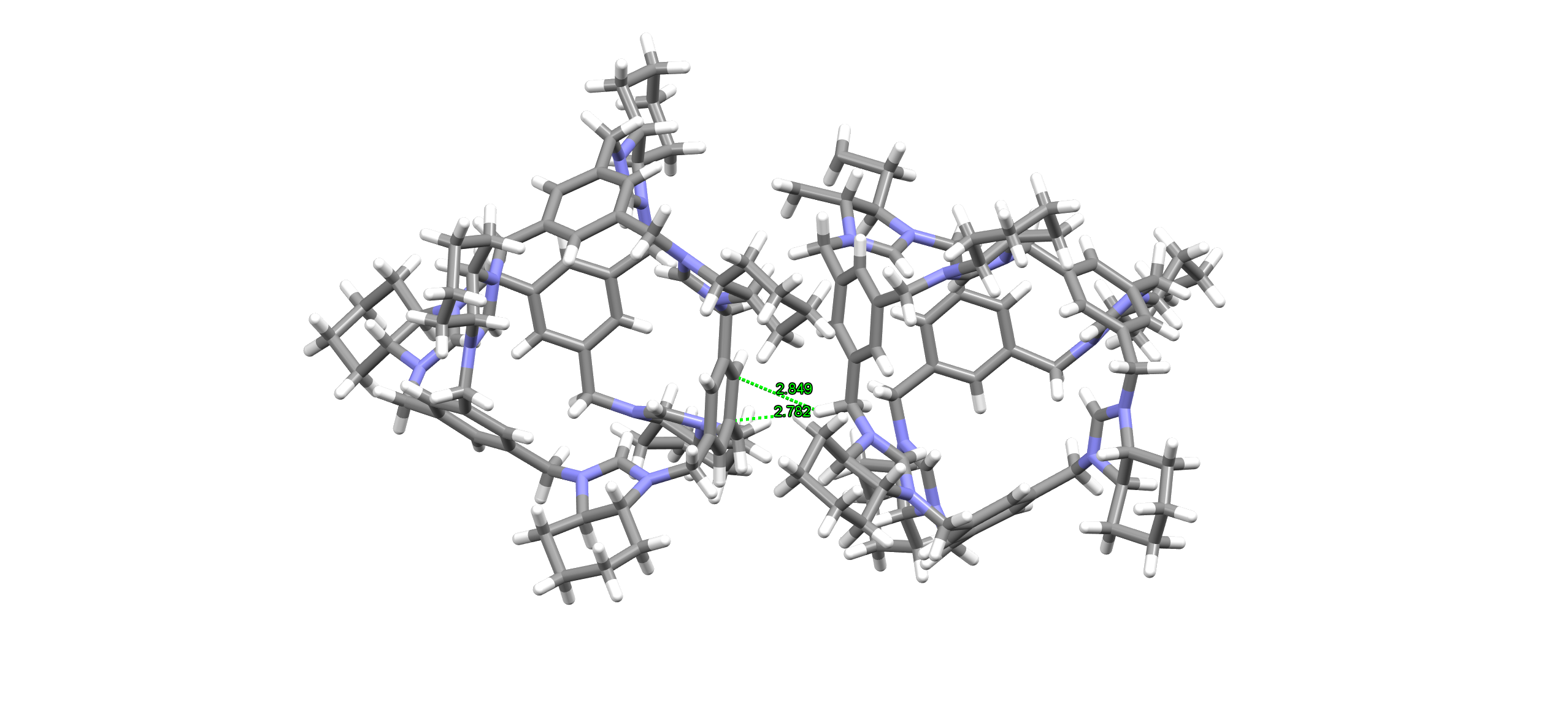
**

***Figure S29*** The packing modes of **OFT-RCC3^6+^6Br^-^**. Intermolecular C-H···π interactions are present with green dashed lines. Counter-anions are omitted for clarity.

***Table S1*** Single crystal X-ray data for **OFT-RCC3^6+^6Br^-^** (CCDC number: 2368267**)**

| Compound | **OFT-RCC3^6+^6Br^-^** |
| --- | --- |
| Formula | C_78_H_102_Br_8.13_N_12_O_4_S |
| Formula weight | 1953.69 |
| Temperature [K] | 150(2) |
| Crystal system | hexagonal |
| Space group (number) | $P6$ (168) |
| *a* [Å] | 20.9610(2) |
| *b* [Å] | 20.9610(2) |
| *c* [Å] | 13.9813(3) |
| α [°] | 90 |
| β [°] | 90 |
| γ [°] | 120 |
| Volume [Å^3^] | 5319.88(15) |
| *Z* | 2 |
| *ρ*_calc_ [gcm^−3^] | 1.220 |
| *μ* [mm^−1^] | 4.185 |
| F(000) | 1973 |
| Radiation | Cu*K_α_* (λ=1.54178 Å) |
| Reflections collected | 40210 |
| Final *R* indexes [*I*≥2σ(*I*)] | *R*_1_ = 0.0819 w*R*_2_ = 0.2011 |
| Final *R* indexes [all data] | *R*_1_ = 0.1010 w*R*_2_ = 0.2128 |
| Goodness-of-fit on *F*^2^ | 1.022 |

**3.2 X-ray Experimental Data for FT-RTC**

A colorless, block shaped crystal (**FT-RTC**) was successfully obtained via slow evaporation of solution of **FT-RTC** in THF.


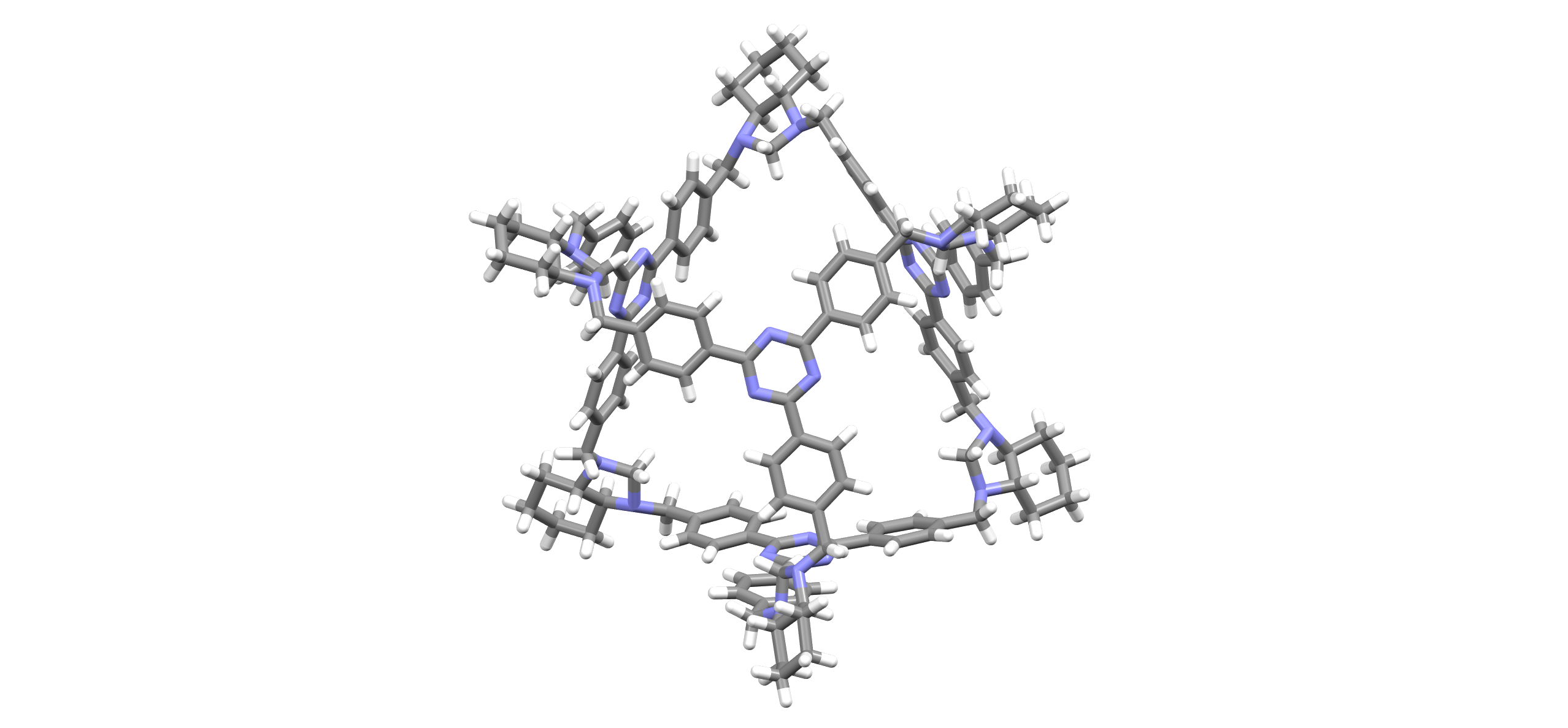


***Figure S30*** Single crystal structure of **FT-RTC**.

***Table S2*** Single crystal X-ray data for **FT-RTC** (CCDC number: 2368266**)**

| Compound | **FT-RTC** |
| --- | --- |
| Formula | C_138_H_144_N_24_ |
| Formula weight | 2138.76 |
| Temperature [K] | 150(2) |
| Crystal system | cubic |
| Space group (number) | $I2_{1}3$ (199) |
| *a* [Å] | 34.2127(9) |
| *b* [Å] | 34.2127(9) |
| *c* [Å] | 34.2127(9) |
| α [°] | 90 |
| β [°] | 90 |
| γ [°] | 90 |
| Volume [Å^3^] | 40046(3) |
| *Z* | 8 |
| *ρ*_calc_ [gcm^−3^] | 0.709 |
| *μ* [mm^−1^] | 0.333 |
| F(000) | 9120 |
| Radiation | Cu*K_α_* (λ=1.54178 Å) |
| Reflections collected | 22087 |
| Final *R* indexes [*I*≥2σ(*I*)] | *R*_1_ = 0.0874 w*R*_2_ = 0.1904 |
| Final *R* indexes [all data] | *R*_1_ = 0.1865 w*R*_2_ = 0.2369 |
| Goodness-of-fit on *F*^2^ | 1.026 |

**4. Iodine vapor uptake experiments**

**4.1 Iodine vapor uptake**

Time-dependent iodine vapor uptake experiments were carried out as follows: a glass vial (20 mL) packed with the activated materials (**OFT-RCC1^6+^6Br^-^**, **OFT-RCC3^6+^6Br^-^**, **OFT-RTC^6+^6Br^-^**, 30mg) was pre-weighed, and then was transferred to a larger wide-mouth jar (100 mL) containing a sufficient amount of solid iodine at the bottom. There was not physical contact between the adsorbents and iodine. Next, the jar was sealed and heated at 75 ℃ at ambient pressure in an oven. At different time intervals of adsorption, the iodine-loaded materials were cooled down to room temperature and weighed until its weight reached a steady value. The iodine uptake capacities were estimated by the weight gains: *C*=(m_2_-m_1_)/m_1_*100%, where *C* was the I_2_ uptake capacities, m_1_ and m_2_ were the masses of adsorbents before and after being exposed to I_2_ vapor, respectively. The capacities were determined by at least three parallel experiments. The pseudo-first-order and pseudo-second-order models were employed to help understand the adsorption kinetics. The linear forms of the two models can be expressed by the following equation (S-1 and S-2):

The pseudo-first order model (S-1)

$$\ln\text{(}\text{q}_{\text{e}}-\text{q}_{\text{t}}\text{)=}\ln\text{q}_{\text{e}}\text{-}\text{k}_{\text{1}}\text{∙t}$$

The pseudo-second order model(S-2)

$$\frac{\text{t}}{\text{q}_{\text{t}}}\text{=}\frac{\text{1}}{\text{k}_{\text{2}}\cdot\text{q}_{\text{e}}^{\text{2}}}\text{+}\frac{\text{t}}{\text{q}_{\text{e}}}$$

Where $\text{q}_{\text{e}}$ (g g^-1^) and $\text{q}_{\text{t}}$ (g g^-1^) refer to the adsorption capacity at equilibrium and at time t (min); k_1_ (min^−1^) and k_2_ (g g^−1^ min^−1^) are the adsorption rate constants for the pseudo-first-order and pseudo-second-order, respectively.

***
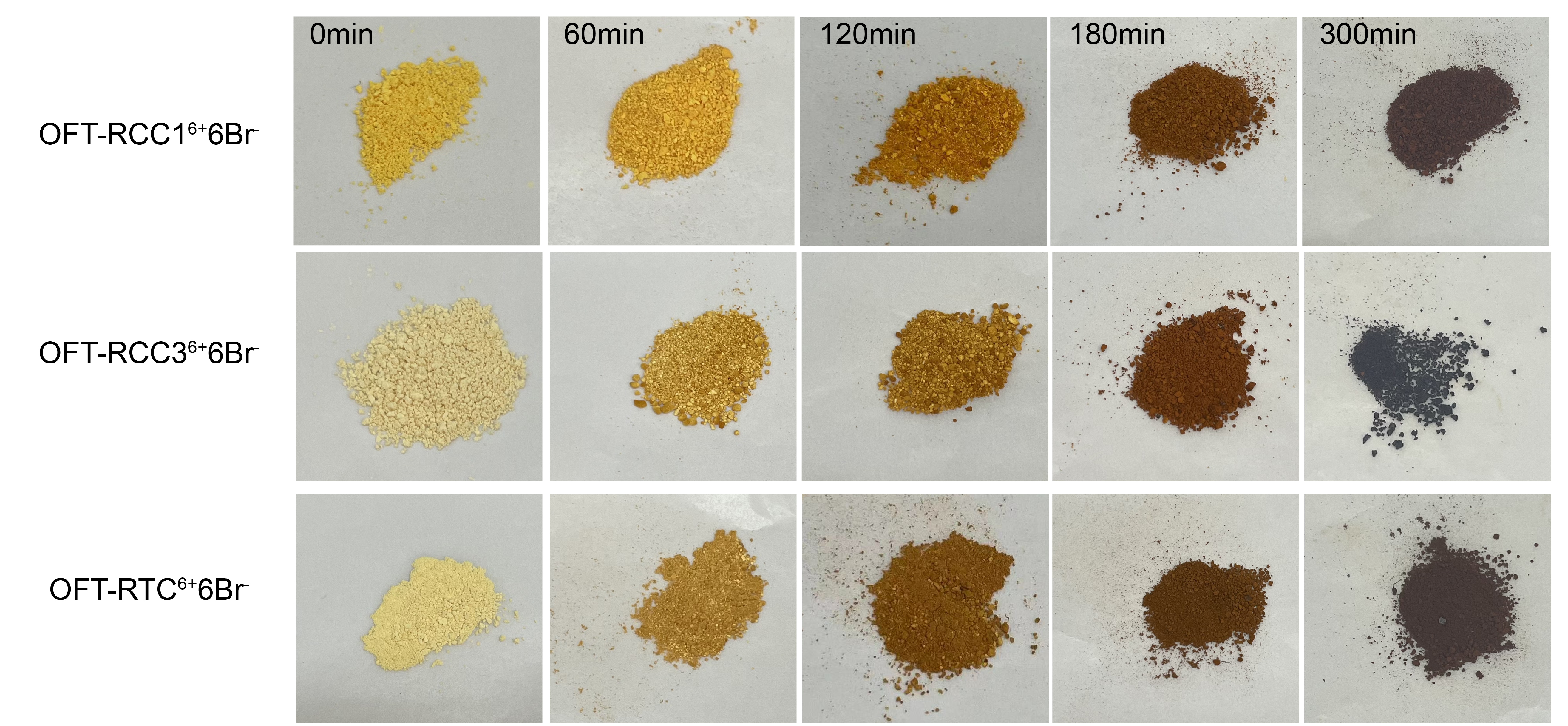
***

***Figure S31*** The photographs of **OFT-RCC1^6+^6Br^-^**, **OFT-RCC3^6+^6Br^-^**and **OFT-RTC^6+^6Br^-^** upon I_2_ vapor uptake at varying time intervals.


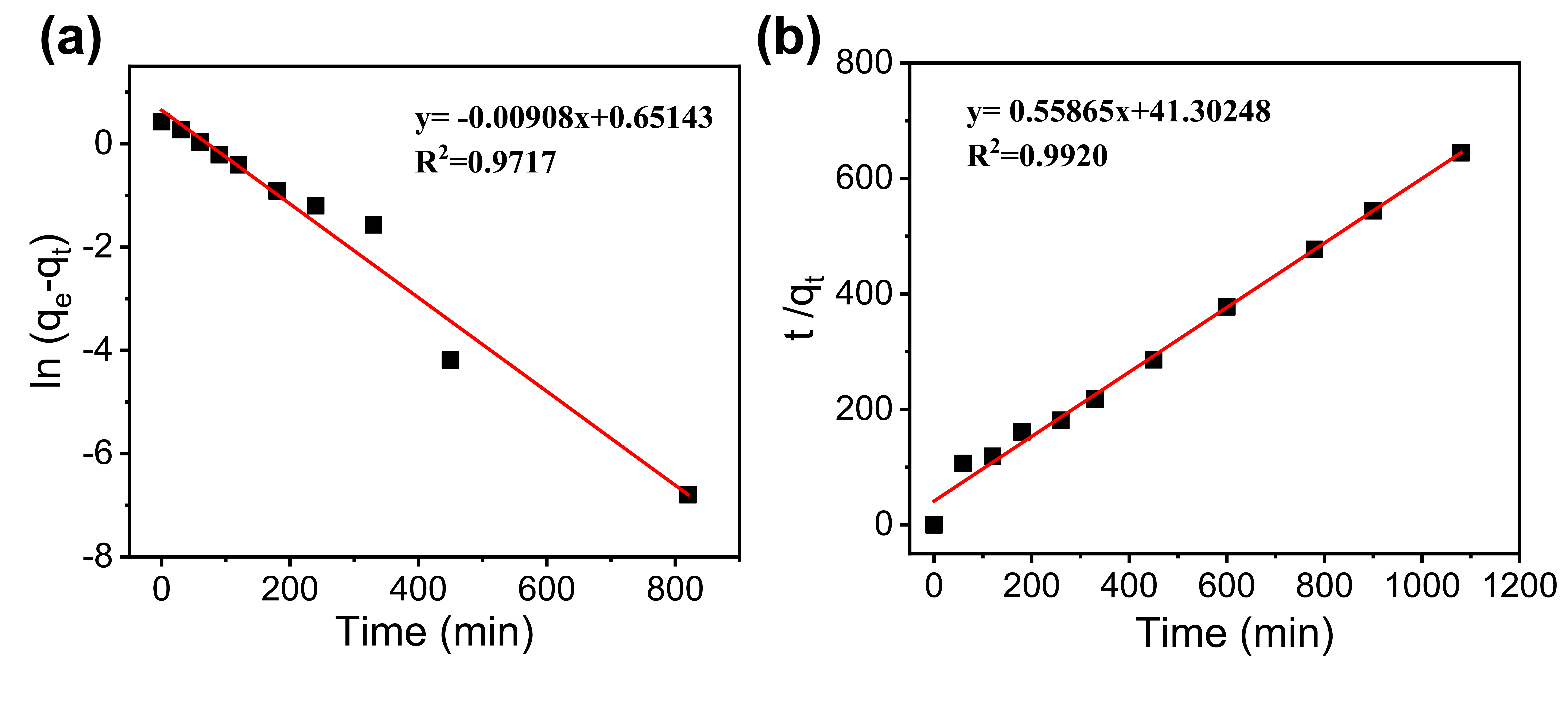


***Figure S32*** The kinetic fitting curves by pseudo-first-order kinetic (a) and pseudo-second-order model (b) for iodine vapor uptake of **OFT-RCC1^6+^6Br^-^**.


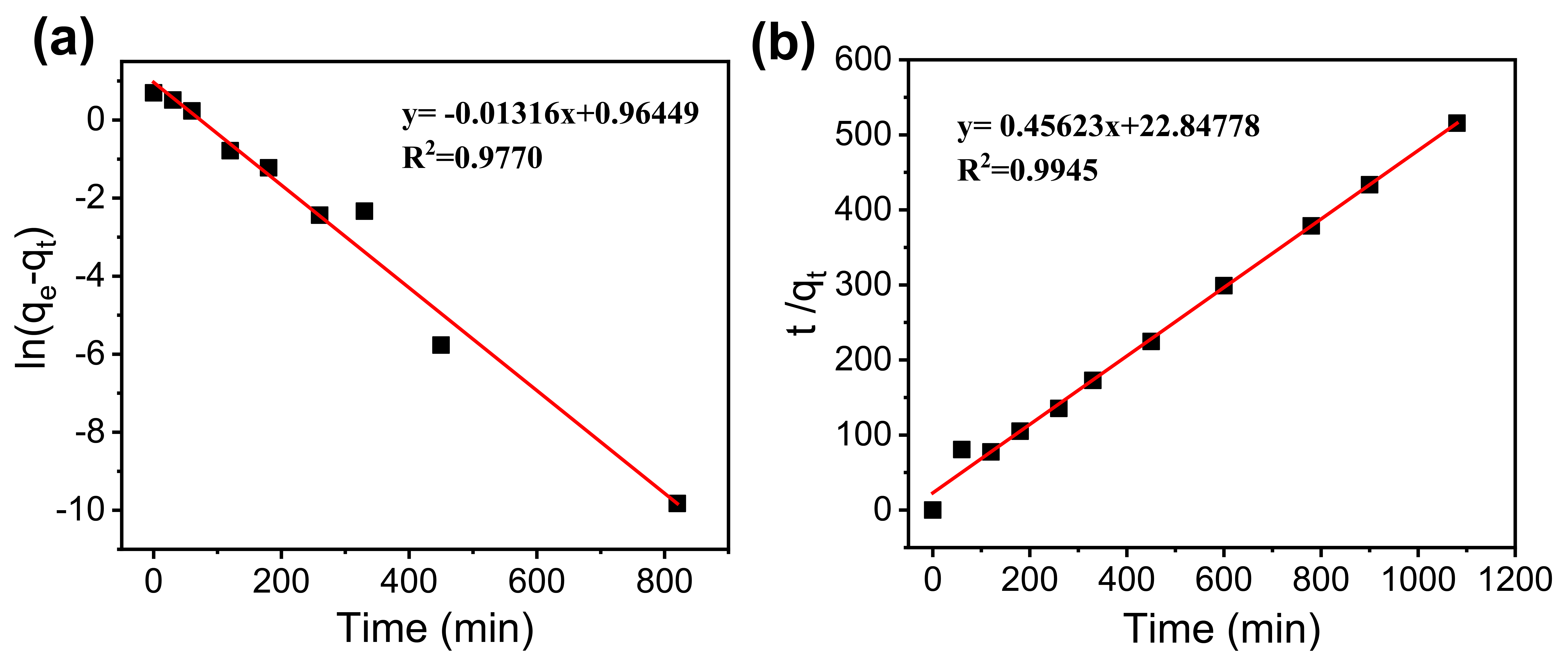


***Figure S33*** The kinetic fitting curves by pseudo-first-order kinetic (a) and pseudo-second-order model (b) for iodine vapor uptake of **OFT-RCC3^6+^6Br^-^**.


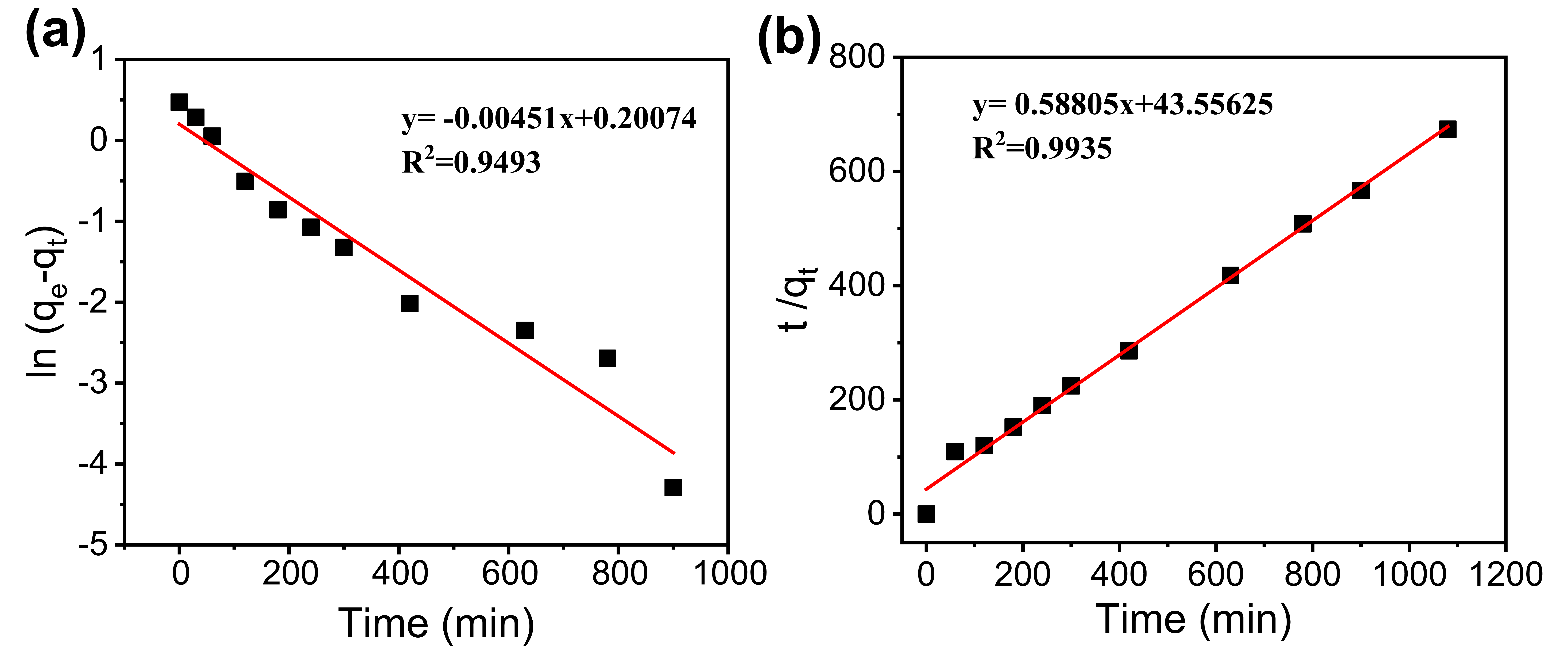


***Figure S34*** The kinetic fitting curves by pseudo-first-order kinetic (a) and pseudo-second-order model (b) for iodine vapor uptake of **OFT-RTC^6+^6Br^-^**.

***Table S3*** Kinetic parameters of I_2_ vapor uptake onto **OFT-RCC1^6+^6Br^-^**, **OFT-RCC3^6+^6Br^-^** and **OFT-RTC^6+^6Br^-^**.

| Adsorbent | Model | Parameters |  |
| --- | --- | --- | --- |
|  |  | q_exp_(g g^-1^) | 1.5425 |
|  |  | q_e,cal_ (g g^-1^) | 1.9183 |
|  | Pseudo-first-order | k_1_ (min^-1^) | 0.0091 |
| OFT-RCC1^6+^6Br^-^ |  | R^2^ | 0.9717 |
|  |  | q_e,cal_ (g g^-1^) | 1.7900 |
|  | Pseudo-second-order | k_2_ (g g^-1^min^-1^) | 0.0076 |
|  |  | R^2^ | 0.9920 |
|  |  | q_exp_(g g^-1^) | 1.9523 |
|  |  | q_e,cal_ (g g^-1^) | 2.6234 |
|  | Pseudo-first-order | k_1_ (min^-1^) | 0.0132 |
| OFT-RCC3^6+^6Br^-^ |  | R^2^ | 0.9770 |
|  |  | q_e,cal_ (g g^-1^) | 2.1919 |
|  | Pseudo-second-order | k_2_ (g g^-1^min^-1^) | 0.0091 |
|  |  | R^2^ | 0.9945 |
|  |  | q_exp_(g g^-1^) | 1.6098 |
|  |  | q_e,cal_ (g g^-1^) | 1.2223 |
|  | Pseudo-first-order | k_1_ (min^-1^) | 0.0045 |
| OFT-RTC^6+^6Br^-^ |  | R^2^ | 0.9493 |
|  |  | q_e,cal_ (g g^-1^) | 1.7005 |
|  | Pseudo-second-order | k_2_ (g g^-1^min^-1^) | 0.0079 |
|  |  | R^2^ | 0.9935 |

***
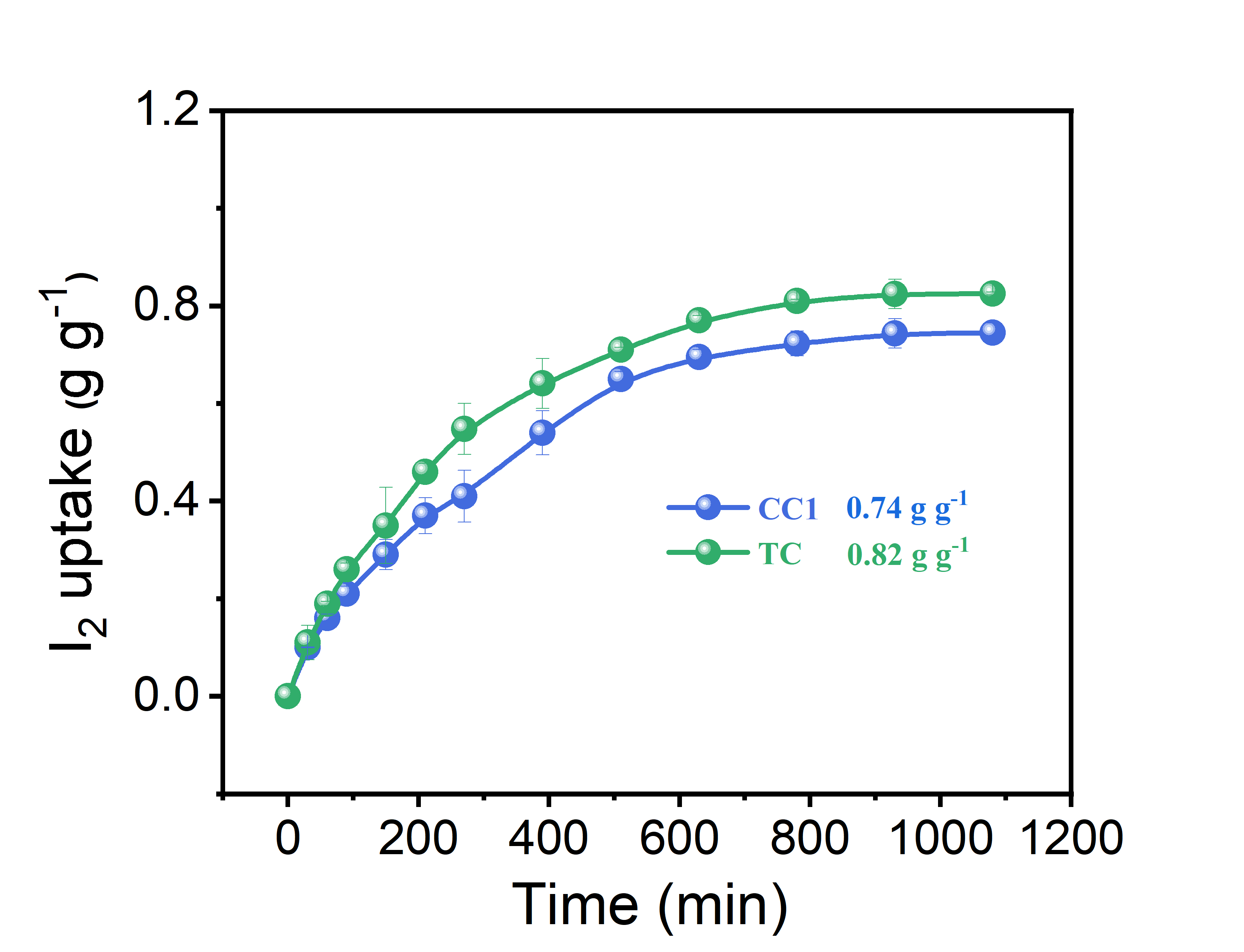
***

***Figure S35*** Time-dependent plots of iodine vapor uptake at 75 °C and under ambient pressure.


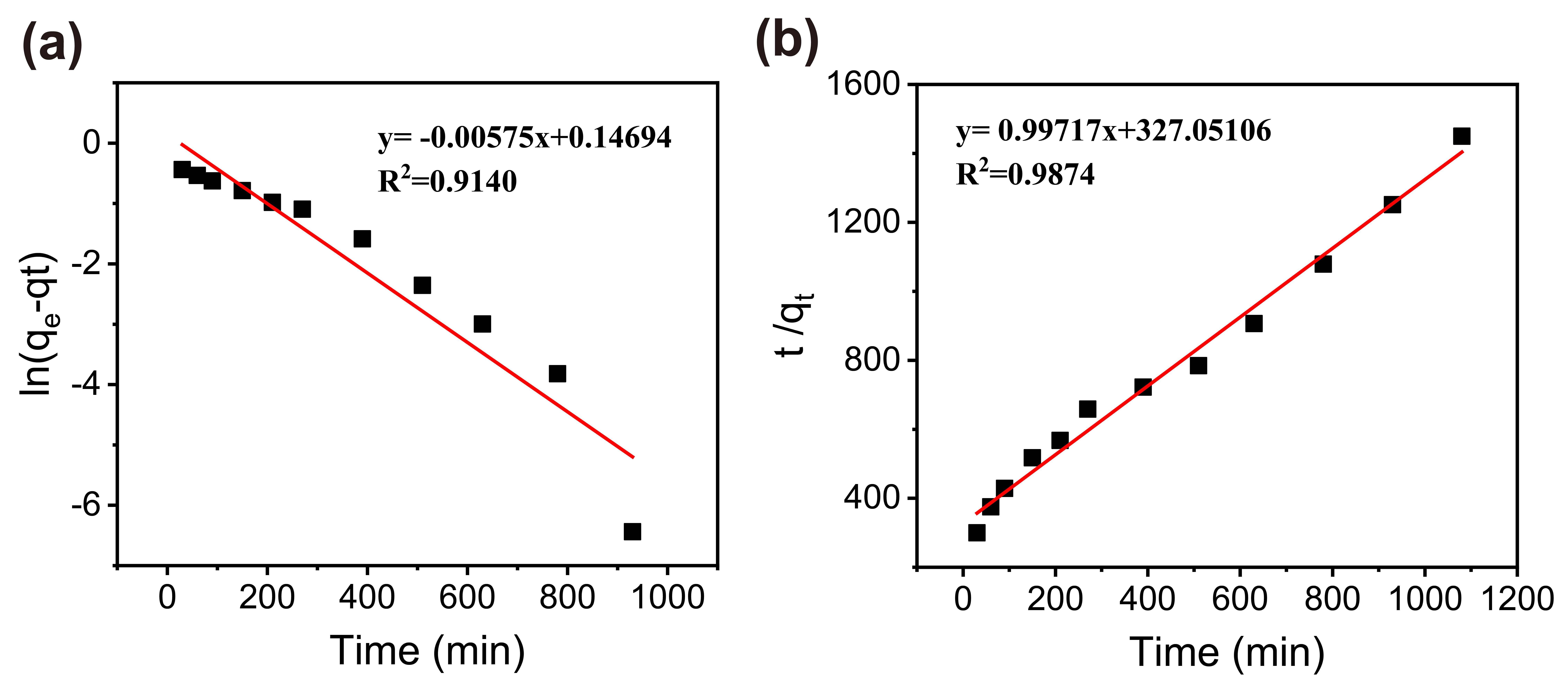


***Figure S36*** The kinetic fitting curves by pseudo-first-order kinetic (a) and pseudo-second-order model (b) for iodine vapor uptake of **CC1**.


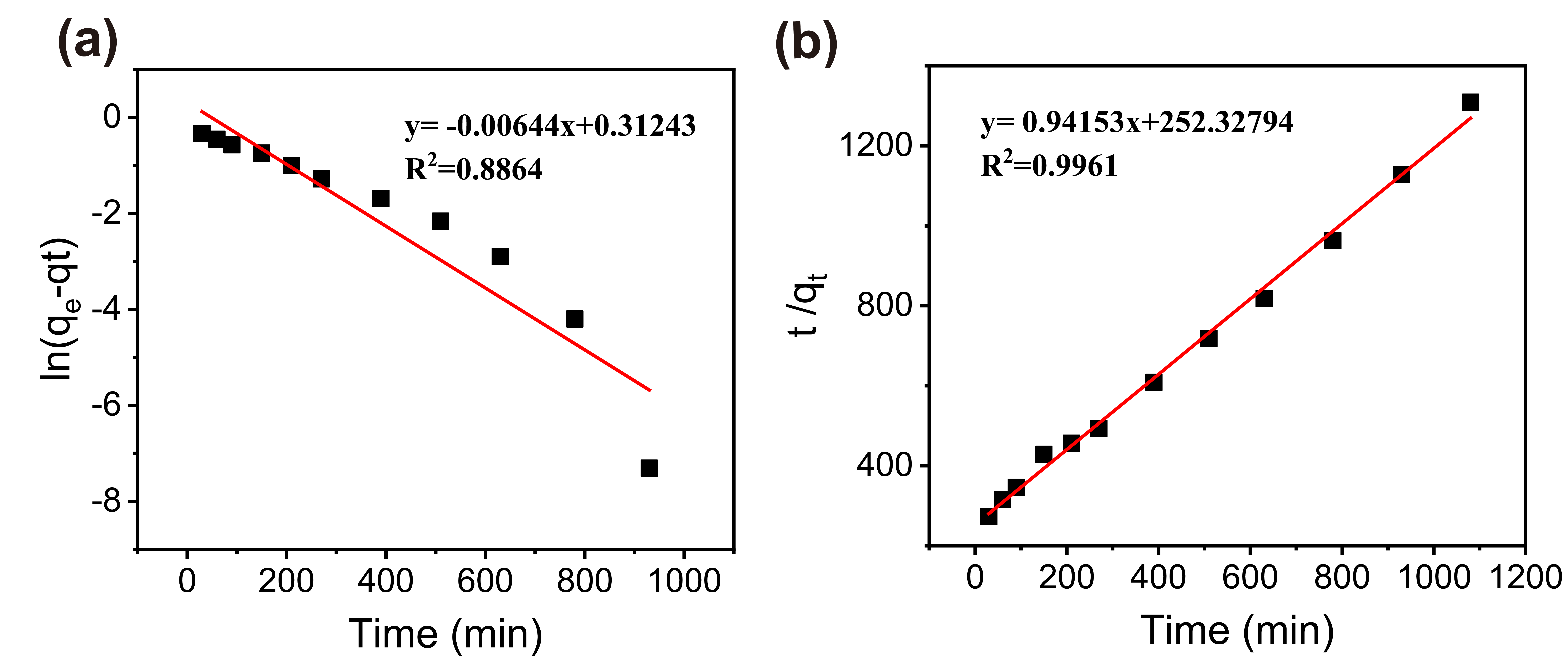


***Figure S37*** The kinetic fitting curves by pseudo-first-order kinetic (a) and pseudo-second-order model (b) for iodine vapor uptake of **TC**.

***Table S4*** Kinetic parameters of I_2_ vapor uptake onto **CC1** and **TC**

| Adsorbent | Model | Parameters |  | |
| --- | --- | --- | --- | --- |
|  |  | q_exp_(g g^-1^) | | 0.7449 |
|  |  | q_e,cal_ (g g^-1^) | | 1.1583 |
|  | Pseudo-first-order | k_1_ (min^-1^) | | 0.0058 |
| CC1 |  | R^2^ | | 0.9140 |
|  |  | q_e,cal_ (g g^-1^) | | 1.0028 |
|  | Pseudo-second-order | k_2_ (g g^-1^min^-1^) | | 0.0030 |
|  |  | R^2^ | | 0.9874 |
|  |  | q_exp_(g g^-1^) | | 0.8250 |
|  |  | q_e,cal_ (g g^-1^) | | 1.3667 |
|  | Pseudo-first-order | k_1_ (min^-1^) | | 0.00644 |
| TC |  | R^2^ | | 0.8864 |
|  |  | q_e,cal_ (g g^-1^) | | 1.0621 |
|  | Pseudo-second-order | k_2_ (g g^-1^min^-1^) | | 0.0035 |
|  |  | R^2^ | | 0.9961 |


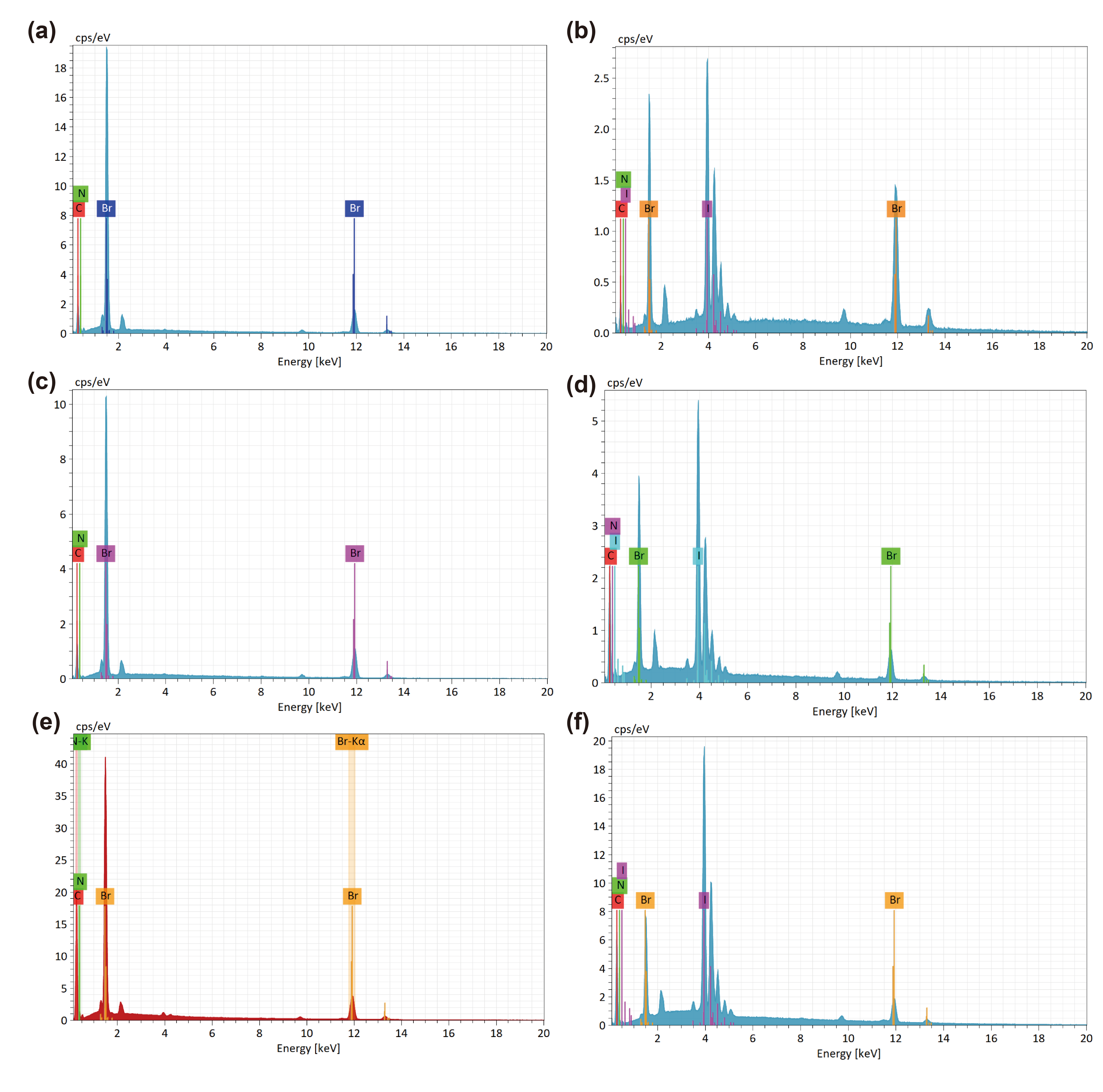


***Figure S38*** Energy-dispersive spectroscopy (EDS) analysis profiles of **OFT-RCC1^6+^6Br^-^**, **OFT-RCC3^6+^6Br^-^**, **OFT-RTC^6+^6Br^-^**before (a, c, e) and after (b, d, f) adsorption of iodine.


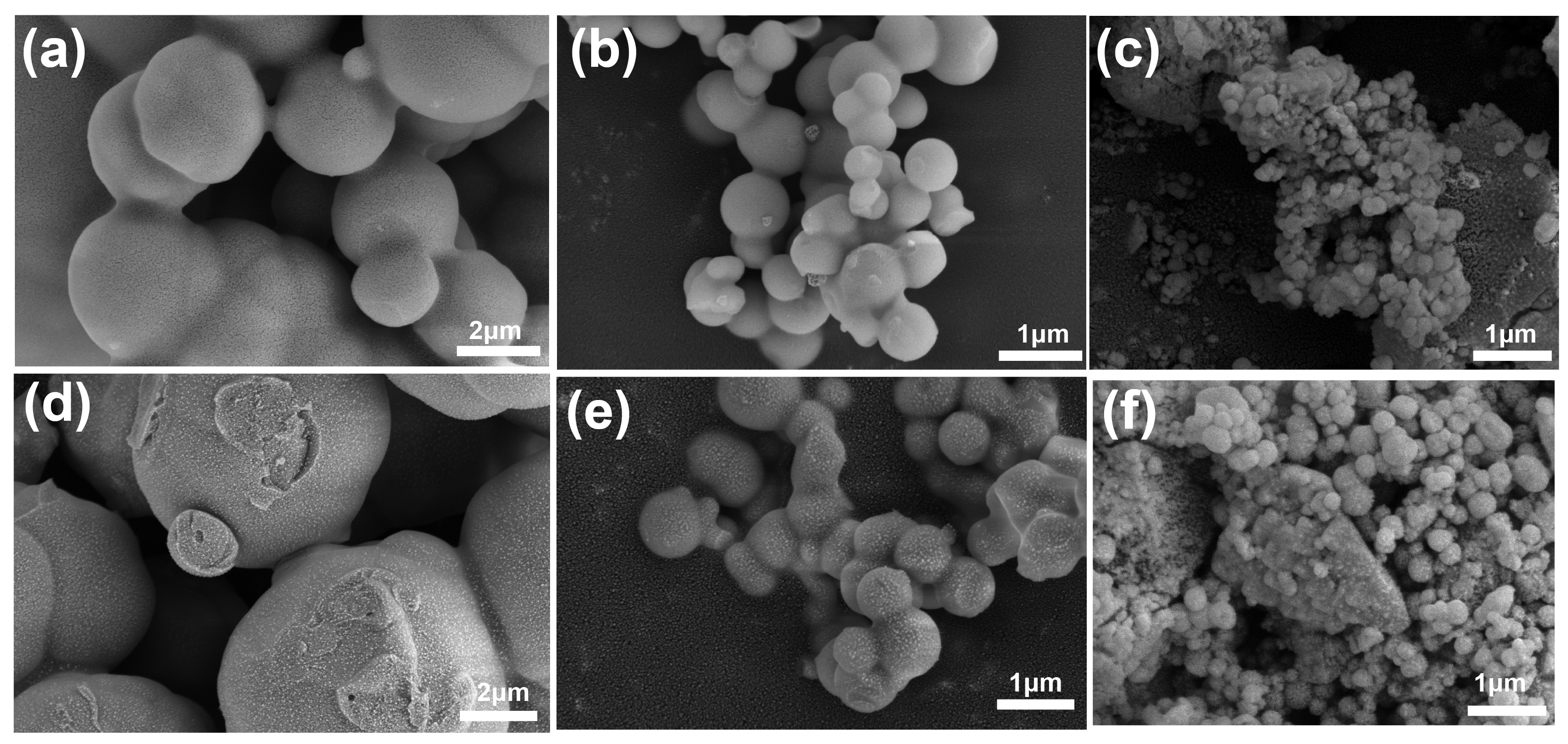


***Figure S39*** SEM profiles of **OFT-RCC1^6+^6Br^-^**, **OFT-RCC3^6+^6Br^-^**, **OFT-RTC^6+^6Br^-^**before (a, b, c) and after (d, e, f) adsorption of iodine.





***Figure S40*** TGA analysis of **OFT-RCC1^6+^6Br^-^**(a), **OFT-RCC3^6+^6Br^-^**(b), **OFT-RTC^6+^6Br^-^** (c) before and after adsorption of iodine.





***Figure S41*** (a) N_2_ adsorption isotherm for **OFT-RCC1^6+^6Br****^−^**, with the calculated BET surface area value at 1.08 m^2^ /g. (b) N_2_ adsorption isotherm for **OFT-RCC3^6+^6Br^−^**, with the calculated BET surface area value at 3.78 m^2^ /g. (c) N_2_ adsorption isotherm for **OFT-RTC^6+^6Br^−^**, with the calculated BET surface area value at 15.85 m^2^ /g.





***Figure S42*** Powder X-ray diffraction patterns of OFT-RCC1^6+^6Br^-^ (a), OFT-RCC3^6+^6Br^-^ (b), OFT-RTC^6+^6Br^-^ (c) before and after adsorption of iodine vapor. After the uptake of iodine, the PXRD of all three adsorbents showed no clear diffraction peaks, demonstrating the loss of the crystalline structure of OFT-RCC1^6+^6Br^-^ and good dispersion of iodine molecules in I_2_@ OFT-RCC1^6+^6Br^-^.


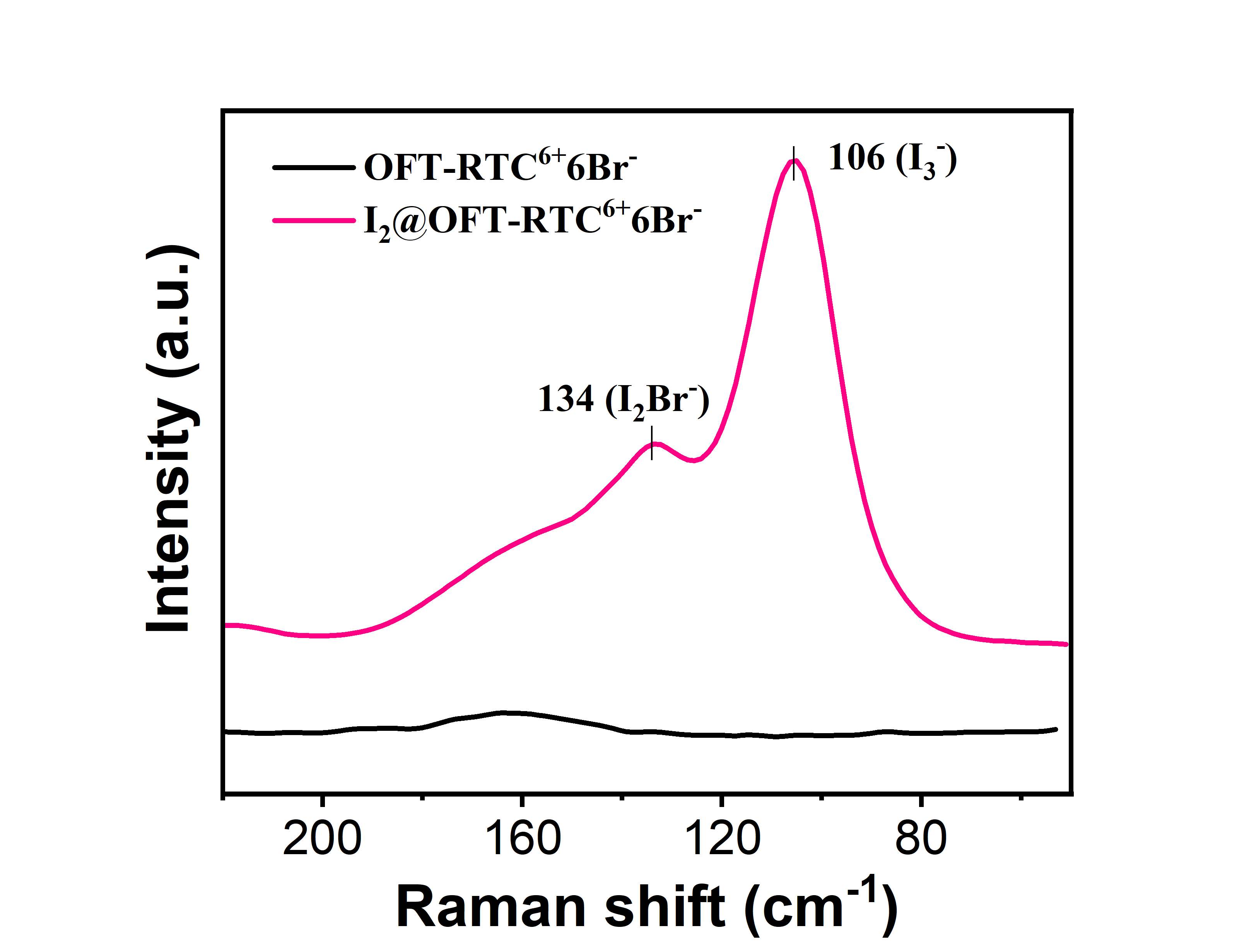


***Figure S43*** The Raman spectra of OFT-RTC^6+^6Br^-^ before and after iodine adsorption


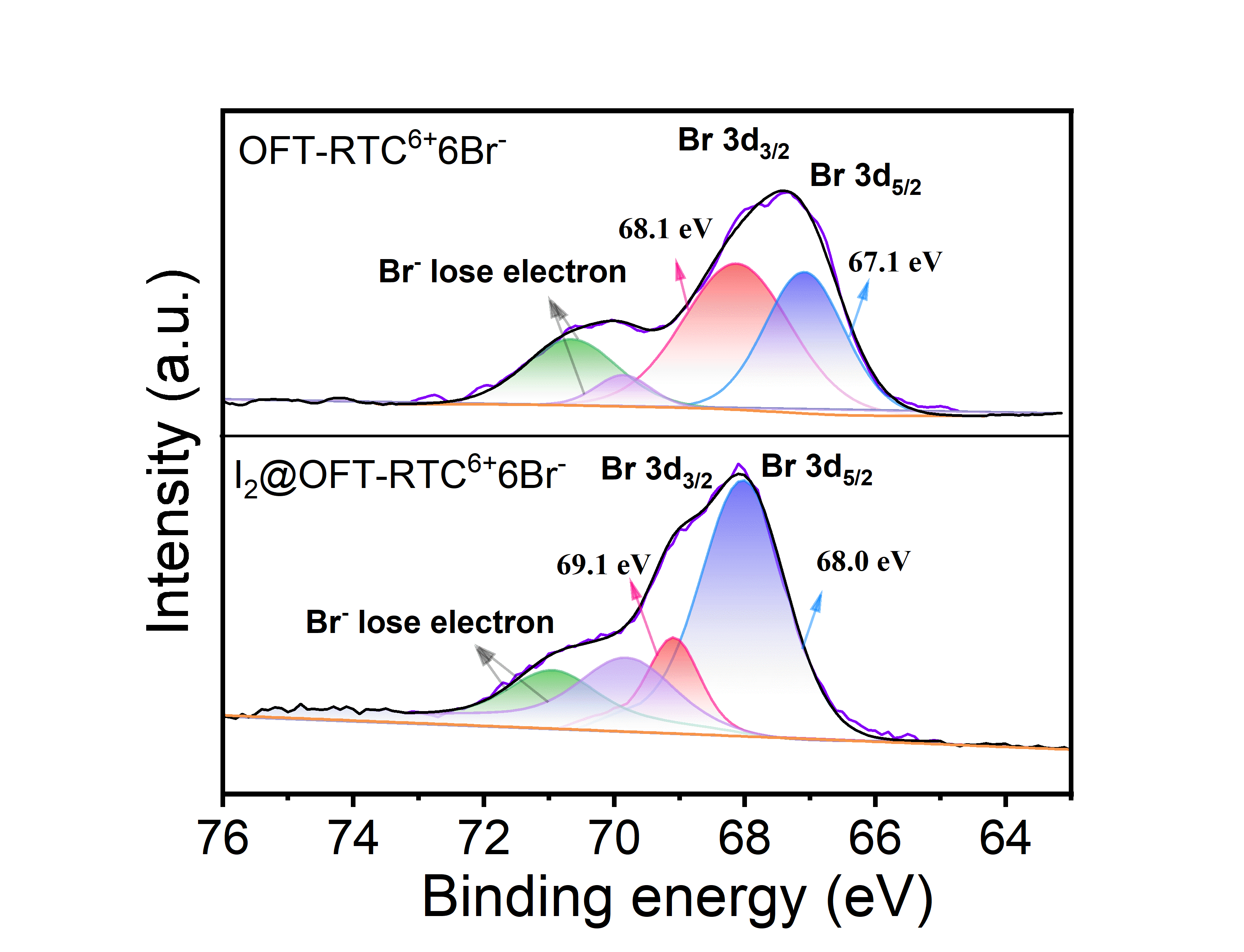


***Figure S44*** XPS analysis profiles of Br 3d for **OFT-RTC^6+^6Br^-^**before and after adsorption of iodine.

**
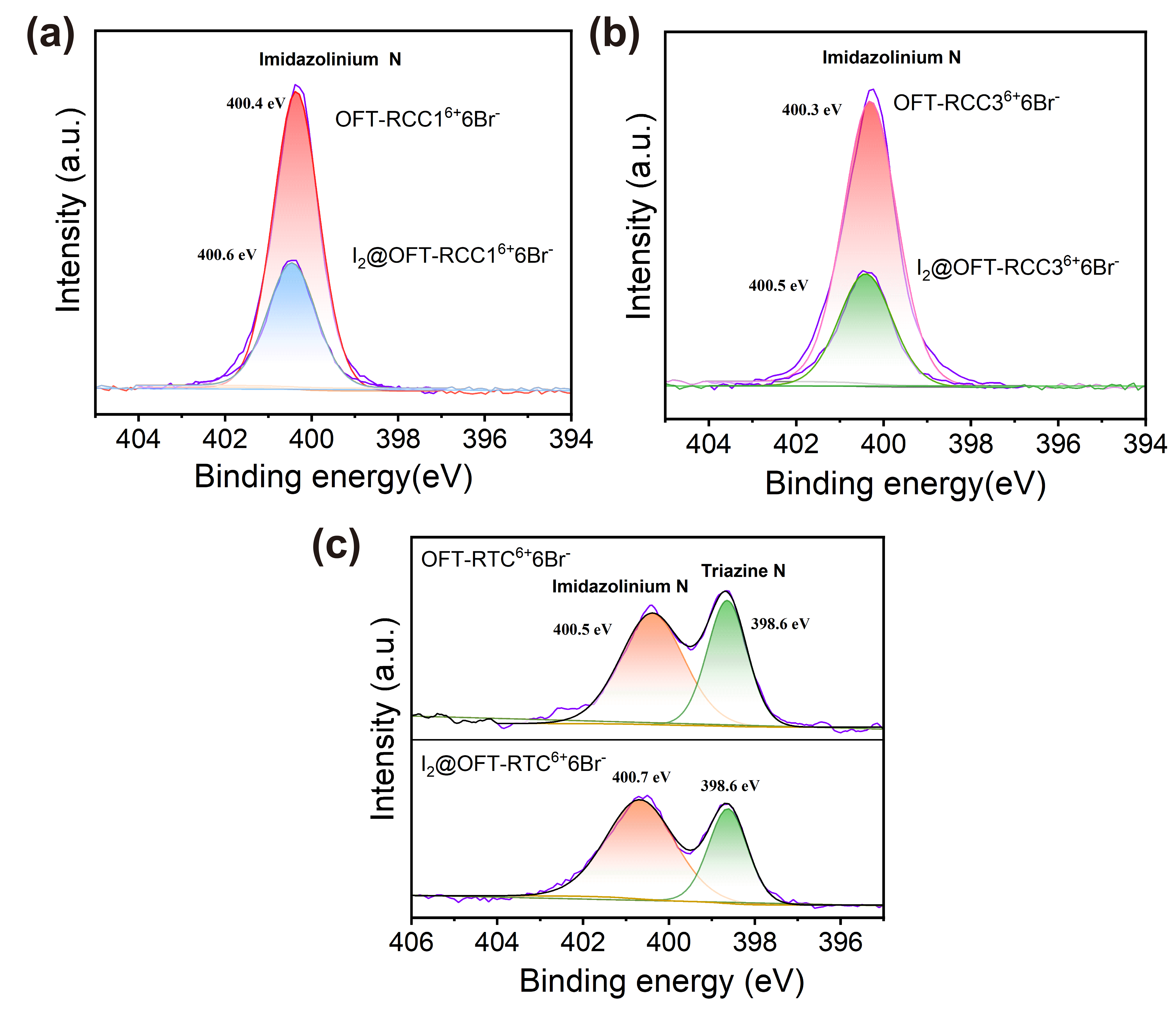
**

***Figure S45*** XPS analysis profiles of N 1s for **OFT-RCC1^6+^6Br^-^** (a), **OFT-RCC3^6+^6Br^-^** (b), **OFT-RTC^6+^6Br^-^** (c).

**4.2 Iodine release experiment**

To monitor the iodine release speed from iodine-loaded absorbents, time-dependent UV–vis spectroscopic measurements were carried out in methanol. In a typical experiment, **I_2_@OFT-RCC1^6+^6Br^-^**, **I_2_@OFT-RCC3^6+^6Br^-^** and **I_2_@OFT-RTC^6+^6Br^-^** (10 mg) was immersed in methanol (15.0 mL) in a reaction vial respectively. The UV–vis absorbance was recorded at different time intervals removing 3.0 mL methanol solution from the mother solution and then transferring it back. The time for release maximum was nearly 300 min with the release efficiency around 80 %, which distinctly indicated that the iodine is dissociating from the adsorbents.





***Figure S46*** Time-dependent UV/vis absorption spectra of iodine release of **I_2_@OFT-RCC1^6+^6Br^-^** (a), **I_2_@ OFT-RCC3^6+^6Br^-^** (b) and **I_2_@ OFT-RTC^6+^6Br^-^** (c). (d) Release efficiency of iodine from **I_2_@ adsorbents** in methanol over time.

**5. Iodine adsorption experiments in *n*-hexane solution**

Iodine solutions in *n*-hexane of different initial concentrations (0.1-1.0 mg mL^-1^, 15 mL) were prepared and materials (10 mg) were added to solutions. After 24 h of adsorption, adsorbents were removed by filtering and the UV-vis spectra of filtrate were carried out to calculate the equilibrium concentrations of remaining iodine. According to the pre-made standard calibration curve, the absorbance at 522 nm was chosen to determine the concentration of iodine in solution. The maximum adsorption capacity ($\text{q}_{\text{e}}\text{)}$ can be calculated using equation (S-3) and the efficiency of iodine removal (%) is determined by equation(S-4).

The maximum adsorption capacity calculation equation (S-3)

$$\text{q}_{\text{e}}\text{=}\text{(}\text{C}_{\text{0}}\text{-}\text{C}_{\text{e}}\text{)}\text{⋅}\frac{\text{v}}{\text{m}}$$

The removal efficiency calculation equation (S-4)

$$\text{removal efficiency=}\frac{\text{C}_{\text{0}}\text{-}\text{C}_{\text{e}}}{\text{C}_{\text{0}}}\text{×100\%}$$

Where m (mg) denotes the weight of adsorbents (**OFT-RCC1^6+^6Br^-^**, **OFT-RCC3^6+^6Br^-^**, **OFT-RTC^6+^6Br^-^**) and V (mL) represents the volume of iodine solutions in *n*-hexane. *C_0_* and *C_e_* (mg mL^-1^) are the initial and equilibrium concentrations of the iodine solutions, respectively.

The adsorption kinetics also was studied by immersing 10 mg of activated absorbents in 15mL of an iodine solution in *n*-hexane (1.0 mM) and monitoring the UV-Vis absorption spectra at various time intervals.

***
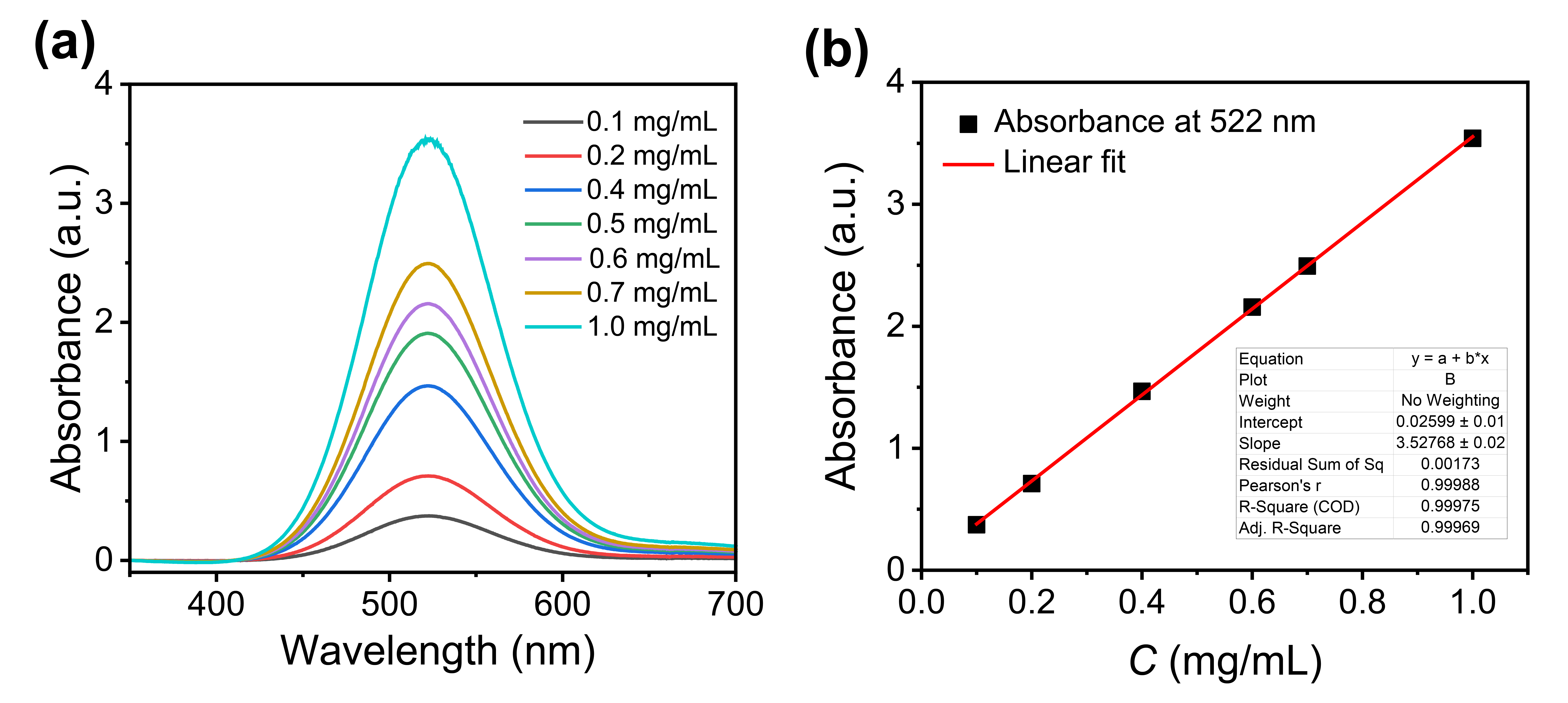
***

***Figure S47*** Standard calibration plot of iodine in *n*-hexane solution determined by UV-vis spectra.


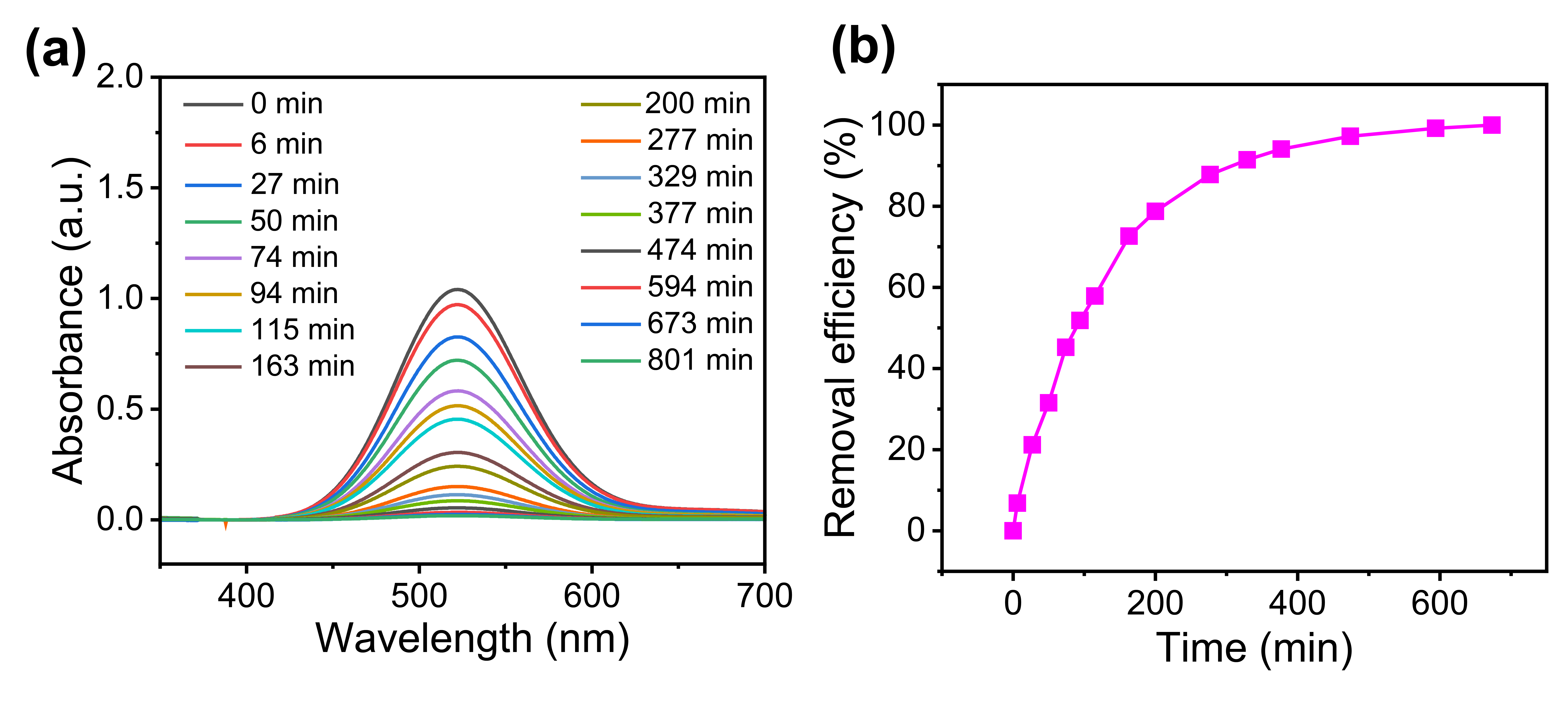


***Figure S48*** UV/Vis spectra of *n*-hexane iodine solution (1.0 mM) in the presence of **OFT-RCC1^6+^6Br^-^**. b) Kinetic of iodine adsorption by **OFT-RCC1^6+^6Br^-^** in *n*-hexane iodine solution (1.0 mM).


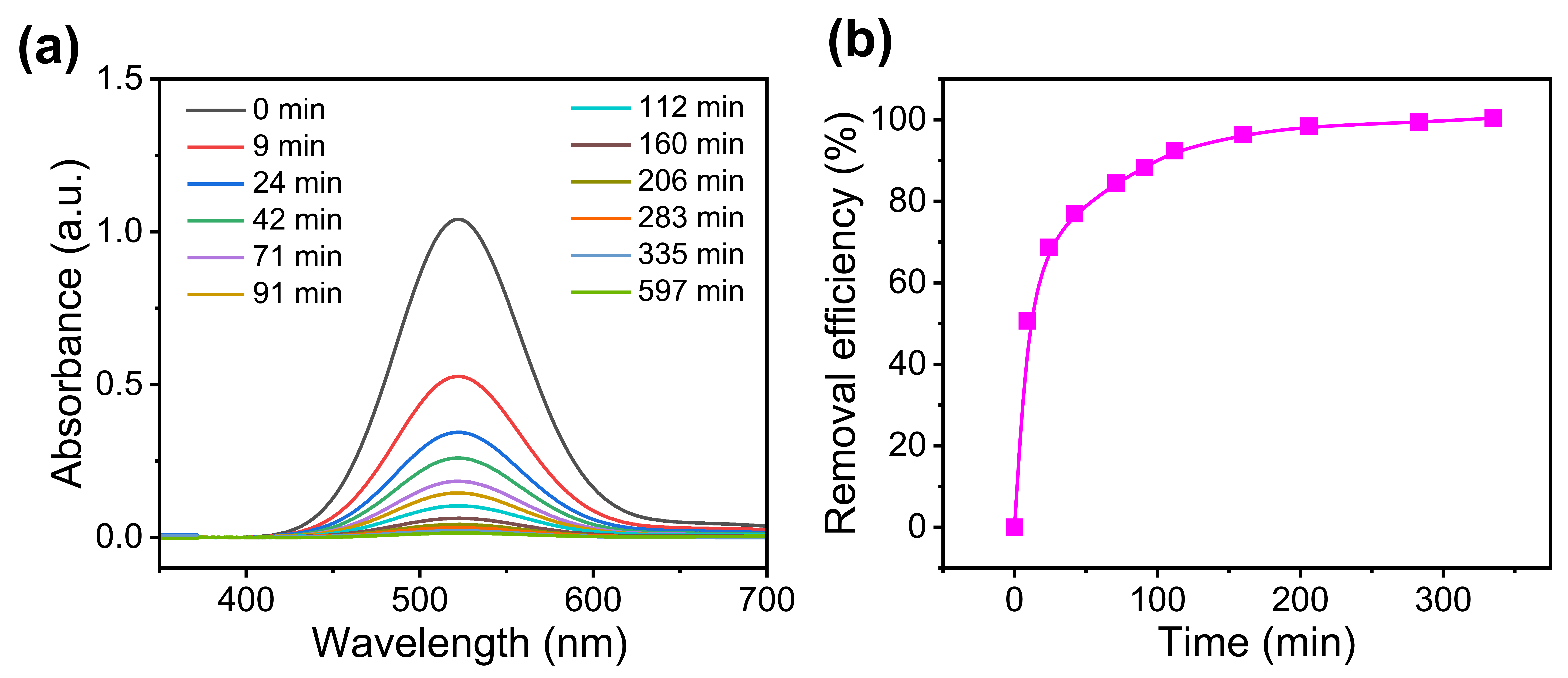


***Figure S49*** UV/Vis spectra of *n*-hexane iodine solution (1.0 mM) in the presence of **OFT-RCC3^6+^6Br^-^**. b) Kinetic of iodine adsorption by **OFT-RCC3^6+^6Br^-^** in *n*-hexane iodine solution (1.0 mM).

***
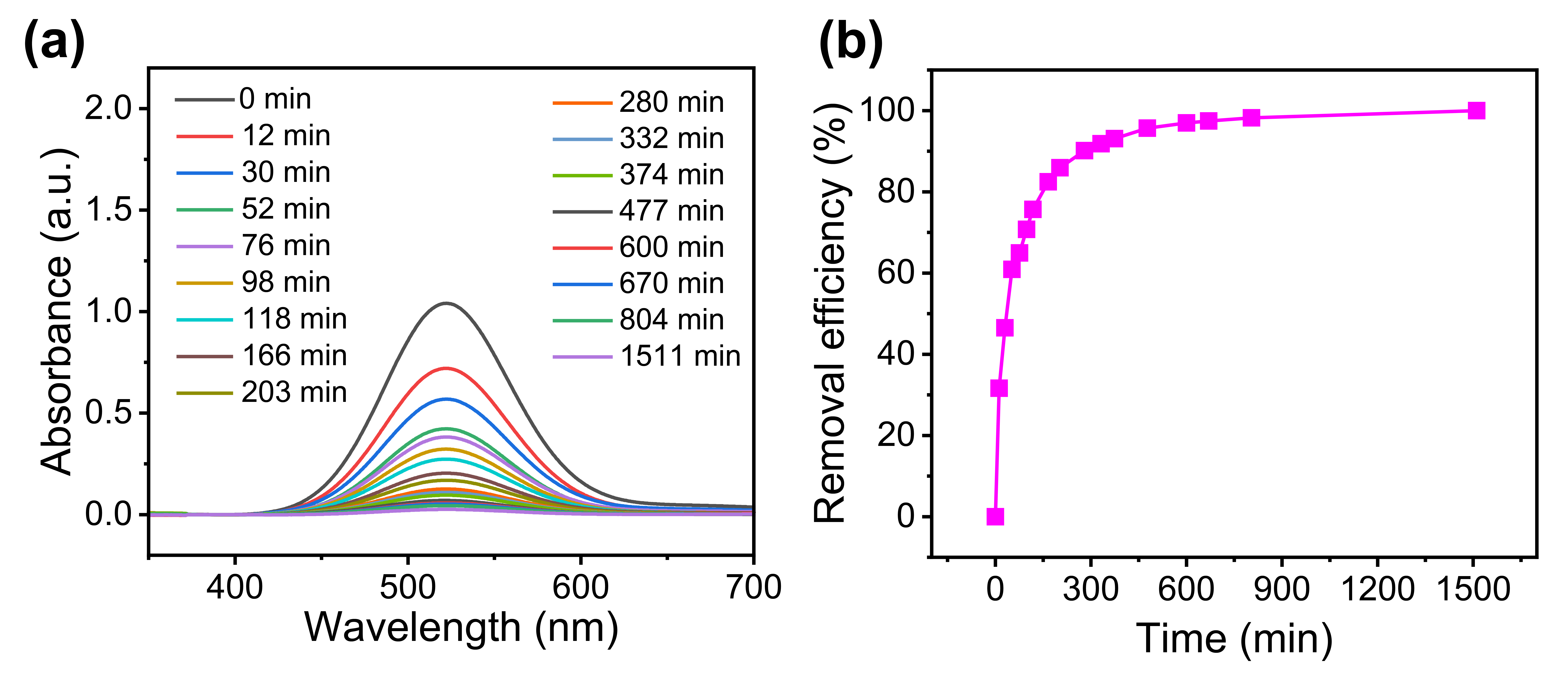
***

***Figure S50*** UV/Vis spectra of *n*-hexane iodine solution (1.0 mM) in the presence of **OFT-RTC^6+^6Br^-^**. b) Kinetic of iodine adsorption by **OFT-RTC^6+^6Br^-^** in *n*-hexane iodine solution (1.0 mM).


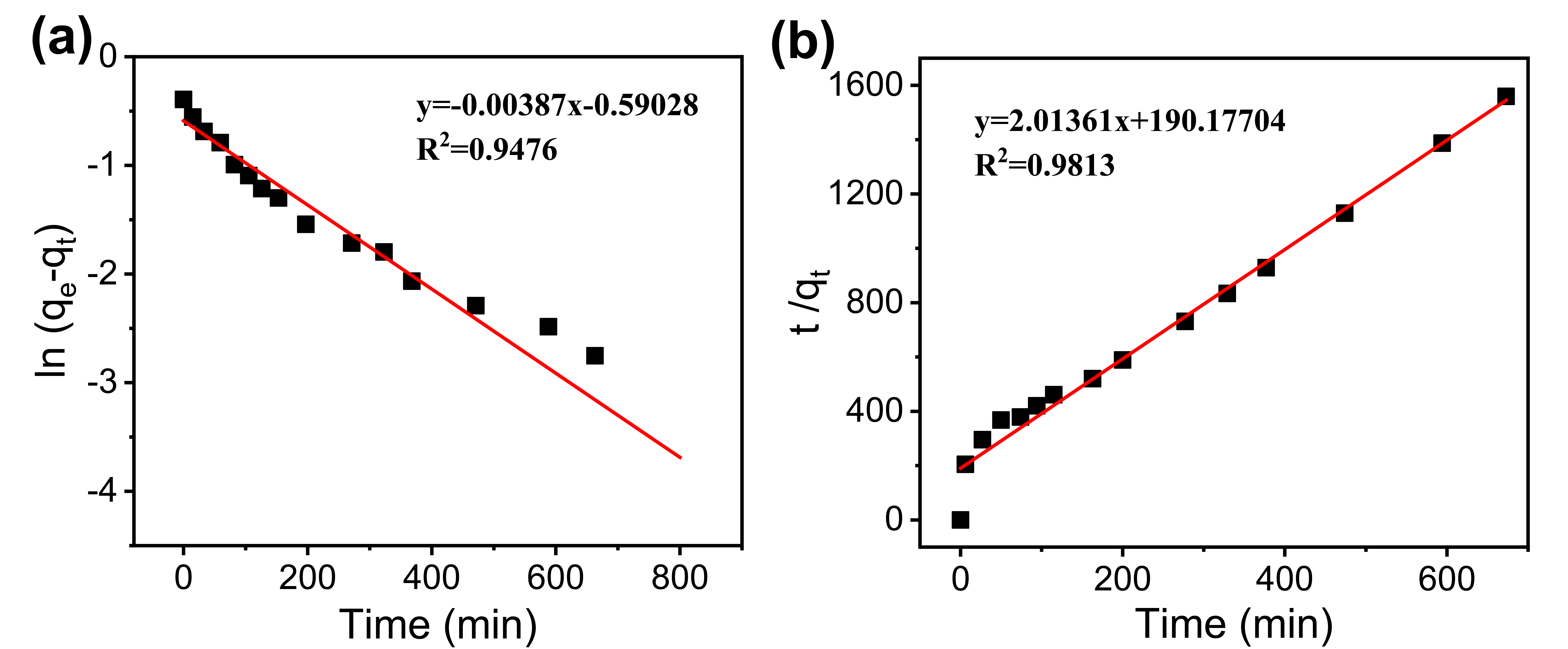


***Figure S51*** The kinetic fitting curves by pseudo-first-order kinetic (a) and pseudo-second-order model (b) for iodine *n*-hexane solution (1.0 mM) adsorption of **OFT-RCC1^6+^6Br^-^**.


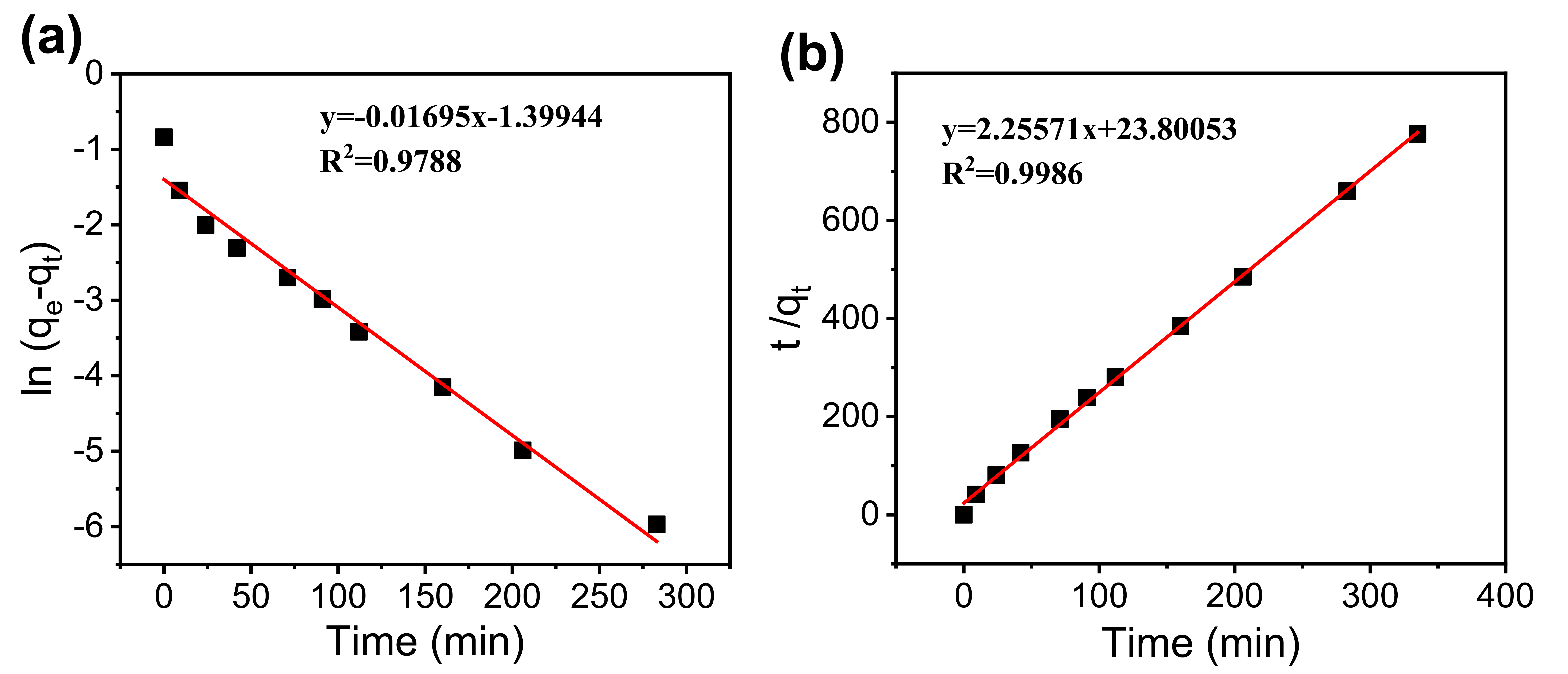


***Figure S52*** The kinetic fitting curves by pseudo-first-order kinetic (a) and pseudo-second-order model (b) for iodine *n*-hexane solution (1.0 mM) adsorption of **OFT-RCC3^6+^6Br^-^**.


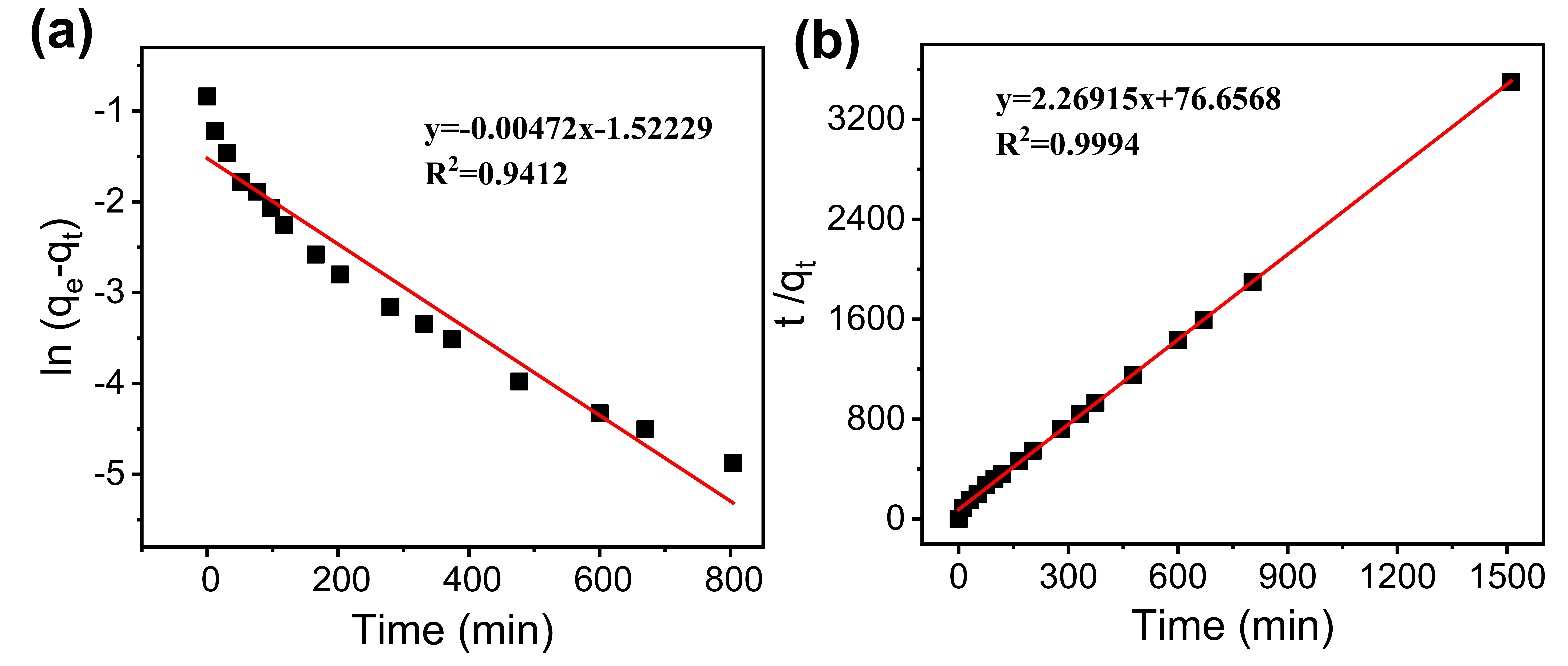


***Figure S53*** The kinetic fitting curves by pseudo-first-order kinetic (a) and pseudo-second-order model (b) for iodine *n*-hexane solution (1.0 mM) adsorption of **OFT-RTC^6+^6Br^-^****.**

***Table S5*** Kinetic parameters of I_2_ in *n*-hexane solution onto **OFT-RCC1^6+^6Br^-^**, **OFT-RCC3^6+^6Br^-^** and **OFT-RTC^6+^6Br^-^**.

| Adsorbent | Model | Parameters |  |
| --- | --- | --- | --- |
|  |  | q_exp_(g g^-1^) | 0.4316 |
|  |  | q_e,cal_ (g g^-1^) | 0.5542 |
|  | Pseudo-first-order | k_1_ (min^-1^) | 0.0039 |
| OFT-RCC1^6+^6Br^-^ |  | R^2^ | 0.9476 |
|  |  | q_e,cal_ (g g^-1^) | 0.4966 |
|  | Pseudo-second-order | k_2_ (g g^-1^min^-1^) | 0.0213 |
|  |  | R^2^ | 0.9813 |
|  |  | q_exp_(g g^-1^) | 0.4316 |
|  |  | q_e,cal_ (g g^-1^) | 0.2467 |
|  | Pseudo-first-order | k_1_ (min^-1^) | 0.0170 |
| OFT-RCC3^6+^6Br^-^ |  | R^2^ | 0.9788 |
|  |  | q_e,cal_ (g g^-1^) | 0.4433 |
|  | Pseudo-second-order | k_2_ (g g^-1^min^-1^) | 0.2138 |
|  |  | R^2^ | 0.9986 |
|  |  | q_exp_(g g^-1^) | 0.4316 |
|  |  | q_e,cal_ (g g^-1^) | 0.2182 |
|  | Pseudo-first-order | k_1_ (min^-1^) | 0.0047 |
| OFT-RTC^6+^6Br^-^ |  | R^2^ | 0.9412 |
|  |  | q_e,cal_ (g g^-1^) | 0.4407 |
|  | Pseudo-second-order | k_2_ (g g^-1^min^-1^) | 0.0672 |
|  |  | R^2^ | 0.9994 |

**6. Iodine adsorption experiments in aqueous solution**


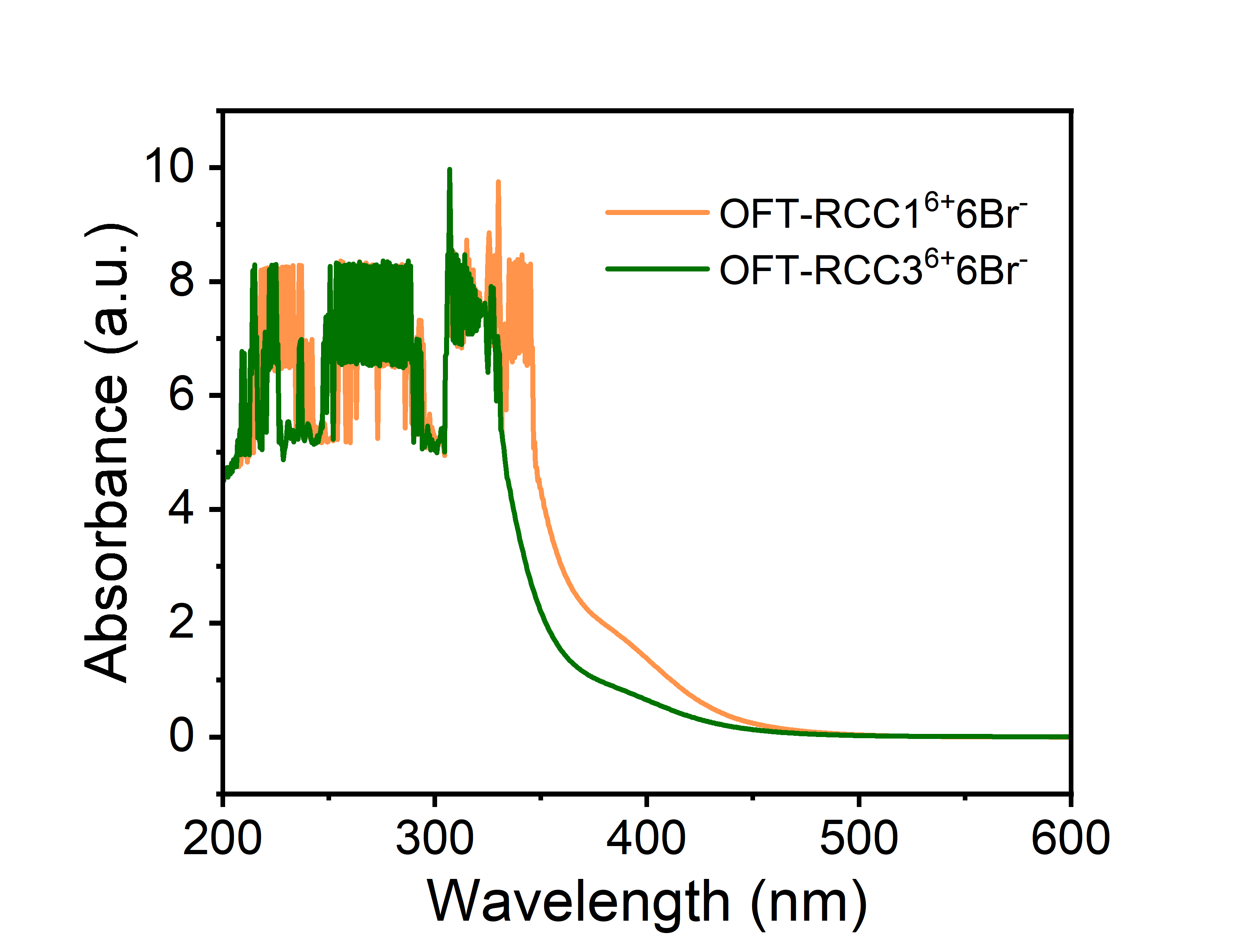


***Figure S54*** (a) UV/vis absorption spectra of aqueous solution of **OFT-RCC1^6+^6Br^-^** and **OFT-RCC3^6+^6Br^-^.**

**
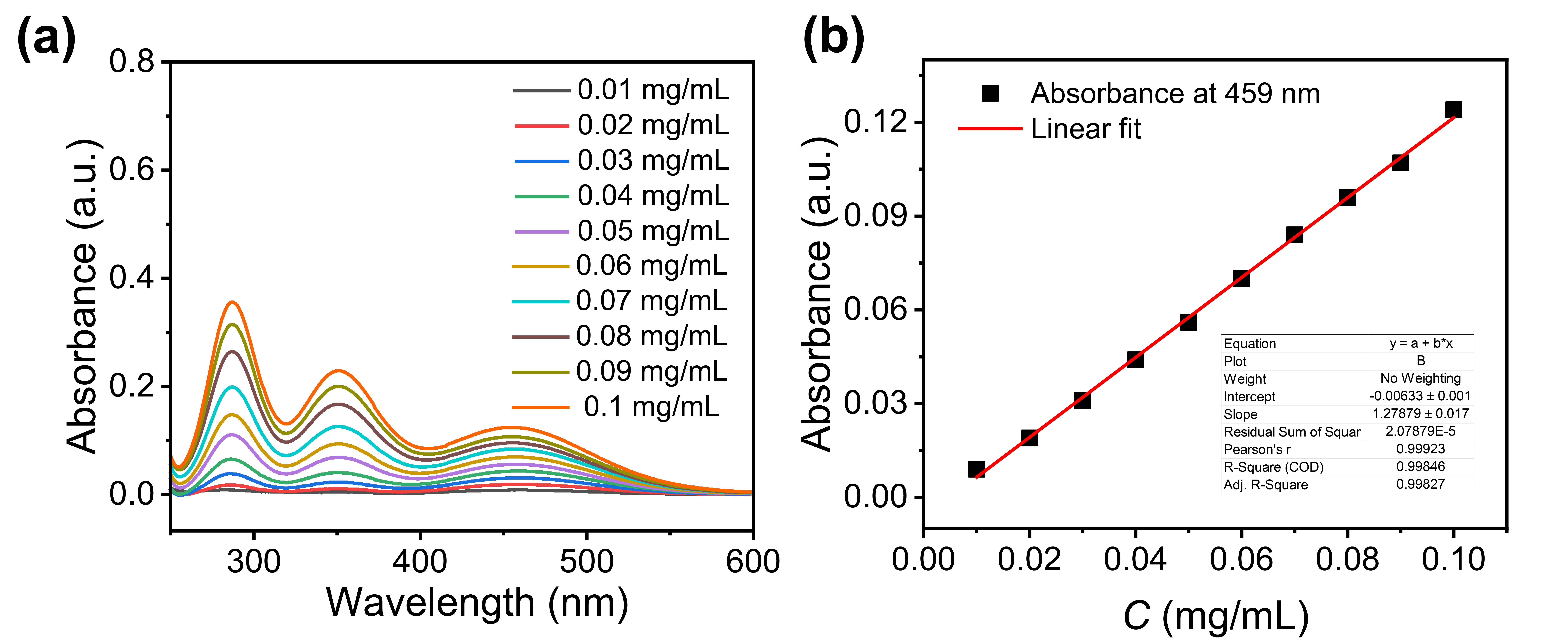
**

***Figure S55*** Standard calibration plot of iodine in aqueous solution determined by UV-vis spectra.

**
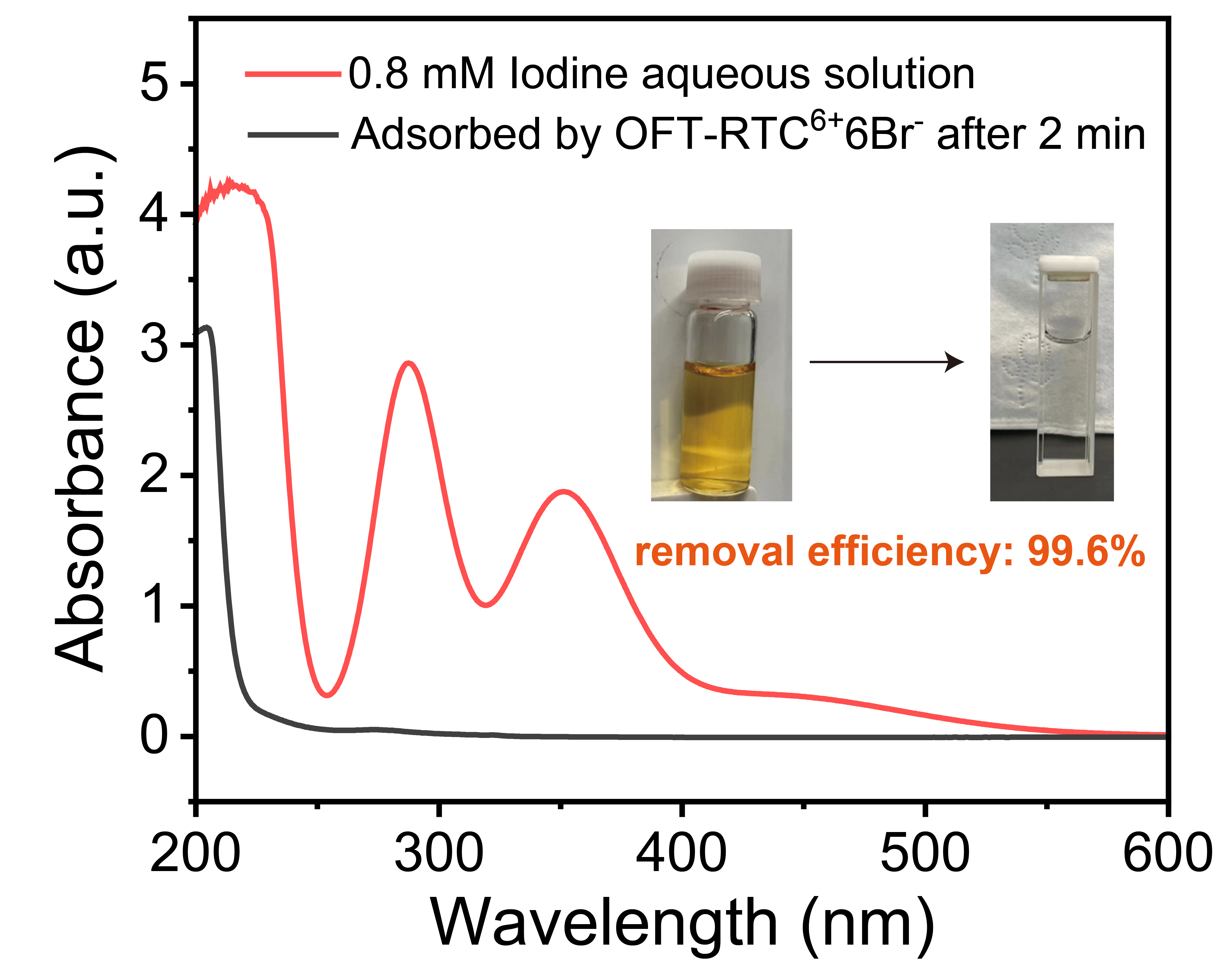
**

***Figure S56*** (a) UV/vis absorption spectra of aqueous solution of I_2_ (0.8 mM, 15 mL) upon addition of 5 mg **OFT-RTC^6+^6Br^-^.** The inset shows color changes of the solution upon the addition of **OFT-RTC^6+^6Br^-^** after 2 min.


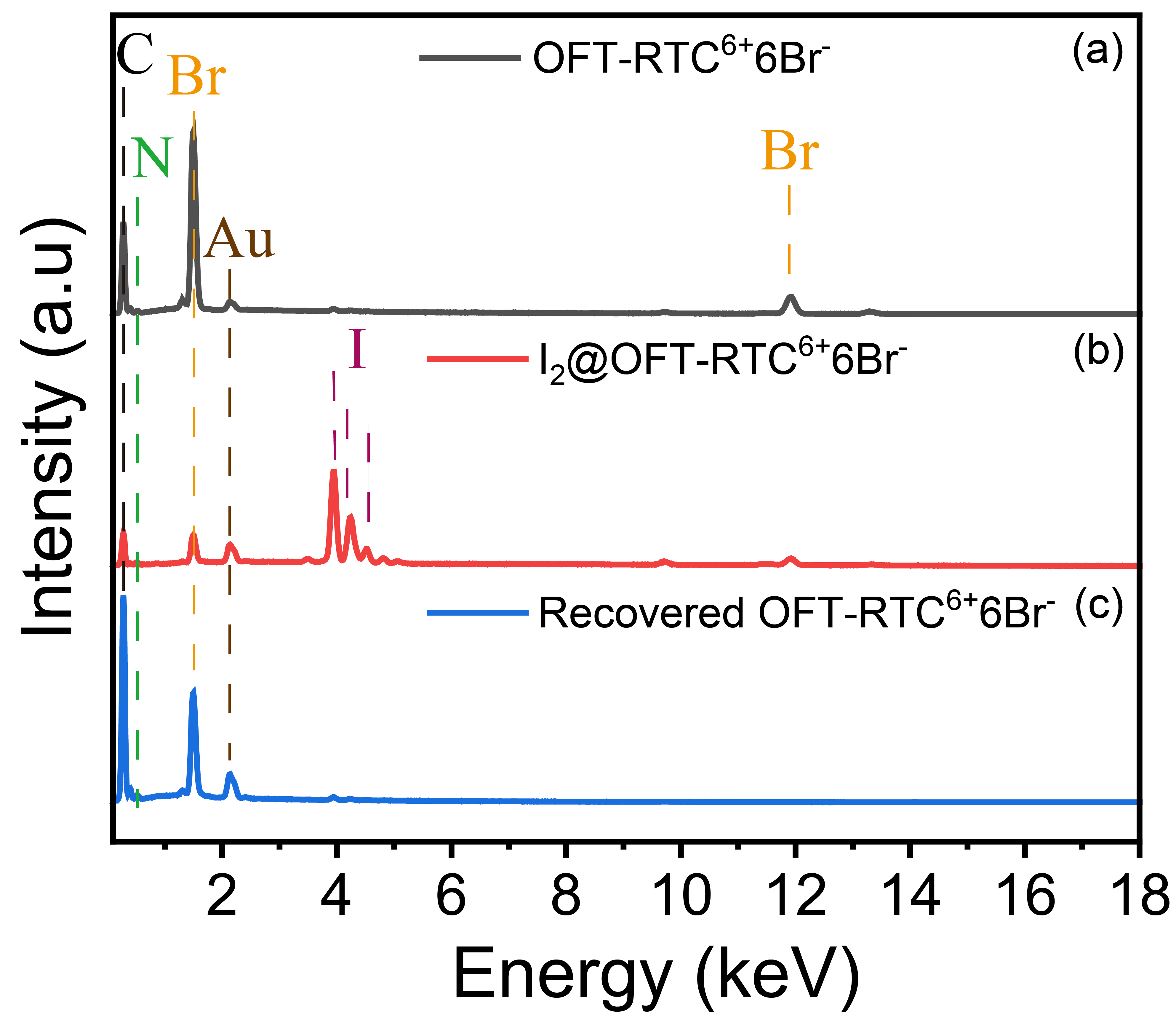


***Figure S57*** Energy-dispersive spectroscopy (EDS) analysis profiles of **OFT-RTC^6+^6Br^-^** before (a) and after (b) adsorption of iodine, and the EDS analysis profiles of recovered **OFT-RTC^6+^6Br^-^** after iodine release (c).

**7. Density functional theory (DFT) and** ***ab initio* molecular dynamics (AIMD) calculations**

The geometries of the complexes were fully optimized by means of the hybrid B3LYP functional^[10]^ D3 version of Grimme’s dispersion^[11]^. For all atoms, the def2-SVP basis set^[12]^ was applied. No geometry constraint was imposed during optimizations. The optimized geometries were verified as local minima on the potential energy surface by frequency computations at the same theoretical level. The energies of optimized geometries were further computed with def2-TZVP basis set^[12b]^. These calculations were performed with the Gaussian 16 suite of programs^[13]^. The binding energy (BE) was calculated using the equation of BE = E(total)-E(host)-E(guest).

We used the independent gradient model based on the Hirshfeld molecular density partitioning (IGMH)^[14]^ to evaluate the non-covalent interactions between the cage and iodine species. Wave function files were generated using single-point energy calculations. The IGMH calculations were performed using Multiwfn 3.8^[15]^, and the visualization was created with VMD 1.9.4a53^[16]^.


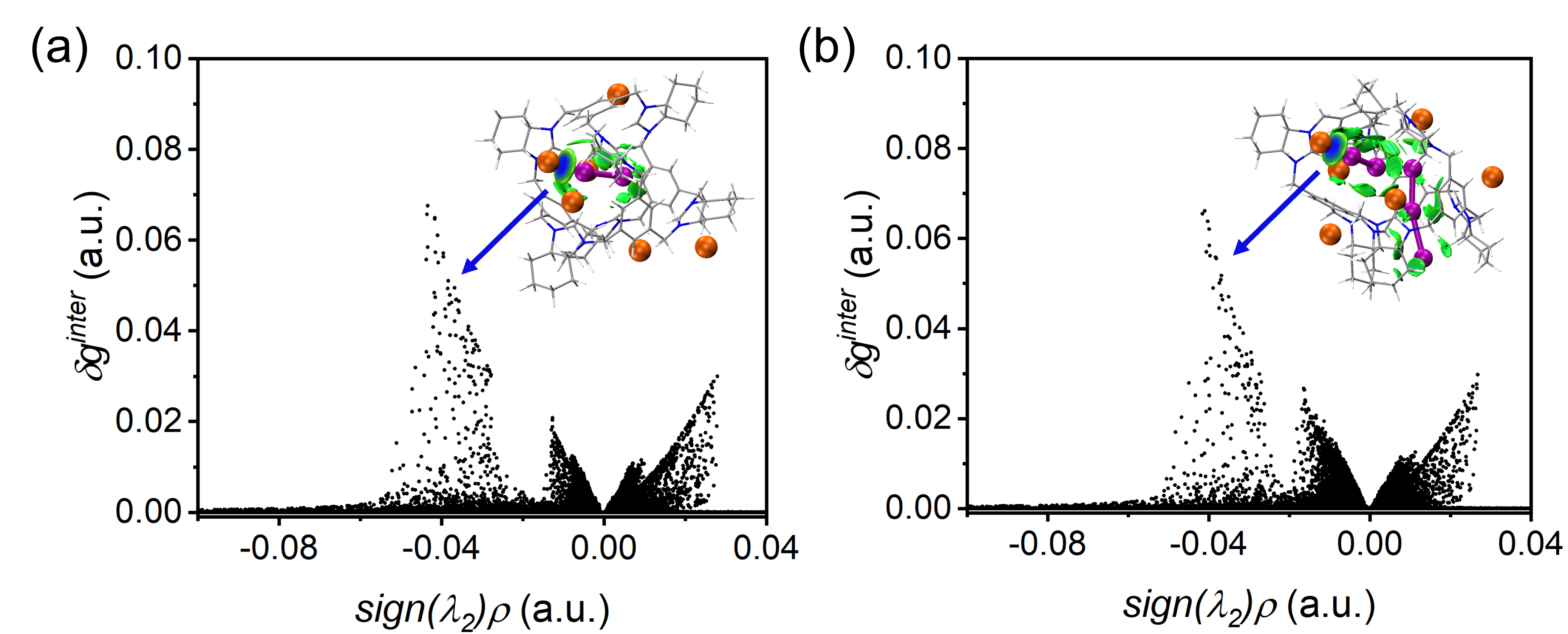


***Figure S58*** Scatter plots of IGMH for site 1: I_2__OFT-RCC3^6+^6Br^−^ and I_5__OFT-RCC3^6+^6Br^−^.

***Ab initio* molecular dynamics**

The geometry of the initial experimental structure was optimized prior to the ab initio molecular dynamics (AIMD) simulation. 1×1×2 and 2×1×1 supercells were used for OFT-RCC3^6+^6Br^-^ and (H_12_RCC3)^12+^12Br^-^, with an iodine molecule placed inside the cage cavity. This optimization used the Perdew-Burke-Ernzerhof (PBE) exchange correlation functional^[17]^ with DFT-D3(BJ) dispersion corrections^[18]^. The calculations were performed with the Quickstep module^[19]^ within CP2K^[20]^ using Goedecker-Teter-Hutter (GTH) pseudopotentials^[21]^ and TZVP-MOLOPT-PBE-GTH basis sets^[22]^. A plane-wave cut-off of 500 Ry was used together with a 4-level multigrid with a relative cut-off of 55 Ry and a multiplication factor of 3. Both cell dimensions and atomic coordinates were allowed to vary during the optimization.

Optimization was performed using the limited-memory Broyden-Fletcher-Goldfarb-Shanno (LBFGS) optimizer. Convergence was determined by the following criteria: the optimization was considered complete when the maximum change in geometry was less than 3×10^-3^ Bohr, the root mean square (RMS) deviation was less than 1.5×10^-3^ Bohr, the maximum force exerted on the atoms was less than 4.5×10^-4^ E_h_/Bohr and the RMS deviation of the forces was less than 3×10^-4^ E_h_/Bohr. Table S6 shows the optimized supercell parameters.

***Table S6***. The comparison of calculated and experimental lattice parameters.

| Supercells | a, b, c (Å) | α, β, γ (º) |
| --- | --- | --- |
| OFT-RCC3^6+^6Br^-^ | 21.419, 20.929, 28.124 | 80.52, 88.02, 118.22 |
| (H_12_RCC3)^12+^12Br^-^ | 29.495, 24.473, 24.847 | 90.01, 89.99, 89.94 |

AIMD simulations based on the optimised structures were performed using the CP2K package (CP2K Developers Group. CP2K^[20]^. The simulations were performed using an isotropic NPT ensemble. The functional, basis set and dispersion corrections were the same as those used for the structure optimization. The temperature was set to 348.15 K and the pressure to 1 atm. The canonical sampling by velocity rescaling (CSVR) thermostat^[23]^ was used. The total simulation time was 25 ps with a time step of 0.5 fs. The evolutions of potential energy (PE) are shown in Figure S59. Both the mean squared displacement (MSD) and the radial distribution function (RDF) g(r) for iodine in both cages were calculated using MDAnalysis^[24]^.


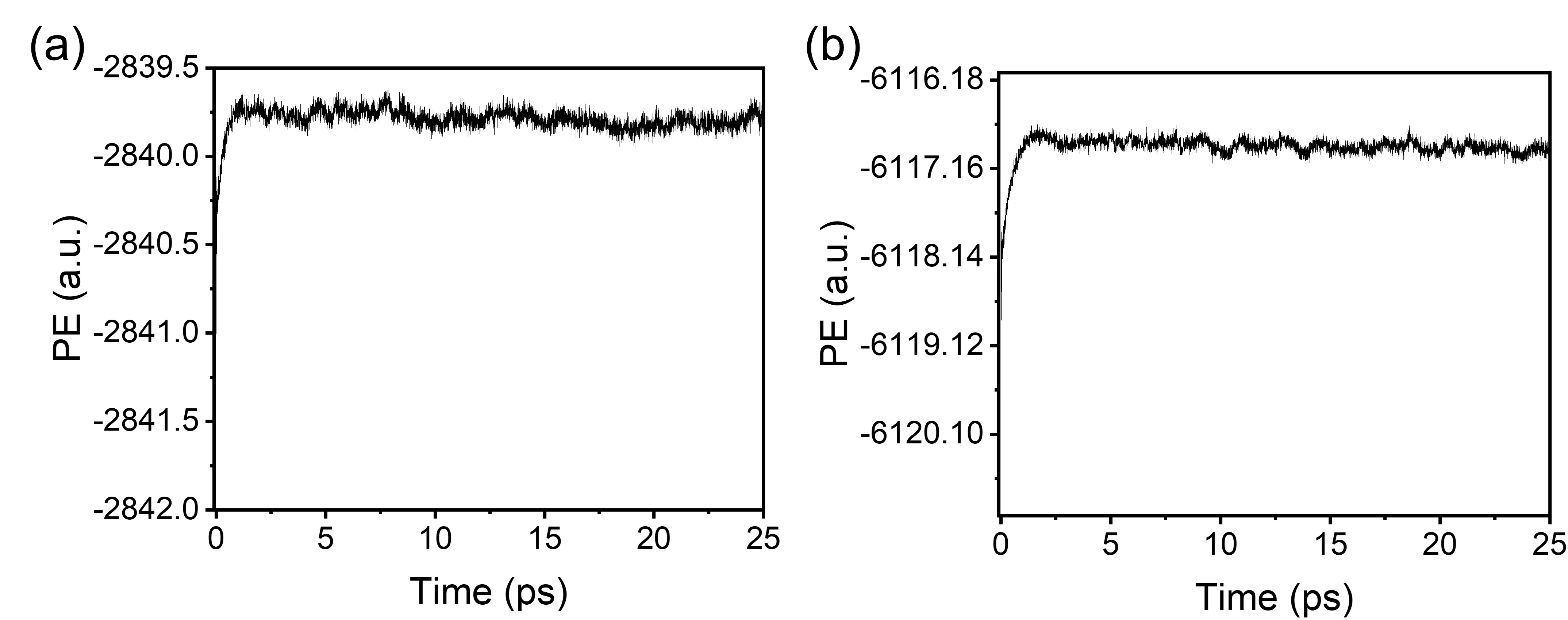


***Figure S59***. Evolution of the PE with time of (a) **OFT-RCC3^6+^6Br^-^** and (b) **(H_12_RCC3)^12+^12Br^-^**.

**8. Iodine adsorption performance comparison of two types of ionic cages**

A solution of I_2_ in *n*-hexane (1mg/ml, 3ml) was prepared, then 5mg **(H_12_RCC3)^12+^ 12Br^-^** 、 **(H_12_RTC)^12+^ 12Br^-^** 、 **(H_12_RCC1)^12+^ 12Br^-^** 、blank solution、**OFT-RCC1^6+^6Br^-^** 、 **OFT-RTC^6+^6Br^-^** 、**OFT-RCC3^6+^6Br^-^** were added and color changes were recorded at intervals over time.

***
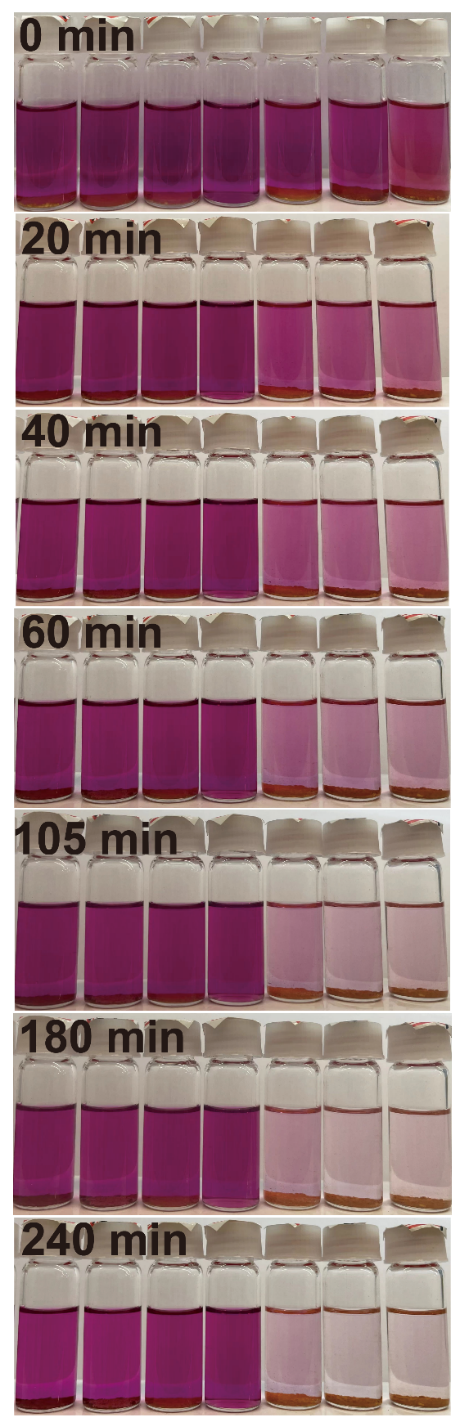
***

***Figure S60*** Time-dependent color changes of iodine *n*-hexane solution (3mL, 1mg/mL) upon addition of 5mg **(H_12_RCC3)^12+^ 12Br^-^**, **(H_12_RTC)^12+^ 12Br^-^**, **(H_12_RCC1)^12+^ 12Br^-^**, blank solution, **OFT-RCC1^6+^6Br**, **OFT-RTC^6+^6Br^-^** and **OFT-RCC3^6+^6Br^-^** from left to right.

**9. References**

[1] Bruker AXS Inc., Madison, Wisconsin, USA.

[2] a) G. Sheldrick, *Acta Crystallogr. C* **2015**, *71*, 3; b) G. Sheldrick, *Acta Crystallogr. A* **2015**, *71*, 3.

[3] O. V. Dolomanov, L. J. Bourhis, R. J. Gildea, J. A. K. Howard, H. Puschmann, *J. Appl. Crystallogr.* **2009**, *42*, 339.

[4] C. R. Groom, I. J. Bruno, M. P. Lightfoot, S. C. Ward, *Acta Crystallogr. B* **2016**, *72*, 171.

[5] D. Kratzert, https://dkratzert.de/finalcif.html.

[6] S. I. Swamy, J. Bacsa, J. T. A. Jones, K. C. Stylianou, A. Steiner, L. K. Ritchie, T. Hasell, J. A. Gould, A. Laybourn, Y. Z. Khimyak, D. J. Adams, M. J. Rosseinsky, A. I. Cooper, *J. Am. Chem. Soc.* **2010**, *132*, 12773.

[7] M. Liu, M. A. Little, K. E. Jelfs, J. T. A. Jones, M. Schmidtmann, S. Y. Chong, T. Hasell, A. I. Cooper, *J. Am. Chem. Soc.* **2014**, *136*, 7583.

[8] H. Ding, Y. Yang, B. Li, F. Pan, G. Zhu, M. Zeller, D. Yuan, C. Wang, *Chem. Commun.* **2015**, *51*, 1976.

[9] Y.-z. Ye, H.-y. Liu, Y.-j. Gong, Z.-z. Xu, Y. Zhao, N. Yu, Q.-s. Wang, W. Wen, T.-y. Yang, W. Li, S. Jiang, *Chem* **2024**, *10*, 1118.

[10] a) P. J. Stephens, F. J. Devlin, C. F. Chabalowski, M. J. Frisch, *J. Phys. Chem. C* **1994**, *98*, 11623; b) K. Kim, K. D. Jordan, *J. Phys. Chem. C* **1994**, *98*, 10089.

[11] S. Grimme, J. Antony, S. Ehrlich, H. Krieg, *J. Chem. Phys.* **2010**, *132*, 154104.

[12] a) F. Weigend, *Phys. Chem. Chem. Phys.* **2006**, *8*, 1057; b) F. Weigend, R. Ahlrichs, *Phys. Chem. Chem. Phys.* **2005**, *7*, 3297.

[13] M. J. Frisch, G. W. Trucks, H. B. Schlegel, G. E. Scuseria, M. A. Robb, J. R. Cheeseman, G. Scalmani, V. Barone, G. A. Petersson, H. Nakatsuji, X. Li, M. Caricato, A. V. Marenich, J. Bloino, B. G. Janesko, R. Gomperts, B. Mennucci, H. P. Hratchian, J. V. Ortiz, A. F. Izmaylov, J. L. Sonnenberg, Williams, F. Ding, F. Lipparini, F. Egidi, J. Goings, B. Peng, A. Petrone, T. Henderson, D. Ranasinghe, V. G. Zakrzewski, J. Gao, N. Rega, G. Zheng, W. Liang, M. Hada, M. Ehara, K. Toyota, R. Fukuda, J. Hasegawa, M. Ishida, T. Nakajima, Y. Honda, O. Kitao, H. Nakai, T. Vreven, K. Throssell, J. A. Montgomery Jr., J. E. Peralta, F. Ogliaro, M. J. Bearpark, J. J. Heyd, E. N. Brothers, K. N. Kudin, V. N. Staroverov, T. A. Keith, R. Kobayashi, J. Normand, K. Raghavachari, A. P. Rendell, J. C. Burant, S. S. Iyengar, J. Tomasi, M. Cossi, J. M. Millam, M. Klene, C. Adamo, R. Cammi, J. W. Ochterski, R. L. Martin, K. Morokuma, O. Farkas, J. B. Foresman, D. J. Fox, Wallingford, CT, **2016**.

[14] T. Lu, Q. Chen, *J. Comput. Chem.* **2022**, *43*, 539.

[15] T. Lu, F. Chen, *J. Comput. Chem.* **2012**, *33*, 580.

[16] D. A. Cosgrove, P. W. Kenny, *J. Mol.Model* **1996**, *14*, 1.

[17] J. P. Perdew, K. Burke, M. Ernzerhof, *Phys. Rev. Lett.* **1996**, *77*, 3865.

[18] S. Grimme, S. Ehrlich, L. Goerigk, *J. Comput. Chem.* **2011**, *32*, 1456.

[19] J. VandeVondele, M. Krack, F. Mohamed, M. Parrinello, T. Chassaing, J. r. Hutter, *Comput. Phys. Commun.* **2005**, *167*, 103.

[20] T. D. Kühne, M. Iannuzzi, M. Del Ben, V. V. Rybkin, P. Seewald, F. Stein, T. Laino, R. Z. Khaliullin, O. Schütt, F. Schiffmann, D. Golze, J. Wilhelm, S. Chulkov, M. H. Bani-Hashemian, V. Weber, U. Borštnik, M. Taillefumier, A. S. Jakobovits, A. Lazzaro, H. Pabst, T. Müller, R. Schade, M. Guidon, S. Andermatt, N. Holmberg, G. K. Schenter, A. Hehn, A. Bussy, F. Belleflamme, G. Tabacchi, A. Glöß, M. Lass, I. Bethune, C. J. Mundy, C. Plessl, M. Watkins, J. VandeVondele, M. Krack, J. Hutter, *J. Chem. Phys.* **2020**, *152*, 194103.

[21] S. Goedecker, M. Teter, J. Hutter, *Physical Review B* **1996**, *54*, 1703.

[22] J. VandeVondele, J. r. Hutter, *J. Chem. Phys.* **2007**, *127*, 114105.

[23] G. Bussi, D. Donadio, M. Parrinello, *J. Chem. Phys.* **2007**, *126*, 014101.

[24] a) N. Michaud-Agrawal, E. J. Denning, T. B. Woolf, O. Beckstein, *J. Comput. Chem.* **2011**, *32*, 2319; b) R. J. Gowers, M. Linke, J. Barnoud, T. J. E. Reddy, M. N. Melo, S. L. Seyler, J. DomaإÑski, D. L. Dotson, S. b. Buchoux, I. M. Kenney, O. Beckstein, in *Proceedings of the 15th Python in Science Conference* (Eds.: S. Benthall, S. Rostrup), pp. 98
